# Regulation Transcriptional of Antibiotic Resistance Genes (ARGs) in Bacteria Isolated from WWTP

**DOI:** 10.1007/s00284-023-03449-z

**Published:** 2023-09-06

**Authors:** Grethel Díaz-Palafox, Yahaira de Jesús Tamayo-Ordoñez, Juan Manuel Bello-López, Benjamin Abraham Ayil-Gutiérrez, Mónica Margarita RodrÍguez-Garza, José Antonio Rodríguez-de la Garza, Gerardo de Jesús Sosa-Santillán, Erika Acosta-Cruz, Alejandro Ruiz-Marín, Atl Victor Córdova-Quiroz, Luis Jorge Pérez-Reda, Francisco Alberto Tamayo-Ordoñez, Maria Concepción Tamayo-Ordoñez

**Affiliations:** 1 Laboratorio de Ingeniería Genética, Departamento de Biotecnología, Facultad de Ciencias Químicas, Universidad Autónoma de Coahuila, Ing J. Cárdenas Valdez s/n, República, 25280 Saltillo, Coah Mexico; 2 Laboratorio de Biotecnología Ambiental del Centro de Biotecnología Genómica del Instituto Politécnico Nacional, CP 88710 Reynosa, TAMPS México; 3 División de Investigación, Hospital Juárez de México, Mexico City, Mexico; 4 CONACYT- Centro de Biotecnología Genómica, Instituto Politécnico Nacional, Biotecnologia Vegetal, Blvd. del Maestro, s/n, Esq. Elías Piña, 88710 Reynosa, Mexico; 5 Laboratorio de Biotecnología Ambiental, Departamento de Biotecnología, Facultad de Ciencias Químicas, Universidad Autónoma de Coahuila, Ing J. Cárdenas Valdez s/n, República, 25280 Saltillo, Coah Mexico; 6 Laboratorio de Biosíntesis Enzimática, Departamento de Biotecnología, Facultad de Ciencias Químicas, Universidad Autónoma de Coahuila, Ing J. Cárdenas Valdez s/n, República, 25280 Saltillo, Coah Mexico; 7 Laboratorio de Microbiología Molecular, Departamento de Biotecnología, Facultad de Ciencias Químicas, Universidad Autónoma de Coahuila, Ing J. Cárdenas Valdez s/n, República, 25280 Saltillo, Coah Mexico; 8 Facultad de Química, Universidad Autónoma del Carmen, Campus “General José Ortiz Ávila, Calle 56 No. 4, 24180 Carmen, Campeche Mexico

## Abstract

The incidence of antibiotics and transcriptional regulation of ARGs in isolated bacteria from wastewater needs to be explored. By HPLC, in samples of untreated wastewater, ampicillin (49.74 ± 5.70 µg/mL), chloramphenicol (0.60 ± 0.03 µg/mL), tylosin (72.95 ± 2.03 µg/mL), and oxytetracycline (0.22 ± 0.01 µg/mL) was determined. Through metagenomic analysis identified 58 bacterial species belonging to 9 phyla and at least 14 species have shown resistance to a variety of antibiotics. Twenty-two bacterial isolates were proved to be resistant to fifteen antibiotics of new generation and used in medical research to combat infectious diseases. Fourteen strains were shown to harbor plasmids in size ranges of 2–5 Kb, 6–10 Kb and plasmids with size greater than 10 Kb. By quantitative PCR it was possible to identify genes *sul*, *qnr*, *cat1*, *aadA1,* and *sat-1* gene were shown to be present in gDNA samples from treated and untreated samples of wastewater and by relative expression analysis, differential expression of *cat1*, *ermB*, *act,* and *tetA* genes was demonstrated in strains that showed identity with *Escherichia coli*, *Bacteroides fragilis,* and *Salmonella thyphi*, and that were stressed with different concentrations of antibiotics. The presence of ARGs in untreated water samples, as well as in bacterial isolates, was indicative that in these habitats there are microorganisms that can resist β-lactams, aminoglycosides, tetracyclines, sulfonamides, and quinolones.

**Supplementary Information:**

The online version contains supplementary material available at 10.1007/s00284-023-03449-z.

## Introduction

Bacteria during their evolution have developed genetic mechanisms allowing them to resist different antibiotics [1]. This may be intrinsically resistant to one or more antibiotics or acquire resistance via mutations or horizontal gene transfer and through cellular mechanisms involving efflux proteins [2, 3]. Porins are outer membrane proteins associated with the modulation of cellular permeability. The OmpF-defective mutant of *E. coli* was shown to be resistant to several antibiotics, suggesting that OmpF functions as the main route of outer membrane penetration for many antibiotics [3]. Depending on the pressure of the antibiotic on the bacteria, it may use different cellular mechanisms to resist them. For example, before the resistance to tetracycline and chloramphenicol, they are associated with the presence of efflux proteins [4, 5].

Also, the selective pressure on microbial communities due to the use of antibiotics led bacteria to improve their antibiotic resistance mechanisms to ensure their survival [2, 6]. Thus, even though various antibiotics have been developed for their use in medical treatment, new generation antibiotic-resistant pathogenic bacteria have evolved becoming a public health issue due to longer hospitalization, which besides implying a rise in the cost of treatment, might also raise the death toll from infection with these adapted pathogens [7]; a challenge that requires both research efforts and corresponding public health policy development [8].

The rapid evolution of antibiotic-resistant bacteria can be influenced by selection driven by the known presence of antibiotics in rivers, sewage, and wastewater treatment plants (WWTPs) [9, 10]. Chow et al. (2021) [11] reviewed the literature on antibiotic detection in water and sediment samples through HPLC. The authors found 40 papers published between 1999 and 2018 containing 887 environmental antibiotic concentration reports from sites in Europe, Asia, and North America, 212 from sediment and 675 from aquatic environments. According to the authors, 2% of these reports overlap the minimum inhibitory concentration (MIC) ranges known for wild-type bacteria, thus creating a strong selection pressure on antibiotic resistance. In a significant number of reports, the antibiotic concentrations reported are above the minimum selective concentration (MSC) estimated to be between 1/4 and 1/230 of the MIC [12, 13], which could affect biological processes like transcription, recombination rates, mutation, and horizontal gene transfer.

The need to eliminate or reduce the global emergence of antibiotic resistance favored by the presence in the environment of antimicrobial pharmaceuticals has generated current research to address the issue through the development of new technologies and strategies. Within these developments, the application of recombinant DNA technology in wastewater treatment has had little advance [14, 15], due to the possible consequences on native and non-pathogenic microorganisms present in the environment. To minimize these negative effects, it is necessary to genetically characterize the microorganisms found in the water or sediment samples to be treated. The genetic characterization of the presence of antibiotic-resistant microorganisms and knowledge of the transcription of genetic elements responsible for their resistance, for example, ARGs (Antibiotic resistance genes), will allow the development of innovative technologies aimed at a group of microbial communities, thus minimizing the adverse effects of recombinant DNA technologies.

In WWTPs are present bacteria of both human and environmental origins and antibiotic residues from households, hospitals, and small-scale pharmaceutical industries [16, 17]. Several studies have suggested a close relationship between antibiotic identification, ARGs expression from WWTP samples, and the incidence of antibiotic-resistant bacteria [18, 19]. It is suggested that the high bacterial density, nutrient availability in conditions of stress a variety of antibiotics present in wastewater, provides suitable conditions to facilitate a high rate of horizontal gene transfer among environmental bacteria and human pathogens [20]. Liu et al. (2019) [18], using metagenomic and metatranscriptomic identified diversity, abundance, 360 ARGs associated with 24 classes of antibiotics in activated sludge (AS) from three conventional WWTPs Taiwan. Metatranscriptome analysis showed 65.8% of the identified ARGs were being expressed and 110 ARGs were annotated as plasmid-associate.

Also, Xu et al. (2020) [21] using transcriptional analysis, demonstrated that 202 ARGs transcripts were detected from samples of sewage sludge from wastewater. ARGs transcripts more relevant were *qacEdelta1*-02, s*ul2*, *qacEdelta1*-01, *aadA2*-03, and *tetX*. Differences in the regulation of these ARGs was demonstrated, depending on the year's season, where there were lower abundances in summer and winter, demonstrating that bacterial communities with antibiotic resistance can change the ARGs transcription according to abiotic factors.

Therefore, if research is carried out aimed at understanding the genomics and transcriptomics of ARGs present in antibiotic-resistant bacteria isolated from WWTP and through advances in genetic engineering, it is possible to innovate a strategy aimed at eliminating antibiotic resistance in bacteria present in wastewater [17].

So due to the importance of the presence of antibiotics and antibiotic-resistant microorganisms in WWTPs, in this research we identified antibiotic-resistant bacteria present in samples from the WWTP through metagenomic analysis, identifying a total of 58 bacteria from untreated waters. At least 22 strains were isolated that proved multidrug-resistant, differentially expressing ARGs related to resistance to ampicillin, tylosin, chloramphenicol, and oxytetracycline and harbor plasmids ranging in size from 2 to 10 kb were identified.

Future genetic characterization of these plasmids could help us innovate new recombinant DNA strategies that allow deleting the resistance cassettes in bacteria present in WWTPs and thus help the treatment of untreated water.

## Materials and Methods

### Sample Collection and Analysis of Physicochemical Parameters

Water samples were taken at the inlet and outlet of the Saltillo wastewater treatment plant located at Dámaso Rodríguez González 750, Nuevo Centro Metropolitano, Saltillo, Coahuila. Sampling areas with homogeneous-looking water where chosen and 100 mL of water were collected in three points in triplicate.

Total alkalinity (CaCO_3_ mg/L), total hardness (CaCO_3_ mg/L), chlorides (mg/L), dissolved oxygen (mg/L), and biochemical oxygen demand (BOD mg/L) were evaluated according to the methods in the Official Mexican Standards NMX-AA-036-SCFI-2001, NMX-AA-072-SCFI-2001, NMX-AA-073-SCFI-2001, NMX-AA-028-SCFI-2001, and NMX- AA-028-SCFI-2001, respectively.

### Determination of Ampicillin, Chloramphenicol, Oxytetracycline, and Tylosin in Water Samples

A Thermo HPLC instrument coupled with a UV detector (P1500 pump, UV2000 detector, AS3000 autosampler) was employed to determine ampicillin, chloramphenicol, oxytetracycline, and tylosin using a C-18 column. The system used to detect the compounds consisted of a mobile phase of 70:30 CH3OH:H2O at a 0.3 mL min^−1^ flow rate, and a detection wavelength of 254 nm, 271 nm, 280 nm, and 351 nm for ampicillin, chloramphenicol, tylosin, and oxytetracycline. Commercial products (chloramphenicol and ampicillin from AMSA laboratories and oxytetracycline and tylosin from veterinary pharmaceutical suppliers), derived from the fact that these are used in veterinary and human medicine were used, and there is a high probability of finding them in urban wastewater. The inlet and outlet water samples from the treatment water network were analyzed in triplicate and the mean value was plotted and subjected to a Tukey test (α = 0.05).

### Metagenomic Analysis of Samples

gDNA was extracted with the Plant/Fungi DNA Isolation kit (Norgen Biotek Corp.). The purity and integrity of the gDNA was verified by electrophoresis in 1% agarose gel and measured in a NanoDrop 2000 microvolume spectrophotometer.

Samples were analyzed by 16S rDNA sequencing using the ABI PRISM model 377 automatic sequencer (PerkinElmer Inc., USA). The sequencing was performed using 200 ng of the gDNA used in the PCR reaction in a Sequenase v.2 Kit thermocycler (Amersham, U.S. Biochemicals). The reaction mix consisted of 4 µL of 5X Big dye Buffer v3.1, 10 µL Sterile milliQ water, 1 µL of 5 mM FW or RV initiator, 4 µL of Big Dye v3.1 ready mix, in a final volume of 20 µL. Subsequently, it was amplified by PCR under the appropriate conditions for each gene under study. Subsequently, 10 µL of the PCR product was purified, 10 µL of X-terminator, and 45 µl of SAM were added. The mixture was left to incubate for 30 min at 37 °C with shaking at 13,000 rpm. Subsequently, it was centrifuged for 2 min at 13,000 rpm. The supernatant was transferred to another tube and 20 µL was taken for sequencing. The obtained readings were identified online by Nucleotide BLAST (https://blast.ncbi.nlm.nih.gov/Blast.cgi) and by scanning the SILVA high-quality ribosomal RNA databases (http://www.arb-silva.de), the Michigan State University Ribosomal Database Project (RDP; https://rdp.cme.msu.edu/index.jsp), the EzTaxon sequence collection (http://www.ezbiocloud.net/eztaxon), and the NCBI non-redundant database (http://www.ncbi.nlm.nih.gov/guide/all/#databases) containing sequence collections from GenBank, the European Bioinformatics Institute (EMBL-EBI), the DNA Data Bank of Japan (DDBJ), the Data Bank of Proteins (PDB), and reference sequences (RefSeq) of NCBI. Once the identity of the identified bacterial species was assigned, the 80 best 16S rDNA sequences were selected and phylogenetic analyses made. Conserved aligned regions (> 90%) were selected in all sequences and phylogenetic analysis was performed in the software MEGA version 6.0. Bacterial abundance data was analyzed and plotted with the ORIGIN software.

### Isolation of Antibiotic-Resistant Bacteria and Characterization of Native Plasmids

Strain isolation was performed by plating concentrated samples and 10X, 100X, and 1000X serial dilutions. The sowing was carried out by plate extension in nutrient agar medium (pluripeptone 5 g, meat extract 3 g, sodium chloride 8 g, and agar 15 g, per liter, at a pH of 7.3) and LB agar medium (casein peptone, yeast extract, and sodium chloride), supplemented with the following antibiotics: ampicillin (25 mg/mL), tylosin (125 µg/mL), oxytetracycline (64 mg/mL), and chloramphenicol (5 µg/mL). The cultures were grown at 37 °C for 16 h. Subsequently, the grown colonies were cultured in 4 mL of liquid LB supplemented with the respective antibiotics. The cultures were incubated at 37 °C for 16 h.

DNA plasmid was extracted by the alkaline lysis method. The electrophoretic profile of the isoforms of these plasmids was performed on 1% agarose gels. The determination of the molecular weight of the plasmids was based on the comparison of the isoforms of the native plasmids with the electrophoretic profile of the supercoiled DNA Ladder (#N0472, NEW ENGLANDS BioLabs). This marker contains 9 supercoiled plasmids, ranging in size from 2 to 10 kb. The 5 kb plasmid has increased intensity and served as a reference to estimate plasmid sizes.

### Morphological Characterization and Resistance or Susceptibility Profile of the Isolated Strains Against Antibiotics

Gram stains were realized to determine the bacterial morphology of the strains and the strains were seeded on MacConkey agar to determine their Lac + and Lac- physiology. The determination of the MIC_90_ was carried out through microdilution in a plate. Sterile, covered, 96-well plastic plates with a U-shaped bottom were used. The culture medium used was Müeller-Hinton broth. 50uL of the culture medium were placed in each well. Then 50 µL of the stock antibiotic solution were placed. The plate was incubated at 37 °C for 16 h. Subsequently, a series of dilutions of the inoculum was made and plated in medium for antibiotic No. 2. It was incubated at 37 °C for 16 h. The following day, the CFU (colony forming units) were counted and multiplied by the dilution obtaining CFU/mL values. Ten consecutive dilutions were worked from a mother solution of each antibiotic. The concentrations of each antibiotic used are described in Table S1.

Two methodologies were used to determine susceptibility or bacterial resistance. Initially, it was determined by the agar diffusion technique-Bauer & Kirby Technique. The bacterial inoculum was adjusted to a standard concentration of 0.5 McFarlane and seeded on the surface of a dry Müeller-Hinton agar plate. After a few minutes, we placed filter paper disc No. 2, with different concentrations of antibiotics (Fig. S1). The plate was incubated at 35 °C in ambient air for a period of 16 h. Each plate was observed in direct light and each inhibition zone was measured using a graduated ruler. The diameters around each disc were measured and their interpretation is based on Table S2.

On the other hand, to determine bacterial susceptibility or resistance, the BD Phoenix system was used. The antibiotics evaluated were Cefriaxone (CRO), Cefepime (FEP), Tetracycline (TE), Piperacillin/tazobactam (TZP), Imipenem (IPM), Gentamicin (GM), Amikacin (AN), Sulbactam/Ampicillin (SAM), Amoxicillin Clavulanic Acid (AMC), Ertapenem (ETP), Meropenem (MEM), Cefotaxime (CTX), Ceftotaxime/Amoxicillin Clavulanic Acid (CTX/CLAV), Ceftazidime (CAZ), Ceftazidime/ Amoxicillin Clavulanic Acid (CAZ/CLAV) and the points the cut-off values used to calculate susceptibility or resistance are those described for Gram-negative bacilli (Table S3).

### Identification of Bacterial Strains for 16S rDNA Sequencing

The gDNA extraction of each strain was carried out with the Plant/Fungi DNA Isolation kit (Norgen Biotek Corp.) following the manufacturer's recommendations. Amplification of the 16S rDNA region was carried out using the 16S-fw5'AGAGTTTGATCCTGGCTCAG3' and 16S-rev 5'CGGGAACGTATTCACCG3' oligonucleotides. The reaction mixture consisted of 2.5 µL of 10X Buffer, 1.5 µL of 50 mM MgCl_2_, 1µL of each primer (forward and reverse) at a concentration of 5 uM, 0.25 µL of 10 mM DNTP, 0.20 µL of Taq polymerase (5U/µL), and 50 ng of gDNA. The reaction mixture was brought to a final volume of 25 µL. The reaction conditions were: 1 cycle of 94 °C for 1 min, followed by 35 cycles of 60 °C for 1 min, 72 °C for 1 min, and 72 °C for 5 min. The amplified fragments were verified and identified by electrophoresis in 1% agarose gels. The sequencing and sequence analysis was the same as reported in the metagenomic analysis section.

### Determination of Relative Expression of ARGs Genes in Strains Isolated

#### Conditions of Induction of the Expression of ARGs

Strains were cultured in 4 mL of liquid LB and incubated at 37 °C for 16 h at 100 rpm. Bacterial cells were adjusted to 1 × 10^9^ cells/mL and inoculated into 25 mL of liquid LB supplemented with antibiotics ranges ampicillin (50 mg/mL and 100 mg/mL), tylosin (150 µg/mL and 200 µg/mL), oxytetracycline (150 mg/mL and 200 mg/mL), and chloramphenicol (25 µg/mL and 50 µg/mL). Cultures were incubated at 37 °C for 24 h, at 100 rpm. Samples were taken at 1, 9, 17, and 24 h.

#### Relative Expression of ARGs

RNA isolation and cDNA synthesis were conducted according to Tamayo-Ordóñez et al. (2016) [22]. The amplification of *act* gene (ampicillin resistance gene), *tetA* (oxytetracycline resistance gene), *cat*1 (chloramphenicol resistance gene), *ermB* (macrolide resistance gene), were determined by real-time PCR (qPCR). The characteristics of the used oligonucleotides are described in Table S4. The melt curve analysis and negative controls for the reference and target genes were always included in the experiments to eliminate gDNA contamination.

The relative expression of each gene was determined by the ∆∆Cq method between the target and reference (16S rDNA) genes. The transcript abundance ratio of target gene to reference gene was determined by the following equation: Relative Expression = (E_ref_)^Ctref^/(E_target_)^Ctarget^, where E_ref_ and E_target_ are the efficiencies of the primers for the reference and target genes, respectively, and Ct _ref_ and Ct _target_ are the mean *C*_T_ value of the reference and target genes, respectively.

### Identification of Genetic Elements in gDNA from Wastewater Samples

The identification of genetic elements such as *sul* (sulfonamide resistance gene) and *qnr* (quinolone resistance genes), *cat*1 (chloramphenicol resistance gene), *aad*A1 (spectinomycin-streptomycin resistance gene), and *sat*-1 (kanamycin resistance gene) were determined by real-time PCR (qPCR). The characteristics of the used oligonucleotides are described in supplementary Table S4.

To determine the copy numbers of the genes of interest (absolute RT-qPCR), we construct calibration curves (Ct vs copy total numbers) to interpolate the Ct values obtained from our samples. The data were analyzed in triplicate and the mean value was plotted and subjected to a Tukey test (α = 0.05). For a detailed description of the methods used to estimate the number of copies is provided in the supplementary file.

## Results

### Presence of Antibiotics, Genetic Elements, and Bacterial Abundance in Wastewater

The physicochemical characterization of the treated and untreated wastewater samples indicated that the BOD was higher in untreated (573 ± 13 mg/L) than in treated wastewater, in which we observed a 95% decrease in BOD (Table 1).

**Table 1:** Physicochemical characterization and determination of antibiotics in treated and untreated wastewater

Parameters analyzed	Untreated wastewater	Treated waste water	Reduced percentage between untreated wastewater and in treated wastewater
Total alkalinity (CaCO3) (mg/L)	706.00 ± 5.00	161.00 ± 3.40	77.19
Total hardness (CaCO3) (mg/L)	338.00 ± 3.00	256.00 ± 4.00	24.26
Chlorides (mg/L)	33.70 ± 2.00	26.40 ± 3.00	22.84
Dissolved oxygen (mg/L)	7.10 ± 5.00	4.70 ± 3.00	33.80
Biochemical Oxygen Demand (mg/L)	573.00 ± 13.00	28.00 ± 4.00	95.11
Ampicillin (µg/mL)	49.74 ± 5.70	25.55 ± 3.02	49.66
Chloramphenicol (µg/mL)	0.60 ± 0.03	0.02 ± 0	96.66
Tylosin (µg/mL)	72.95 ± 2.03	29.96 ± 3.02	58.93
Oxytetracycline (µg/mL)	0.22 ± 0.01	0.08 ± 0.020	63.63

The determination of antibiotics by HPLC allowed us to demonstrate the presence of antibiotics such as ampicillin (49.74 ± 5.70 µg/mL), chloramphenicol (0.60 ± 0.03 µg/mL), tylosin (72.95 ± 2.03 µg/mL), and oxy-tetracycline (0.22 ± 0.01 µg/mL), the concentrations of which decreased between 49 and 99% in treated relative to untreated wastewater (Table 1 and Fig. S2A). Indicating that antibiotic removal techniques could favor eliminating antibiotics in treated water. Residual concentrations of antibiotics present in untreated wastewater could be sufficient to favor the presence of bacteria that have demonstrated antibiotic resistance.

Using qPCR, we identified the presence of genes that confer resistance to sulfonamides (*sul*), quinolones (*qnr*) chloramphenicol (*cat*1), spectinomycin, streptomycin (*aad*A1), and kanamycin *(sat-1*), which were present in higher copy numbers in gDNA samples of untreated than in treated wastewater (Fig. S2B). The number of copies of antibiotic resistance genes present in a sample relates to the abundance of microorganisms that contain these genetic elements, which agrees with the higher BOD values we recorded in the untreated water samples (Table 1). This result indicated that in the untreated water samples, bacteria with antibiotic resistance could be found.

If we compare the percentage decrease in the gene copy number present in the treated wastewater samples, it is observed that the number of copies of *cat*1 and *aad*A1 was 93% less than in untreated wastewater. The detection of chloramphenicol concentrations in treated water samples indicated a reduction of 96.66% relative to untreated water (Table 1), suggesting that the wastewater treatment plant apparently reduces the presence of chloramphenicol, and possible microorganisms with resistance to this antibiotic, by over 90%.

The number of copies of the *sat-1* gene was 83% lower in the samples from treated than from untreated wastewater, while that for the genes conferring resistance to sulfonamides and quinolones decreased 48% and 68%, respectively (Fig. S2B). These reductions in gene copy number we observed in samples of treated wastewater suggest that the inadequate disposal of antibiotics contributes to the presence of emerging pollutants in water.

**Fig. 1:**
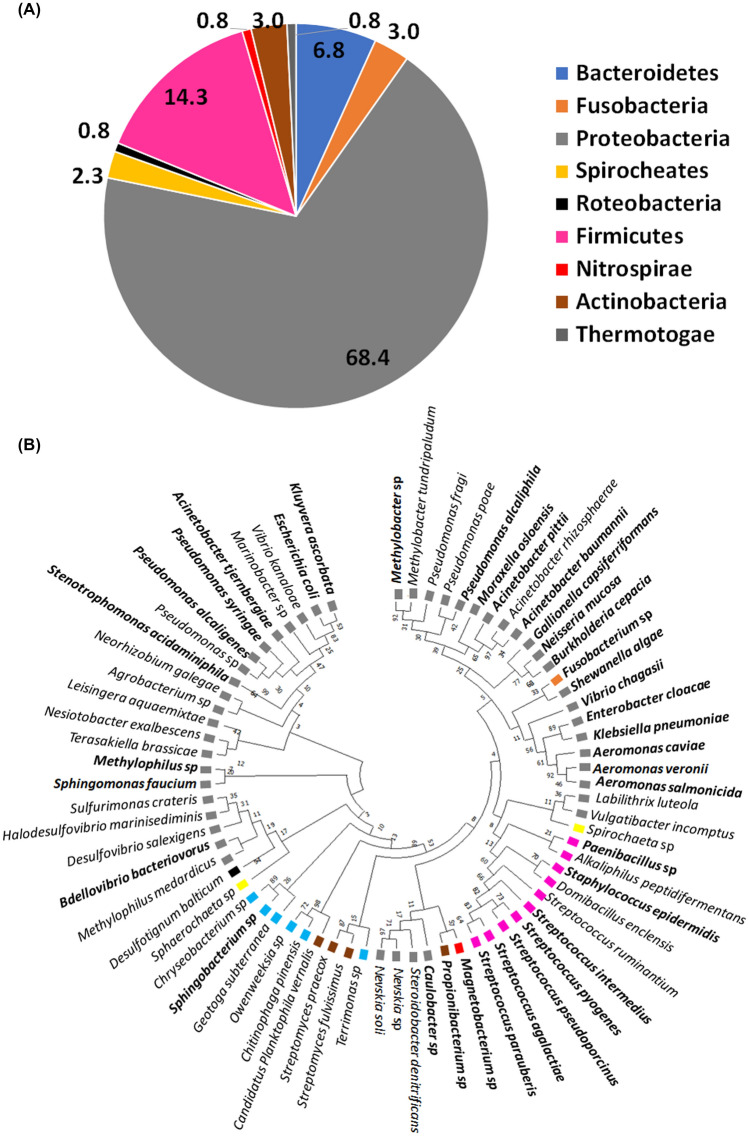
Percentage of bacterial phyla and cluster including species identified in untreated water by metagenomic analysis. untreated water samples. **a** The identification of the bacterial species belonging to each phylum was determined by metagenomics and 16S rDNA sequencing, the identity analysis was performed by BlastN. The results cover a total of 1330 sequences analyzed. the model used was from Minima Evolution. **b** The dendrogram modeling was carried out with the MEGA v.6.0 program, using the method of minimal evolution to boopstrap of 1000 repetitions

The 1330 sequences of 16S rDNA analysis allowed us to identify the abundance of bacteria present in the untreated wastewater samples we analyzed. The percentages of sequence identity were from 99 to 100% (Table S5). We found a total of 58 genera representing nine phyla (Fig. 1a). The most abundant phylum was Proteobacteria (68%), followed by Firmicutes (14.3%), and Bacteroidetes (6.8%). Our analysis identified 83 species in 58 genera representing the nine phyla (Fig. 1a and Table S5). To know how these bacterial species group phylogenetically, we constructed a dendrogram that included 71 bacterial species (Fig. 1b). Of the 46 bacterial species belonging to the phylum Proteobacteria, at least 25 (> 50%) have demonstrated antibiotic resistance (Table S6). We also obtained two distant groups of species belonging to the phylum Proteobacteria. The genera *Methylobacter*, *Pseudomonas*, *Moraxella*, *Acinetobacter*, *Gallionella*, *Neisseria*, *Burkholderia*, *Fusobacterium*, *Shewanella*, *Vibrio*, *Enterobacter, Klebsiella*, and *Aeromonas* proved to be closer to representatives of the phylum Firmicutes. The most phylogenetically distant group of proteobacteria included bacterial species in the genera *Terasakiella*, *Nesiotobacter*, *Leisingera*, *Agrobacterium*, *Neorhizobium*, *Stenotrophomonas*, *Marinobacter*, *Escherichia*, and *Kluyvera* (Fig. 1b). We found species in the genera *Pseudomonas*, *Acinetobacter*, and *Vibrio* in both groups of Proteobacteria, indicating possible genetic variability between these accessions.

Of the 10 accessions belonging to the phylum Firmicutes, the presence of antibiotic-resistant species of *Paenibacillus*, *Staphylococcus*, and *Streptococcus* stands out. Apparently, our metagenomic analysis indicates that untreated wastewater samples might contain at least 36 bacterial species resistant to a wide range of antibiotics (Table S6).

### Isolation of Multidrug-Resistant Strains and Genetic Characterization of Native Plasmids

A total of twenty-two Gram-negative strains were identified, with morphologies of bacilli in short chains and in pairs and Bacilli that grow in the form of fibers (Fig. S3 and Table S7). The phenotype present in most of the isolates was Lac + , except for strain-22, which proved to be Lac-. Plate growth demonstrated phenotypic morphologies of rough, smooth, and mucoid colonies (Fig. S4 and Table S7). The respective MIC_90_ of these strains against ampicillin, chloramphenicol, tylosin, and oxytetracycline were 100 mg/mL, 50 µg/mL, 200 µg/mL, and 200 mg/mL, respectively. Values higher than determined in the residual water by HPLC suggest that these bacteria inhabit a suitable environment.

16S rDNA sequencing indicated that most isolates belonged to the *E. coli* (identity ≥ 90%). Strains-15, -16, -18, and -20 indicated that they belong to the *Bacteroides fragilis* (identity ≥ 95%), and strain-22 indicated to belong to *Salmonella typhi* (identity ≥ 98%) (Table S8).

**Fig. 2:**
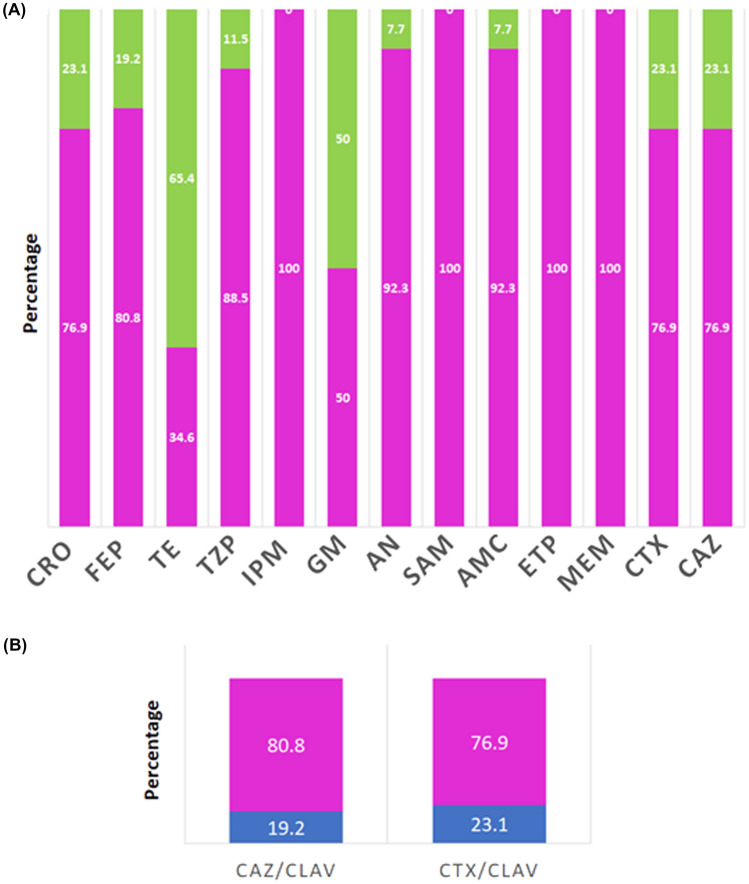
Profile of antimicrobial resistance against pure and combined antibiotics. **a** For the analysis, the strains were exposed to various antibiotics: Cefriaxone (CRO), Cefepime (FEP), Tetracycline (TE), Piperacillin/tazobactam (TZP), Imipenem (IPM), Gentamicin (GM), Amikacin (AN), Sulbactam/ Ampicillin (SAM), Amoxicillin Clavulanic Acid (AMC), Ertapenem (ETP), Meropenem (MEM), Cefotaxime (CTX), Ceftotaxime/ Amoxicillin Clavulanic Acid (CTX/CLAV), Ceftazidime (CAZ), Ceftazidime/ Amoxicillin Clavulanic Acid (CAZ /KEY). The percentage of sensitive bacteria is indicated in a pink bar. The percentage of resistant bacteria is indicated in a green bar. **b** Resistance or sensitivity analysis against Ceftotaxime/amoxicillin clavulanic acid (CTX/CLAV), and Ceftazidime/Amoxicillin clavulanic acid (CAZ/CLAV). The blue bar indicates the percentage of resistant strains, and the pink bar indicates the percentage of sensitive strains

**Fig. 3:**
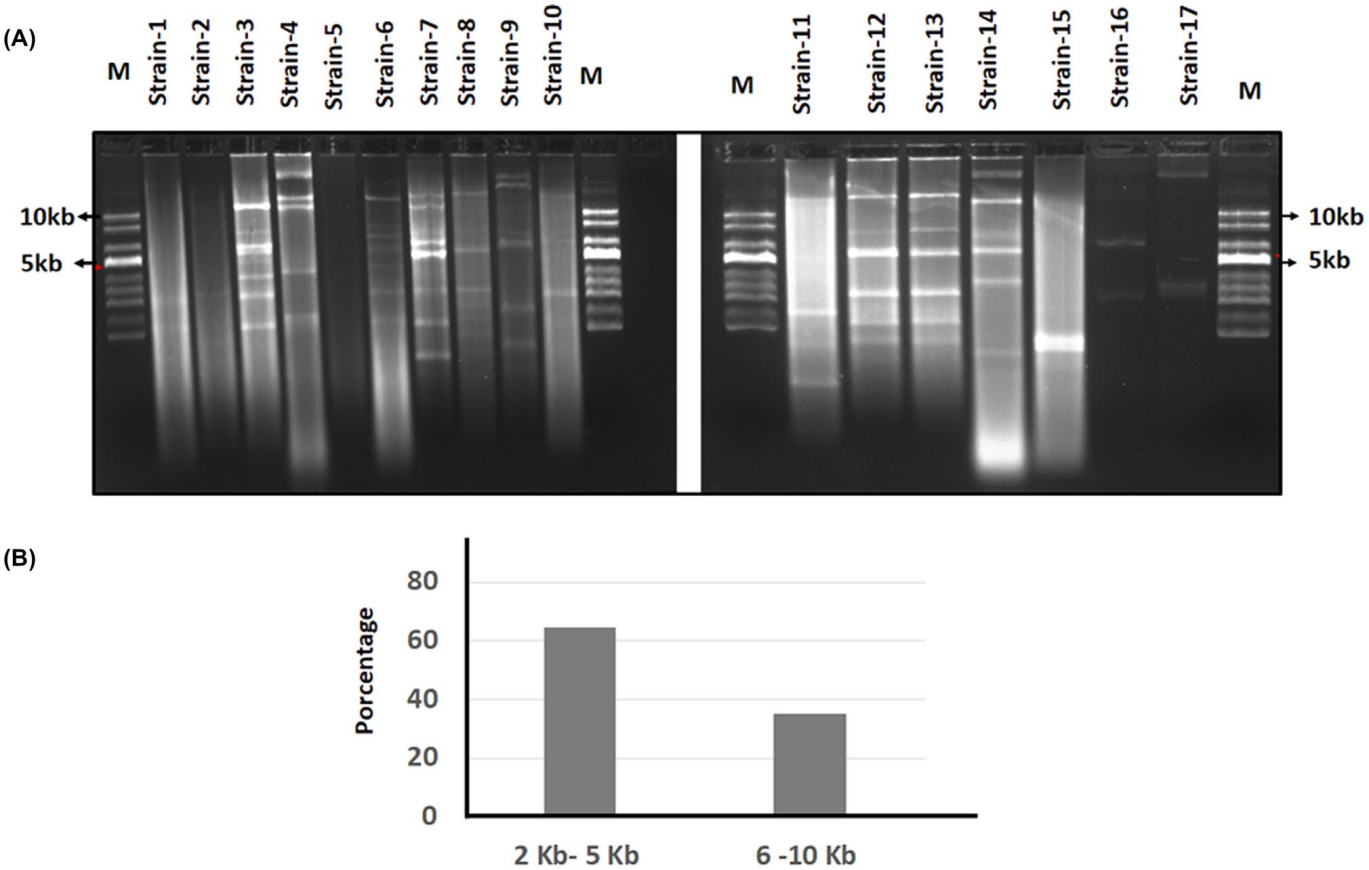
Electrophoretic profile of plasmids extracted from isolated strains. **a** Electrophoresis was performed on 0.8% agarose gels at 75 V for 16 h. As a comparison marker, the Supercoiled DNA Ladder was used. **b** Estimation of the size of the plasmid, comparing the isoform profile with the isoform profile contained in the supercoiled DNA Ladder

The antimicrobial resistance or susceptibility profile against 15 compounds indicated that the strains are multidrug-resistant (Fig. 2a). 100% of the strains indicated sensitivity against ETP, MEM, and IPM. 92% of the strains show sensitivity to AN. 64% of the strains showed resistance to TE and 50% to GM. Furthermore, 10 to 25% of the strains showed resistance against CRO, FEP, TZP, CTX, and CAZ. In the case of AN and AMC it was indicated that 7.7% of the strains are resistant to these compounds (Fig. 3a). Evaluating antibiotics in combination, it was shown that more than 19% of the strains are resistant to CTX/CLAV and CAZ/CLAV (Fig. 2b).

In the least 14 strains, it was possible to isolate plasmids (Fig. 3). It is estimated that at least 64% of these 14 strains harbor small plasmids with sizes between 2 and 5 Kb and the other 35% of strains harbor plasmids with sizes between 6 and 10 Kb (Fig. 3a and b). In ten isolated strains, bands larger than 10 Kb were observed that could correspond to large plasmids or megaplasmids (Fig. 3a). Future studies of genetic characterization of plasmids in bacterial isolated are needed to know the incompatibility types, plasmid sizes, transconjugant frequencies, and antibiotic resistance genes they contain each plasmid.

### Differential Expression of ARGs, in Bacterial Isolates Under Stress with Antibiotics

Various studies point to the relationship between environmental factors and the expression of ARGs [23–25]. In this research, we decided to find out the transcriptional behavior of the *cat*1, *tet*A, *Erm*B, and *Amp*C genes under pressure of chloramphenicol, oxytetracycline, tylosin, and ampicillin, respectively.

#### Relative Expression of Cat1 Gene

**Fig. 4:**
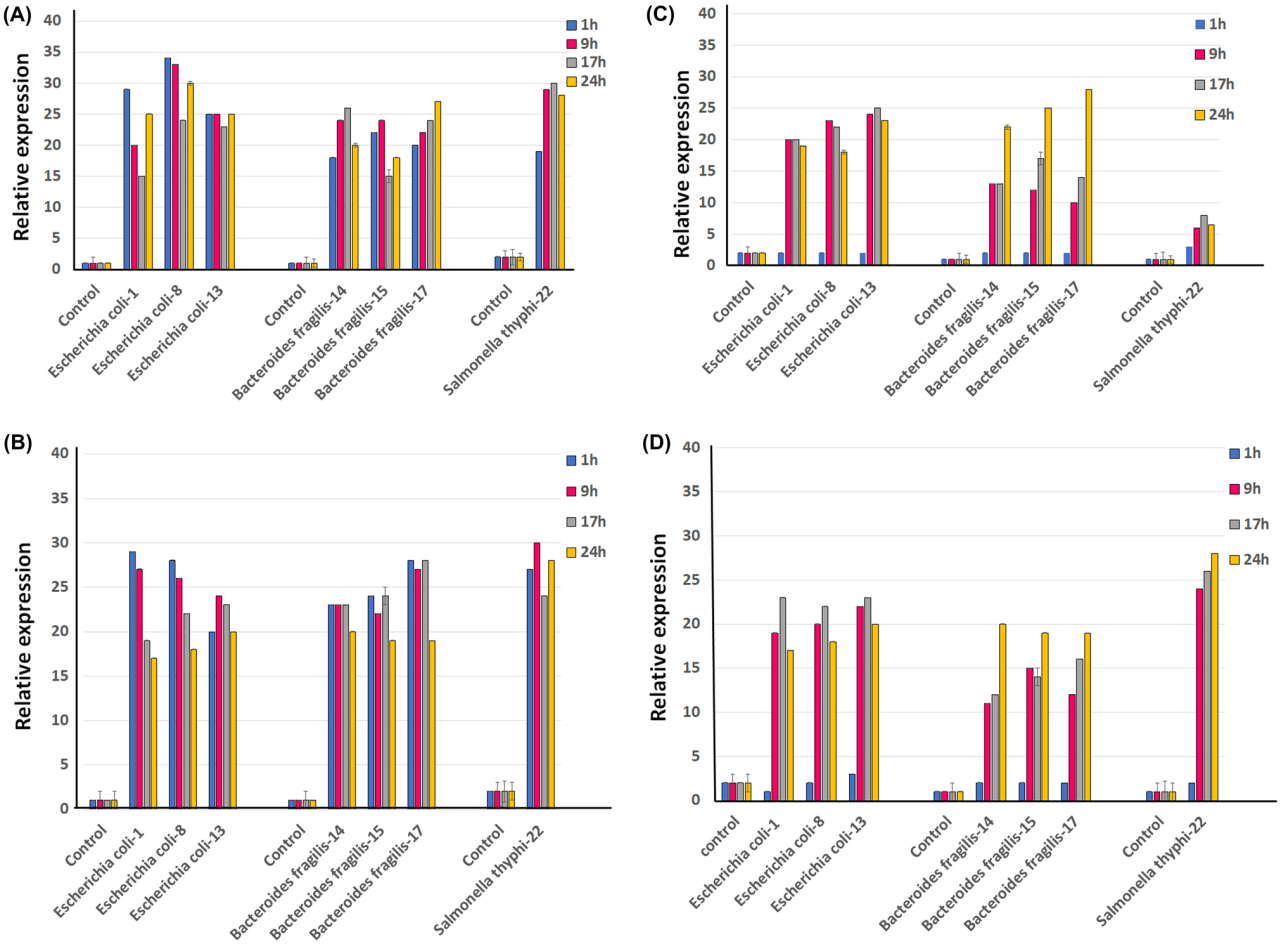
Relative expression of the *cat* and *tetA* gene, under culture conditions supplemented with chloramphenicol and oxytetracycline. Samples were taken at 1, 9, 17, and 24 h after exposure to the antibiotic. As a negative control, the strains cultivated without the presence of the antibiotic were included. **a** Cloramphenicol at 25 µg/mL, **b** Cloramphenicol at 50 µg/mL, **c** Oxytetracycline at 150 mg/mL, and **d** Oxytetracycline at 200 mg/mL

The expression of the *cat*1 gene is translated into the enzyme CAT (Chloramphenicol acetyl transferase [26]. This enzyme acetylates the molecule of the antibiotic chloramphenicol, which causes it to lose its effect. Analysis of the relative expression of the *cat1* gene, in the species *Escherichia coli*-1 *Escherichia coli*-8, *Escherichia coli*-13, *Bacteroides fragillis*-14, *Bacteroides fragillis*-15, *Bacteroides fragillis*-17, *Salmonella thyphi*-22¸ was carried out by analyzing two concentrations of chloramphenicol (25 µg/mL and 50 µg/mL), for 1, 9, 17, and 24 h under exposure to the antibiotic (Fig. 4). *E*. *coli* presents the highest relative expression of the *cat1* gene at 9 h in exposure with the antibiotic (25 µg/mL) (Fig. 4a). *B. fragilis* strains shows to express 10 times less expression of this gene compared to *E. coli* strains, demonstrating its maximum expression at 9 h (*Bacteroides fragillis*-14, *Bacteroides fragillis*-15) and 24 h (*Bacteroides fragillis*-17). *Salmonella thyphi*-22, demonstrated to express 5 times less expression of the *cat1* gene compared to *E. coli* isolates, reaching its maximum expression at 17 h (Fig. 4a).

The behavior of expression in *E. coli* strains, when the concentration of chloramphenicol (50 µg/mL) was increased, showed that after 24 h of exposure to the antibiotic (Fig. 4b), there was an eightfold reduction compared to treatments with chloramphenicol at 25 µg/mL. In the *B. fragilis* isolates, the increase in the concentration of chloramphenicol did not cause a significant change in the expression of the *cat1* gene. In *Salmonella thyphi*-22, a sixfold increase in expression was demonstrated at 9 h compared to low concentrations of chloramphenicol (25 µg/mL).

#### Relative Expression of tetA Gene

The *tetA* gene confers resistance to oxytetracycline [27], this gene encodes specific efflux pumps for this antibiotic. The relative expression of this gene under a concentration of 150 mg/mL of oxytetracycline had a similar behavior among the *E. coli* isolates, showing lower expression at 1 h of exposure to the antibiotic and raising its expression eighteen times more at 9 h under the presence of oxytetracycline, maintaining constant expression during the 24 h evaluated. In the case of *B. fragillis*, it showed behaviors similar to isolated *E. coli*, indicating its lower expression of *tetA* at one hour and an increase at 9 and 17 h. *B. Fragilis* demonstrated a tenfold increase in its expression at 24 h compared to 17 h. *S. thyphi*-22 had a lower expression (20 times less) in relation to the higher levels of expression of *E. coli* and *B. Fragilis* (Fig. 4c).

The relative expression of the *tetA* gene with a medium supplemented with 200 mg/mL oxytetracycline (Fig. 4d), was similar to that of the culture medium supplemented with 150 mg/mL oxytetracycline, one of the most relevant differences in both concentrations was that the relative expression of the gene in *S. thyphi*-22 was higher than 200 mg/mL, having a higher expression at 24 and 9 h, reaching relative expressions 20 times more compared to lower concentrations of oxytetracycline (150 mg/mL) (Fig. 4c).

#### Relative Expression of Act Gene

Β-lactamase activity is related to antibiotic resistance, mostly in gram-negative bacteria. In *E. coli*, at least 10 β-lactamase genes have been reported, among them the *act* gene belongs to AmpC β-lactamases (ABL) [28]. The strains analyzed in this research mostly demonstrated resistance to beta-lactams (Fig. 2), therefore in this research we included the transcriptional analysis of the *act* gene, in the presence of ampicillin at two concentrations (50 mg/mL and 100 mg/mL), during 24 h of culture.

**Fig. 5:**
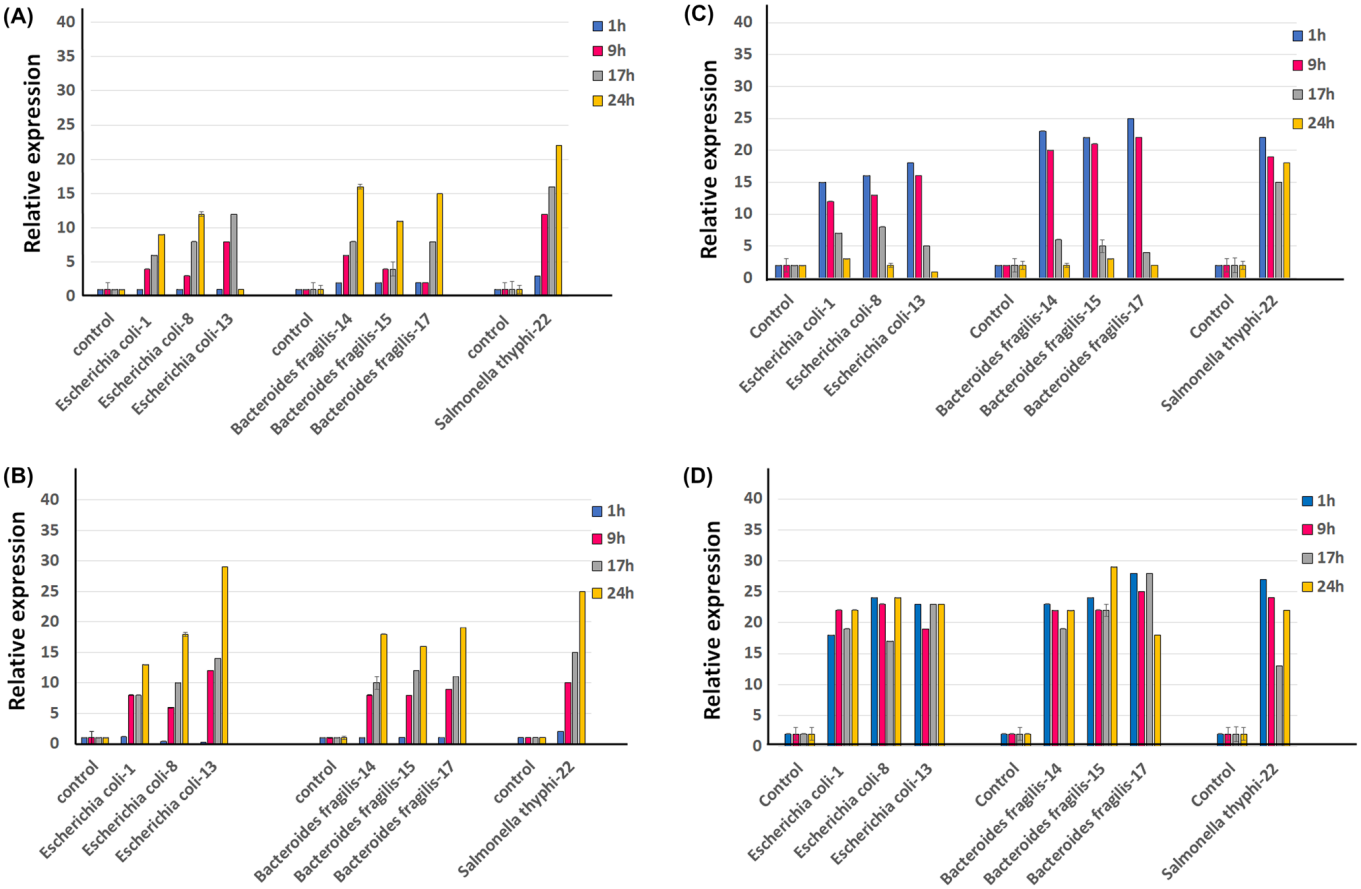
Relative expression of the *act* and *ermB* gene, under culture conditions supplemented with ampicillin and tylosin. Samples were taken at 1, 9, 17, and 24 h after exposure to the antibiotic. As a negative control, the strains cultivated without the presence of the antibiotic were included. **a** Ampicillin at 50 mg/mL, **b** Ampicillin at 100 mg/mL, **c** tylosin at 100 µg/mL, and **d** tylosin at 200 µg/mL

The relative expression in representative strains of the *Escherichia*, *Bacteroides,* and *Salmonella* genera showed that the *act* gene increases its expression during 24 h in the presence of ampicillin in both concentrations analyzed (Fig. 5a). *S. thyphi*-22 indicated the highest expression levels at 24 h compared to *E. coli* and *B. fragilis* isolates.

The expression level of the *act* gene does not show significant differences that indicate a considerable increase between treatments with ampicillin (50 mg/mL or 100 mg/mL), in the species evaluated, except for *E. coli*-13, which demonstrated to increase its relative expression of the *act* gene 28 times more in comparison when this strain was evaluated with treatment with lower concentrations of ampicillin (50 mg/mL) (Fig. 5b).

#### Relative Expression of ermB Gene

Macrolide antibiotics bind to the ribosome and then activate the expression of the antibiotic resistance genes *ermC* or *ermB* [29, 30]. We evaluated the expression of the *ermB* gene in bacterial cultures with concentrations of 100 µg/mL and 200 µg/mL of tylosin, for 24 h (Fig. 5c and d). Similar transcriptional expression of the *ermB* gene was observed in *E. coli* and *B. fragilis*, exposed to both tylosin concentrations (100 µg/mL and 200 µg/mL). In cultures with tylosin concentrations of 100 µg/mL both isolates show high expression values at 1 h of culture, later this expression decreases during the 24 consecutive hours, a tenfold decrease in the relative expression of the *erm*B gene is observed in *E. coli* and 20 times less in *B. fragilis*. In addition, *B. Fragilis* expressed 5 times more of the *ermB* gene at 1 h of culture than *E. coli*. On the other hand, *S. thyphi*-22 showed a constant expression during the 24 h of culture (Fig. 5c).

In cultures with concentrations of 200 µg/mL of tylosin, it was observed that the three species analyzed showed constant expression values during the 24 h evaluated (Fig. 5d). Expression levels were similar to those obtained when isolates were grown at (100 µg/mL).

## Discussion

Analyzing the occurrence of the antibiotics detected, such as ampicillin (49.74 ± 5.70 µg/mL), chloramphenicol (0.60 ± 0.03 µg/mL), tylosin (72.95 ± 2.03 µg/mL), and oxytetracycline (0.22 ± 0.01 µg/mL) with the identification by metagenomic of bacterial species in wastewater samples, we can highlight resistance to ampicillin with MIC_90_ within a range from 56 to 256 µg/mL in *Enterobacter cloacae*, *E. coli*, *Pseudomonas* sp, *Bacillus coreaensis*, *Moraxella osloensis*, *Methylobacter* sp., *Paenibacillus* sp., *Aeromonas* sp., *Caulobacter* sp., and *Vibrio* sp. (Table S6), have been reported.

Among the species we found, some have been reported to be resistant to chloramphenicol with MIC_90_ in ranges from 256 µg/mL to 4 mg/mL like *Pseudomonas* sp., *Bacillus coreansis*, *Methylobacter* sp., *Streptococcus pyogenes*, *E. coli*, and *Aeromonas salmonicida*. Resistance to tylosin has been reported for *E. coli* with MIC_90_ of 15 μg/L. Resistance to oxy-tetracycline is known to occur in *Bacillus coreaensis* (80%), *Sphingobacterium* sp. (MIC of 128 mg/mL), and *Magnetobacterium* sp. (Table S6).

The concentration of ampicillin (49.74 ± 5.70 µg/mL) we observed in wastewater (Table 1) is within the MIC_90_ value reported for some bacterial species (Table S6). Depending on the type of bacteria analyzed, it can resist different concentrations of antibiotics. According to Li et al. (2019) [18] some bacterial strains of *E. coli* exposed to antibiotics for prolonged periods tend to accumulate SNPs, which allows them to increase their range of antibiotic resistance.

Furthermore, the adaptation of bacteria to high concentrations of antibiotics in their habitat will be favored in bacterial strains with genetic elements such as plasmids and integrons that allow them to survive in these conditions. A recent study where it was predicted how gene-by-gene by-environment (G × G × E) interactions affect the evolution of *E. coli* against pressure at various concentrations of antibiotics, it was shown that G × G interactions and rugged fitness landscapes in the absence of antibiotics, but as antibiotic concentration increased, the fitness effects of ABR genotypes quickly overshadowed those of gene knock-outs, and the landscapes became smoother [31], suggesting that the evolution of resistant bacteria to antibiotics is facilitated by extreme habitats (with the presence of antibiotics) [32].

According to our results and what has been described in the literature, at least 14 species identified by metagenomic analysis (*Enterobacter cloacae*, *E. coli*, *Pseudomonas* sp,, *Bacillus coreaensis, Moraxella osloensis*, *Methylobacter* sp., *Paenibacillus* sp., *Aeromonas* sp., *Caulobacter* sp., *Vibrio sp., Streptococcus pyogenes, Aeromonas salmonicida, Sphingobacterium* sp., and *Magnetobacterium* sp.) have shown resistance to the antibiotics we detected by HPLC (chloramphenicol, oxy-tetracycline, tylosin, and ampicillin), suggesting that the presence of these antibiotics in wastewater may favor the adaptation of these bacterial genera and favor processes such as plasmid conjugation and horizontal gene transfer between the bacterial community.

On the other hand, the identification of genetic elements present in wastewater samples is important to highlight that among the bacteria we identified by metagenomic analysis, the occurrence of genes that confer resistance to sulfonamides such as *sul*1, *sul*2, and *sul*3, were identified in *Fusobacterium* sp. by Xiong et al. (2014) [33], *Acinetobacter baumannii* by Girija et al. (2019) [34], *Aeromonas* sp. by Piotrowska and Popowska (2014) [35], and *Klebsiella pneumoniae* by Shahid et al. (2012) [36]. Genes conferring resistance to quinolone (*qnr*) were reported by Saga et al. (2005) [37] to be present in *Vibrio parahaemolyticus*, Salah et al. (2019) [38] found them in *E. coli*, Touati et al. (2008) [39] in *Acinetobacter baumannii*, and Sivaraman et al. (2020) [40] in *Klebsiella pneumoniae*. The *cat* gene that confers resistance to chloramphenicol was identified by Piotrowska and Popowska (2014) [35] in *Aeromonas salmonicida*, *A. veronii*, and *A. caviae*. The gene conferring resistance to spectinomycin and streptomycin (*aad*A1) was found to be present in *Acinetobacter baumannii* by Huang et al. (2015) [41], *Aeromonas veronii* and *Aeromonas caviae* by Piotrowska and Popowska, 2014 [35], and *Klebsiella pneumoniae* by Sivaraman et al. (2020) [40]. In the case of the *sat-1* gene associated with resistance to kanamycin, it was identified in *E. coli* by Peerayeh et al. (2019) [42]. The occurrence of these bacteria in our samples of wastewater correlates to the detection of these genes conferring antibiotics resistance (Fig. 1B), suggesting that early detection of genetic elements (ARGs) in water samples could be used as a preliminary method that indicates the possible presence of bacterial genera with resistance to a variety of antibiotics [43].

Most of our isolates show a high identity with *Escherichia coli*, bacteria that is known to resist a wide range of antibiotics, as Williams et al. (2019) [44] demonstrated for chloramphenicol (MIC range of 64–512 mg/L), and as reported for ampicillin (MIC > 256 µg/mL) and tetracycline (256 µg/mL).

In addition to resistance against tylosin, ampicillin, chloramphenicol, and oxytetracycline, the strains also proved to be multidrug resistant against eleven of the compounds evaluated (Fig. 2). This suggests that the bacterial isolates can resist to β-lactams, aminoglycosides, tetracyclines, sulfonamides, and quinolones. In bacteria, the genes responsible for resistance against macrolide antibiotics have been reported in small plasmids (< 15 kb in size) [45]. In *E. coli*, the genes responsible for resistance to ampicillin, chloramphenicol, and tetracycline have been shown to be on large conjugative plasmids, pIS46, pIS66, and pIS102 [46]. In highly pathogenic bacteria such as *Salmonella* and *Pseudomonas* it has been described that resistance genes to a wide range of antibiotics are in megaplasmids (280 Kb and 423 Kb) [47]. This research suggests that the plasmids isolated from each strain could contain the identified ARGs, which could play an important role in the resistance of the isolates against the antibiotics analyzed.

Unfortunately, there are few studies that evaluate the transcriptional behavior of ARGs against antibiotics. Most of the studies carried out consist of identifying the presence of these ARGs or evaluating them globally and not punctually [48]. This makes our interpretation of results difficult, however we can highlight that the results obtained from the relative expression of the *act*, *tetA*, *cat1,* and *ermB* gene, showed that the expression behavior depends on the type of antibiotic to which the strain is exposed, the mechanism of action of each antibiotic and the genetic background of each species analyzed to deal with this stress condition against antibiotic.

The resistance of bacteria against chloramphenicol and oxytetracycline is related to the action of efflux pumps [49, 50]. At least three gene and their variants (*cat*-1, -2, -3, *flo*R, and *cml*A) responsible for resistance against chloramphenicol and for resistance to oxytetracycline have been identified. More than 40 genes encoding tetracycline resistance (tet-genes) have been characterized and they are divided into 11 classes, with a majority of classes (60%) encoding membrane-associated efflux proteins [51]. In *Burkholderia ubonensis tetA* and *tetR* were shown to be significantly induced 16–22 times when this bacterium was exposed to tetracycline and doxycycline [51]. Similar patterns of expression of the *tetA* and *tetR* genes were reported in *E. coli*, when cultured against [52]. Our data indicate similar patterns of *tetA* expression at 9 h vulture, showing a 10–20-fold increase in expression depending on the strain studied and compared to the control (Fig. 4). It is important to point out that the genes related to bacterial resistance through efflux proteins (*cat* and *tet*) showed a higher level of expression in comparison to genes related to other resistance mechanisms (*act* and *erm*) (Figs. 4, 5).

For this part, the β-lactam antibiotics; their mode of action interferes with the synthesis of the cell wall during cell replication. Bacteria have shown the ability to tolerate ampicillin ranges from 56 to 256 µg/mL (Table S6), and to date it has been shown that they can accumulate SNPs, and present plasmids and transposons that facilitate the evolution of bacteria for the formation of new genes and/or gene variants [18]. In *E. coli* at least 10 β-lactamase genes have been reported, suggesting that a strong genetic background allows it to cope successfully with β-lactam antibiotics.

Tylosin belongs to Macrolide antibiotics binding to the ribosome and then activate the expression of the antibiotic resistance genes *ermC* or *ermB*. A study carried out on *Mycobacterium abscessus* showed that minimal concentrations of macrolides allowed better expression of the *erm* gene [53]. Another study by Yao et al. (2019) [54] indicated that the expression of *erm*A, *erm*B, and *erm*C was evaluated in strains with different phenotypes (iMLSB and cMLSB) a differential expression. This suggests that between different isolates, it is possible that the behavior in the regulation of genes related to resistance to antibiotics is differential, for which the importance of carrying out these specific studies is highlighted, which allows us to know how each species or bacterial isolate behaves.

In literature, the determination of antibiotic susceptibility or resistance in isolated or cultured bacteria against tylosin has not been extensively explored. This research showed that our isolates present MIC_90_ of 200 µg/mL, and the relative expression of the *erm* gene indicated that the activity of its encoded protein is possibly involved in resistance. Future studies that allow us to know what the expression of the *ermA*, *ermB*, *ermC* genes is like against this tylosin, would allow us to innovate a biotechnological strategy that allows us to deal with bacterial infections using this antibiotic, minimizing the negative consequences for health.

Finally, we can emphasize that even though we evaluated the expression of genes related to resistance to antibiotics most commonly used in veterinary medicine, we also demonstrated that the isolates could resist new generation antibiotics (third and fourth generation) that are widely used to combat bacterial infections in the medical area (Fig. 2).

Future studies of genetic characterization of plasmids in bacterial isolated are needed to know the incompatibility types, transconjugant frequencies, antibiotic resistance genes they contain, and if plasmid-mediated horizontal gene transfer between bacteria is a significant source of antibiotic resistance in the bacteria present in wastewater treatment plant. The information generated will allow us to build an innovative strategy (conjugative vector) that can facilitate the selection of resistance cassettes in bacteria that inhabit bodies of water.

## Conclusion

In this Project, the incidence of antibiotic residues in wastewater and the presence of bacteria resistant to a variety of antibiotics (including the antibiotics detected in WWTP) were demonstrated. ARGs and plasmids that allow them to resist a wide variety of antibiotics were identified in the isolates. The human population is at risk of contact from this wastewater and the bacteria present in it. Many of the genera identified by metagenomic analyzes have been reported to cause disease, so this is already a public health risk. Investigations such as the one supported in this article are important to know the impact that the incidence of antibiotic-resistant bacteria in human contact waters can have and how this can be a public health risk.

### Supplementary Information

Below is the link to the electronic supplementary material.

Supplementary file1 (DOCX 1454 kb)

## Data Availability

All data are available with corresponding author.
