# Supplementary material for: Regulation Transcriptional of Antibiotic Resistance Genes (ARGs) in Bacteria Isolated from WWTP
Source: Curr Microbiol. 2023 Sep 6;80(10):338. doi: 10.1007/s00284-023-03449-z (PMC10482803; doi:10.1007/s00284-023-03449-z)
Supplement: Supplementary file 1 — Supplementary file1 (DOCX 1454 kb) [file 284_2023_3449_MOESM1_ESM.docx]

**Data supplementary**

| **Table S1.** Concentrations of antibiotics used in the plate microdilution technique. | | | | | | | | | |  |  |
| --- | --- | --- | --- | --- | --- | --- | --- | --- | --- | --- | --- |
|  | 1 | 2 | 3 | 4 | 5 | 6 | 7 | 8 | 9 | 10 | 11 |
| Ampicillin | Negative control | 1 µg/mL | 10µg/mL | 100µg/mL | 1mg/mL | 10mg/mL | 100mg/mL | 1mg/mL | 120mg/mL | 150mg/mL | 200mg/mL |
| Oxytetracycline | Negative control | 5 µg/mL | 50µg/mL | 500µg/mL | 5mg/mL | 50mg/mL | 500mg/mL | 5mg/mL | 520mg/mL | 550mg/mL | 600mg/mL |
| Chloramphenicol | Negative control | 4 µg/mL | 50µg/mL | 500µg/mL | 5mg/mL | 50mg/mL | 500mg/mL | 5mg/mL | 520mg/mL | 550mg/mL | 600mg/mL |
| Tylosin | Negative control | 1µg/mL | 10µg/mL | 100µg/mL | 1mg/mL | 10mg/mL | 100mg/mL | 1mg/mL | 120mg/mL | 150mg/mL | 200mg/mL |
| *The evaluated concentrations of each antibiotic were selected from the literature where the ranges of resistance to these antibiotics by different bacterial genera have been reported. | | | | | | | | | | | |


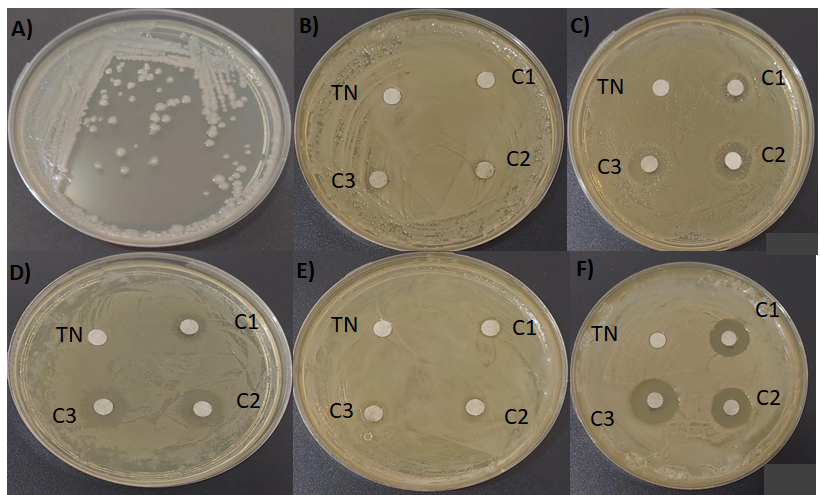


**Figure S1.** Schematic representation of the Bauer & Kirby agar diffusion technique. For the analysis of each group of strains, the *E. coli* strain DH10B ATCC was used as a control (non-resistant). The figure illustrates the results obtained in a representative strain of each group when analyzed by this method, under concentrations already described in the materials and methods section. A) *E. coli* DH10B ATCC, B) *E. coli* DH10B ATCC under ampicillin, C) *E. coli* DH10B ATCC+pGEM under ampicillin, D) strain under chloramphenicol, E) strain under oxytetracycline, F) strain under tylosin. Sections A, B and C were analyzed the following ampicillin concentrations: TN = without antibiotic, C1: 1µg/mL, C2: 10µg/mL, and C3; 100µg/mL. Section D were analyzed the following chloramphenicol concentrations: TN = without antibiotic, C1: 4µg/mL, C2: 50µg/mL, and C3; 500µg/mL. Section E were analyzed the following oxytetracycline concentrations: TN = without antibiotic, C1: 5µg/mL, C2: 50µg/mL, and C3; 500µg/mL. Section F were analyzed the following tylosin concentrations: TN = without antibiotic, C1: 1µg/mL, C2: 10µg/mL, and C3; 100µg/mL.

| **Table S2.** Standard inhibition halo patterns, cut-off points equivalent to MIC for enterobacteriaceae and inhibition halo diameter for the E. coli ATCC25922 strain used as quality control | | | | | | |
| --- | --- | --- | --- | --- | --- | --- |
| Antimicrobial | Inhibition halo diameter (mm) | | | Cut-off Point Equivalent to MIC (ug/mL) | | E. coli ATCC 25922  intervalo |
|  | resistant | intermediate | sensitive | resistant | sensitive |  |
| Ampicillin | ≤ 13 | 14-10 | ≥ 17 | ≥32 | ≤ 8 | 16-22 |
| Tetracycline | ≤ 14 | 15-18 | ≥ 19 | ≥16 | ≤ 4 | 18-25 |
| Chloramphenicol | ≤ 14 | 13-17 | ≥ 18 | ≥32 | ≤ 8 | 21-27 |
| Erythromycin (macrólido)* | ≤ 13 | 14-22 | ≥ 23 | ≥8 | ≤ 8 | 22-30* |
| *The values of Erythromycin a macrolide were used as a reference to compare the halos of tylosin inhibition. These data are those reported in enterococcus. | | | | | | |

| **Table S3. Antimicrobials present in the NMIC 406 panel (Gram negative bacilli) for the automated equipment BD** Phoenix. | | | |
| --- | --- | --- | --- |
| Antimicrobial | MIC | Antimicrobial | MIC |
| Amikacin | ≤ 8 | Ertapenem | ≤ 0.25 |
| Ampicillin | ≤4 | Fosfomycin c/G6P | ≤16 |
| Ampicillin / sulbactam | ≤4/2 | Gentamicin | ≤ 2 |
| Cefazolin | ≤2 | Imipenem | ≤ 0.25 |
| Cefepime | ≤1 | Levofloxacin | ≤ 1 |
| Cefoxitin | ≤4 | Meropenem | ≤ 0.5 |
| ceftazidime | ≤1 | piperacillin/Tazobactam | ≤4/4 |
| Ceftriaxone | ≤1 | Tigecycline  Trimethoprim/Sulfamethoxazole | >2/38 |
| Ciprofoxacin | ≤0.125 |  |  |
| Colistin | ≤0.125 |  |  |

| **Table S4. Oligonucleotides used in this research work** | | | | | |
| --- | --- | --- | --- | --- | --- |
| Target | Amplicon size bp | Name primer sets | Sequence (5´ to 3´) | T_a_  (°C) | Reference |
|  |  |  |  |  |  |
| *sul1* | 293 | SulF | GCGCTCAAGGCAGATGGCATT | 53 | [1] |
|  |  | SulR | GCGTTTGATACCGGCACCCGT |  |  |
|  |  | qnrAF | ATTTCTCACGCCAGGATTTG |  |  |
| *qnrA* | 140 | qnrAR | GCAGATCGGCATAGCTGAAG | 51 | [2] |
|  |  | Cat1R | CGAACTGGCAAAATAACGCA | 57 | In this study* |
| *cat1* | 199 | Cat1F | TCAACAAGTAACACTCCGCT |  |  |
| *aadA1* | 150 | aadA1F | GCGAGCTTTGATCAACGACC | 59 | [3] |
|  |  | aadA1R | ATGTCATTGCGCTGCCATTC |  |  |
| *tetA* | 200 | tetAF | CGGCAATCATTCCGAGCATG | 60 | [3] |
|  |  |  | ATTCTGCATTCACTCGCCCA |  |  |
| *ermB* | 170 | ermBF | TCACCGAACACTAGGGTTGC | 59 | [3] |
|  |  | ermBR | CTGTGGTATGGCGGGTAAGT |  |  |
| *sat1* | 350 | Sat1F | TATGAGAAAAACTCATCGAGCATC | 59 | In this study* |
|  |  | Sat1R | GAATTCAGCCATATTCAACGGGAA |  |  |
| *act1* | 300 | Act1R | TCGGTAAAG CCGATGTTG CGG | 60 |  |
|  |  | Act1F | CTT CCA CTG CGG CTG CCA GTT |  | [4] |

*The primers designed in this study were obtained from alignments and analysis of resistance cassettes contained in bacterial plasmids that are released in the NCBI database.

[1] Arabi, H., Pakzad, I., Nasrollahi, A., Hosainzadegan, H., Jalilian, F. A., Taherikalani, M., ... & Sefidan, A. M. (2015). Sulfonamide resistance genes (sul) M in extended spectrum beta lactamase (ESBL) and non-ESBL producing Escherichia coli isolated from Iranian hospitals. Jundishapur Journal of Microbiology, 8(7).

[2] Marti, E., & Balcázar, J. L. (2013). Real-time PCR assays for quantification of qnr genes in environmental water samples and chicken feces. *Applied and Environmental Microbiology*, *79*(5), 1743-1745.

[3] Vikram, A., Rovira, P., Agga, G. E., Arthur, T. M., Bosilevac, J. M., Wheeler, T. L., ... & Schmidt, J. W. (2017). Impact of “raised without antibiotics” beef cattle production practices on occurrences of antimicrobial resistance. *Applied and Environmental Microbiology*, *83*(22), e01682-17.

[4] Singh, T., Singh, P. K., Das, S., Wani, S., Jawed, A., & Dar, S. A. (2019). Transcriptome analysis of beta-lactamase genes in diarrheagenic Escherichia coli. Scientific reports, 9(1), 3626.


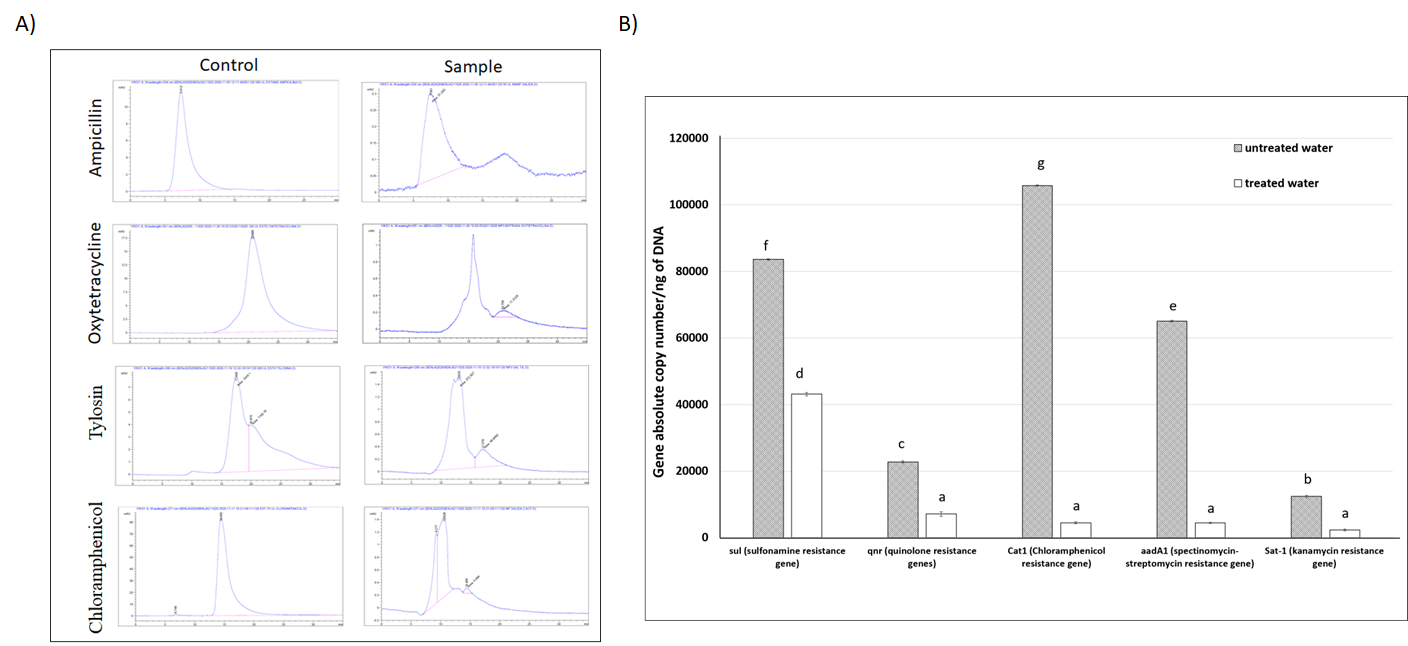


**Figure S2. Antibiotic detection curves and determination of the number of copies of genetic elements in wastewater.** A). On the left side, the standard curves of the used standards of the antibiotic’s ampicillin, oxytetracycline, tylosin and chloramphenicol are shown. On the right side a detection curve of the analyzed samples is illustrated. B. From the respective clones of each gene of interest, calibration curves were constructed that were used to interpolate the Ct values of the samples from treated and untreated water.

| **Table S5. Sequence identity results obtained from metagenomic analysis from untreated water from the Saltillo treatment plant. These sequences were included in the bioinformatic analyzes** | | | | | | | | | | |
| --- | --- | --- | --- | --- | --- | --- | --- | --- | --- | --- |
| **Sequence** | **Identity** | **E value** | **Specie** | **Acession** | **Genus** | **Family** | **Order** | **Class** | **Phylum** | **Domain** |
|  |  |  |  |  |  |  |  |  |  |  |
| 2 | 100% | *0* | *Marinifilum sp.* | HM466894.1 | *Marinifilum* | Marinifilaceae | Marinilabiliales | Bacteroidia | Bacteroidetes | Bacteria |
| 3 | 100% | *0* | *Prolixibacter sp.* | KU533814.1 | *Prolixibacter* | Prolixibacteraceae | Marinilabiliales | Bacteroidia | Bacteroidetes | Bacteria |
| 4 | 100% | *4e-172* | *Fusobacterium sp* | [HM466896.1](https://www.ncbi.nlm.nih.gov/nucleotide/HM466896.1?report=genbank&log$=nucltop&blast_rank=1&RID=2V27Z4GE013) | *Fusobacterium* | Fusobacteriaceae | [Fusobacteriales](https://www.ncbi.nlm.nih.gov/Taxonomy/Browser/wwwtax.cgi?mode=Undef&id=203491&lvl=3&keep=1&srchmode=1&unlock) | [Fusobacteria](https://www.ncbi.nlm.nih.gov/Taxonomy/Browser/wwwtax.cgi?mode=Undef&id=203490&lvl=3&keep=1&srchmode=1&unlock) | [Fusobacteria](https://es.wikipedia.org/wiki/Fusobacteria) | Bacteria |
| 5 | 100% | *4e-143* | ***Fusobacteriaceae bacterium F3*** | [KT799837.1](https://www.ncbi.nlm.nih.gov/nucleotide/KT799837.1?report=genbank&log$=nucltop&blast_rank=2&RID=2V33KAWC013) | *Fusobacterium* | [Fusobacteriaceae](https://es.wikipedia.org/wiki/Fusobacteriaceae) | [Fusobacteriales](https://www.ncbi.nlm.nih.gov/Taxonomy/Browser/wwwtax.cgi?mode=Undef&id=203491&lvl=3&keep=1&srchmode=1&unlock) | [Fusobacteriia](https://www.ncbi.nlm.nih.gov/Taxonomy/Browser/wwwtax.cgi?mode=Undef&id=203490&lvl=3&keep=1&srchmode=1&unlock) | [Fusobacteria](https://es.wikipedia.org/wiki/Fusobacteria) | Bacteria |
| 6 | 100% | *0* | *Fusobacterium sp* | [HM466898.1](https://www.ncbi.nlm.nih.gov/nucleotide/HM466898.1?report=genbank&log$=nucltop&blast_rank=1&RID=2V3EYWH5013) | *Fusobacterium* | [Fusobacteriaceae](https://es.wikipedia.org/wiki/Fusobacteriaceae) | [Fusobacteriales](https://www.ncbi.nlm.nih.gov/Taxonomy/Browser/wwwtax.cgi?mode=Undef&id=203491&lvl=3&keep=1&srchmode=1&unlock) | [Fusobacteriia](https://www.ncbi.nlm.nih.gov/Taxonomy/Browser/wwwtax.cgi?mode=Undef&id=203490&lvl=3&keep=1&srchmode=1&unlock) | [Fusobacteria](https://es.wikipedia.org/wiki/Fusobacteria) | Bacteria |
| 7 | 100% | *0* | *Fusobacterium sp* | HM466899.1 | *Fusobacterium* | [Fusobacteriaceae](https://es.wikipedia.org/wiki/Fusobacteriaceae) | [Fusobacteriales](https://www.ncbi.nlm.nih.gov/Taxonomy/Browser/wwwtax.cgi?mode=Undef&id=203491&lvl=3&keep=1&srchmode=1&unlock) | [Fusobacteriia](https://www.ncbi.nlm.nih.gov/Taxonomy/Browser/wwwtax.cgi?mode=Undef&id=203490&lvl=3&keep=1&srchmode=1&unlock) | [Fusobacteria](https://es.wikipedia.org/wiki/Fusobacteria) | Bacteria |
| 8 | 100% | *2e-134* | ***Desulfovibrio sp.*** | [HM466900.1](https://www.ncbi.nlm.nih.gov/nucleotide/HM466900.1?report=genbank&log$=nucltop&blast_rank=1&RID=2V42VNKZ013) | *Desulfovibrio* | Desulfovibrionaceae | [Desulfovibrionales](https://en.wikipedia.org/wiki/Desulfovibrionales) | Deltaproteobacteria | [Proteobacteria](https://en.wikipedia.org/wiki/Proteobacteria) | Bacteria |
| 9 | 100% | *0* | ***Desulfovibrio sp*** | [HM466901.1](https://www.ncbi.nlm.nih.gov/nucleotide/HM466901.1?report=genbank&log$=nucltop&blast_rank=1&RID=2V4R9RSF01N) | *Desulfovibrio* | Desulfovibrionaceae | Desulfovibrionales | Deltaproteobacteria | Proteobacteria | Bacteria |
| 10 | 100% | *5e-128* | *Desulfovibrio sp.* | [HM466902.1](https://www.ncbi.nlm.nih.gov/nucleotide/HM466902.1?report=genbank&log$=nucltop&blast_rank=1&RID=37TENAMW013) | *Desulfovibrio* | Desulfovibrionaceae | Desulfovibrionales | Deltaproteobacteria | Proteobacteria | Bacteria |
| 11 | 100% | *2e-109* | *Vibrio parahaemolyticus* | [MT588906.1](https://www.ncbi.nlm.nih.gov/nucleotide/MT588906.1?report=genbank&log$=nucltop&blast_rank=1&RID=37U1XVJ1016) | *Vibrio* | Vibrionaceae | Vibrionales | Gamma Proteobacteria | Proteobacteria | Bacteria |
| 12 | 99.12% | *6e-109* | [*Vibrio owensii*](https://www.ncbi.nlm.nih.gov/Taxonomy/Browser/wwwtax.cgi?mode=Tree&id=696485&lvl=3&keep=1&srchmode=1&unlock) | [HQ161738.1](https://www.ncbi.nlm.nih.gov/nucleotide/HQ161738.1?report=genbank&log$=nucltop&blast_rank=4&RID=37UE5KTV013) | *Vibrio* | Vibrionaceae | Vibrionales | Gamma Proteobacteria | Proteobacteria | Bacteria |
| 13 | 100% | *2e-104* | ***Vibrio sp*** | [HM466905.1](https://www.ncbi.nlm.nih.gov/nucleotide/HM466905.1?report=genbank&log$=nucltop&blast_rank=1&RID=37UXKK96013) | *Vibrio* | Vibrionaceae | Vibrionales | Gamma Proteobacteria | Proteobacteri | Bacteria |
| 14 | 99.13 | *1e-111* | [***Vibrio alginolyticus***](https://www.ncbi.nlm.nih.gov/Taxonomy/Browser/wwwtax.cgi?mode=Info&id=663&lvl=3&lin=f&keep=1&srchmode=1&unlock) | [CP051109.1](https://www.ncbi.nlm.nih.gov/nucleotide/CP051109.1?report=genbank&log$=nucltop&blast_rank=3&RID=37V7WM6W013) | *Vibrio* | [Vibrionaceae](https://en.wikipedia.org/wiki/Vibrionaceae) | [Vibrionales](https://en.wikipedia.org/wiki/Vibrionaceae) | [Gammaproteobacteria](https://en.wikipedia.org/wiki/Gammaproteobacteria) | [Proteobacteria](https://en.wikipedia.org/wiki/Proteobacteria) | [Bacteria](https://en.wikipedia.org/wiki/Bacteria) |
| 15 | 100% | *0* | [***Enterobacter cloacae***](https://www.ncbi.nlm.nih.gov/Taxonomy/Browser/wwwtax.cgi?mode=Info&id=550&lvl=3&lin=f&keep=1&srchmode=1&unlock) | [MT613377.1](https://www.ncbi.nlm.nih.gov/nucleotide/MT613377.1?report=genbank&log$=nucltop&blast_rank=1&RID=37VMXT2P013) | [*Enterobacter*](https://es.wikipedia.org/wiki/Enterobacter) | [Enterobacteriaceae](https://es.wikipedia.org/wiki/Enterobacteriaceae) | [Enterobacterales](https://es.wikipedia.org/wiki/Enterobacterales) | [Gammaproteobacteria](https://es.wikipedia.org/wiki/Gammaproteobacteria) | [Proteobacteria](https://es.wikipedia.org/wiki/Proteobacteria) | [Bacteria](https://es.wikipedia.org/wiki/Bacteria) |
| 16 | 100% | *0* | *Spirochaeta sp* | [HM466912.1](https://www.ncbi.nlm.nih.gov/nucleotide/HM466912.1?report=genbank&log$=nucltop&blast_rank=1&RID=37VYC361016) | *Spirochaeta* | [Spirochaetaceae](https://es.wikipedia.org/wiki/Spirochaetaceae) | [Spirochaetales](https://es.wikipedia.org/wiki/Spirochaetales) | [Spirochaetes](https://es.wikipedia.org/wiki/Spirochaetes) | [Spirochaetes](https://es.wikipedia.org/wiki/Spirochaetes) | [Bacteria](https://es.wikipedia.org/wiki/Bacteria) |
| 17 | 100% | *2e-175* | *Marinilabilia sp* | [HM466913.1](https://www.ncbi.nlm.nih.gov/nucleotide/HM466913.1?report=genbank&log$=nucltop&blast_rank=1&RID=37W98540013) | [*Marinilabilia*](https://www.ncbi.nlm.nih.gov/Taxonomy/Browser/wwwtax.cgi?mode=Undef&id=59738&lvl=3&keep=1&srchmode=1&unlock) | [Marinilabiliaceae](https://www.ncbi.nlm.nih.gov/Taxonomy/Browser/wwwtax.cgi?mode=Undef&id=558415&lvl=3&keep=1&srchmode=1&unlock) | [Marinilabiliales](https://www.ncbi.nlm.nih.gov/Taxonomy/Browser/wwwtax.cgi?mode=Undef&id=1970189&lvl=3&keep=1&srchmode=1&unlock) | [Bacteroidia](https://www.ncbi.nlm.nih.gov/Taxonomy/Browser/wwwtax.cgi?mode=Undef&id=200643&lvl=3&keep=1&srchmode=1&unlock) | [Bacteroidetes](https://www.ncbi.nlm.nih.gov/Taxonomy/Browser/wwwtax.cgi?mode=Undef&id=976&lvl=3&keep=1&srchmode=1&unlock) | Bacteria |
| 18 | 100% | *5e-130* | *Shewanella sp.* | [HM466914.1](https://www.ncbi.nlm.nih.gov/nucleotide/HM466914.1?report=genbank&log$=nucltop&blast_rank=1&RID=37WSDTFD016) | [*Shewanella*](https://www.ncbi.nlm.nih.gov/Taxonomy/Browser/wwwtax.cgi?mode=Undef&id=22&lvl=3&keep=1&srchmode=1&unlock) | [Shewanellaceae](https://www.ncbi.nlm.nih.gov/Taxonomy/Browser/wwwtax.cgi?mode=Undef&id=267890&lvl=3&keep=1&srchmode=1&unlock) | [Alteromonadales](https://www.ncbi.nlm.nih.gov/Taxonomy/Browser/wwwtax.cgi?mode=Undef&id=135622&lvl=3&keep=1&srchmode=1&unlock) | [Gammaproteobacteria](https://www.ncbi.nlm.nih.gov/Taxonomy/Browser/wwwtax.cgi?mode=Undef&id=1236&lvl=3&keep=1&srchmode=1&unlock) | [Proteobacteria](https://www.ncbi.nlm.nih.gov/Taxonomy/Browser/wwwtax.cgi?mode=Undef&id=1224&lvl=3&keep=1&srchmode=1&unlock) | [Bacteria](https://www.ncbi.nlm.nih.gov/Taxonomy/Browser/wwwtax.cgi?mode=Undef&id=2&lvl=3&keep=1&srchmode=1&unlock) |
| 19 | 100% | *3e-96* | [***Escherichia coli***](https://www.ncbi.nlm.nih.gov/Taxonomy/Browser/wwwtax.cgi?mode=Info&id=562&lvl=3&lin=f&keep=1&srchmode=1&unlock) | [AP024205.1](https://www.ncbi.nlm.nih.gov/nucleotide/AP024205.1?report=genbank&log$=nucltop&blast_rank=2&RID=37X07X9E013) | *Escherichia* | Enterobacteriae | [Enterobacterales](https://es.wikipedia.org/wiki/Enterobacterales) | [Gammaproteobacteria](https://es.wikipedia.org/wiki/Gammaproteobacteria) | [Proteobacteria](https://es.wikipedia.org/wiki/Proteobacteria) | Bacteria |
| 20 | 99.39 % | *7e-77* | *Sphingomonas faucium* | [MN918414.1](https://www.ncbi.nlm.nih.gov/nucleotide/MN918414.1?report=genbank&log$=nucltop&blast_rank=14&RID=37XF04JV013) | *Sphingomonas* | [Sphingomonadaceae](https://es.wikipedia.org/wiki/Sphingomonadaceae) | [Sphingomonadales](https://es.wikipedia.org/w/index.php?title=Sphingomonadales&action=edit&redlink=1) | [Alphaproteobacteria](https://es.wikipedia.org/wiki/Alphaproteobacteria) | Proteobacteria | Bacteria |
| 21 | 100% | *1e-80* | *Pseudomonas sp.* | [GU270473.1](https://www.ncbi.nlm.nih.gov/nucleotide/GU270473.1?report=genbank&log$=nucltop&blast_rank=1&RID=37XT04PJ016) | *Pseudomonas* | [Pseudomonadaceae](https://es.wikipedia.org/wiki/Pseudomonadaceae) | Pseudomonales | [Gammaproteobacteria](https://es.wikipedia.org/wiki/Gammaproteobacteria) | [Proteobacteria](https://es.wikipedia.org/wiki/Proteobacteria) | [Bacteria](https://es.wikipedia.org/wiki/Bacteria) |
| 22 | 100% | *3e-94* | *Chryseobacterium sp.* | [JQ404489.1](https://www.ncbi.nlm.nih.gov/nucleotide/JQ404489.1?report=genbank&log$=nucltop&blast_rank=2&RID=37Y5S7BY013) | *Chryseobacterium* | [Flavobacteriaceae](https://es.wikipedia.org/wiki/Flavobacteriaceae) | [Flavobacteriales](https://es.wikipedia.org/wiki/Flavobacteriales) | [Flavobacteria](https://es.wikipedia.org/wiki/Flavobacteria) | [Bacteroidetes](https://es.wikipedia.org/wiki/Bacteroidetes) | [Bacteria](https://es.wikipedia.org/wiki/Bacteria) |
| 23* | 100% | *3e-86* |  | [GU270477.1](https://www.ncbi.nlm.nih.gov/nucleotide/GU270477.1?report=genbank&log$=nucltop&blast_rank=1&RID=37YJMXPF016) | *actinobacterium* |  | [Actinomycetia](https://www.ncbi.nlm.nih.gov/Taxonomy/Browser/wwwtax.cgi?mode=Undef&id=1760&lvl=3&keep=1&srchmode=1&unlock) | Actinobacteria | Actinobacteria | [Bacteria](https://es.wikipedia.org/wiki/Bacteria) |
| 24 | 100% | *2e-83* | *Agrobacterium sp,* | [GU270478.1](https://www.ncbi.nlm.nih.gov/nucleotide/GU270478.1?report=genbank&log$=nucltop&blast_rank=1&RID=37Z26VR2013) | [*Rhizobium*](https://es.wikipedia.org/wiki/Rhizobium_(g%C3%A9nero)) | [Rhizobiaceae](https://es.wikipedia.org/wiki/Rhizobiaceae) | [Rhizobiales](https://es.wikipedia.org/wiki/Rhizobiales) | [Proteobacterias alfa](https://es.wikipedia.org/wiki/Proteobacteria) | [Proteobacteria](https://es.wikipedia.org/wiki/Proteobacteria) | [Bacteria](https://es.wikipedia.org/wiki/Bacteria) |
| 25 | 100% | *1e-80* | [***Pseudomonas alcaligenes***](https://www.ncbi.nlm.nih.gov/Taxonomy/Browser/wwwtax.cgi?mode=Info&id=43263&lvl=3&lin=f&keep=1&srchmode=1&unlock) | [MF079377.1](https://www.ncbi.nlm.nih.gov/nucleotide/MF079377.1?report=genbank&log$=nucltop&blast_rank=1&RID=37ZD3NGP01R) | [*Pseudomonas*](https://en.wikipedia.org/wiki/Pseudomonas) | [Pseudomonadaceae](https://en.wikipedia.org/wiki/Pseudomonadaceae) | [Pseudomonadales](https://en.wikipedia.org/wiki/Pseudomonadales) | [Gammaproteobacteria](https://en.wikipedia.org/wiki/Gammaproteobacteria) | [Proteobacteria](https://en.wikipedia.org/wiki/Proteobacteria) | [Bacteria](https://en.wikipedia.org/wiki/Bacteria) |
| 26 | 100% | *1e-80* | [***Pseudomonas protegens***](https://www.ncbi.nlm.nih.gov/Taxonomy/Browser/wwwtax.cgi?mode=Info&id=380021&lvl=3&lin=f&keep=1&srchmode=1&unlock) | [KY681939.1](https://www.ncbi.nlm.nih.gov/nucleotide/KY681939.1?report=genbank&log$=nucltop&blast_rank=1&RID=37ZMNEZG016) | [*Pseudomonas*](https://en.wikipedia.org/wiki/Pseudomonas) | [Pseudomonadaceae](https://en.wikipedia.org/wiki/Pseudomonadaceae) | [Pseudomonadales](https://en.wikipedia.org/wiki/Pseudomonadales) | [Gammaproteobacteria](https://en.wikipedia.org/wiki/Gammaproteobacteria) | [Proteobacteria](https://en.wikipedia.org/wiki/Proteobacteria) | [Bacteria](https://en.wikipedia.org/wiki/Bacteria) |
| 27 | 100% | *1e-80* | ***Pseudomonas syringae*** | [CP026568.1](https://www.ncbi.nlm.nih.gov/nucleotide/CP026568.1?report=genbank&log$=nucltop&blast_rank=2&RID=37ZTZZ90016) | [*Pseudomonas*](https://en.wikipedia.org/wiki/Pseudomonas) | [Pseudomonadaceae](https://en.wikipedia.org/wiki/Pseudomonadaceae) | [Pseudomonadales](https://en.wikipedia.org/wiki/Pseudomonadales) | [Gammaproteobacteria](https://en.wikipedia.org/wiki/Gammaproteobacteria) | [Proteobacteria](https://en.wikipedia.org/wiki/Proteobacteria) | [Bacteria](https://en.wikipedia.org/wiki/Bacteria) |
| 28 | 90.91% | *3e-67* | ***Methylophilus medardicus*** | [CP040947.1](https://www.ncbi.nlm.nih.gov/nucleotide/CP040947.1?report=genbank&log$=nucltop&blast_rank=6&RID=38049MYM013) | [*Methylophilus*](https://www.ncbi.nlm.nih.gov/Taxonomy/Browser/wwwtax.cgi?mode=Undef&id=16&lvl=3&keep=1&srchmode=1&unlock) | [Methylophilaceae](https://www.ncbi.nlm.nih.gov/Taxonomy/Browser/wwwtax.cgi?mode=Undef&id=32011&lvl=3&keep=1&srchmode=1&unlock) | [Nitrosomonadales](https://www.ncbi.nlm.nih.gov/Taxonomy/Browser/wwwtax.cgi?mode=Undef&id=32003&lvl=3&keep=1&srchmode=1&unlock) | [Betaproteobacteria](https://www.ncbi.nlm.nih.gov/Taxonomy/Browser/wwwtax.cgi?mode=Undef&id=28216&lvl=3&keep=1&srchmode=1&unlock) | [Proteobacteria](https://www.ncbi.nlm.nih.gov/Taxonomy/Browser/wwwtax.cgi?mode=Undef&id=1224&lvl=3&keep=1&srchmode=1&unlock) | Bacteria |
| 29 | 98.91% | *2e-87* | ***Stenotrophomonas acidaminiphila*** | [MT527530.1](https://www.ncbi.nlm.nih.gov/nucleotide/MT527530.1?report=genbank&log$=nucltop&blast_rank=4&RID=380C5UGR016) | [*Stenotrophomonas*](https://www.ncbi.nlm.nih.gov/Taxonomy/Browser/wwwtax.cgi?mode=Undef&id=40323&lvl=3&keep=1&srchmode=1&unlock) | [Xanthomonadaceae](https://www.ncbi.nlm.nih.gov/Taxonomy/Browser/wwwtax.cgi?mode=Undef&id=32033&lvl=3&keep=1&srchmode=1&unlock) | [Xanthomonadales](https://www.ncbi.nlm.nih.gov/Taxonomy/Browser/wwwtax.cgi?mode=Undef&id=135614&lvl=3&keep=1&srchmode=1&unlock) | [Gammaproteobacteria](https://www.ncbi.nlm.nih.gov/Taxonomy/Browser/wwwtax.cgi?mode=Undef&id=1236&lvl=3&keep=1&srchmode=1&unlock) | [Roteobacteria](https://www.ncbi.nlm.nih.gov/Taxonomy/Browser/wwwtax.cgi?mode=Undef&id=1224&lvl=3&keep=1&srchmode=1&unlock) | [Bacteria](https://www.ncbi.nlm.nih.gov/Taxonomy/Browser/wwwtax.cgi?mode=Undef&id=2&lvl=3&keep=1&srchmode=1&unlock) |
| 30 | 94.41% | *1e-53* | ***Neorhizobium galegae*** | [MH671634.1](https://www.ncbi.nlm.nih.gov/nucleotide/MH671634.1?report=genbank&log$=nucltop&blast_rank=5&RID=380N4672016) | [*Neorhizobium*](https://www.ncbi.nlm.nih.gov/Taxonomy/Browser/wwwtax.cgi?mode=Undef&id=1525371&lvl=3&keep=1&srchmode=1&unlock) | [Rhizobiaceae](https://www.ncbi.nlm.nih.gov/Taxonomy/Browser/wwwtax.cgi?mode=Undef&id=82115&lvl=3&keep=1&srchmode=1&unlock) | [Rhizobiales](https://www.ncbi.nlm.nih.gov/Taxonomy/Browser/wwwtax.cgi?mode=Undef&id=356&lvl=3&keep=1&srchmode=1&unlock) | [Alphaproteobacteria](https://www.ncbi.nlm.nih.gov/Taxonomy/Browser/wwwtax.cgi?mode=Undef&id=28211&lvl=3&keep=1&srchmode=1&unlock) | [Proteobacteria](https://www.ncbi.nlm.nih.gov/Taxonomy/Browser/wwwtax.cgi?mode=Undef&id=1224&lvl=3&keep=1&srchmode=1&unlock) | [Bacteria](https://www.ncbi.nlm.nih.gov/Taxonomy/Browser/wwwtax.cgi?mode=Undef&id=2&lvl=3&keep=1&srchmode=1&unlock) |
| 31 | 100% | *1e-80* | ***Methylophilus sp*** | [AB539841.1](https://www.ncbi.nlm.nih.gov/nucleotide/AB539841.1?report=genbank&log$=nucltop&blast_rank=4&RID=3832WX0S01R) | [*Methylophilus*](https://www.ncbi.nlm.nih.gov/Taxonomy/Browser/wwwtax.cgi?mode=Undef&id=16&lvl=3&keep=1&srchmode=1&unlock) | [Methylophilaceae](https://www.ncbi.nlm.nih.gov/Taxonomy/Browser/wwwtax.cgi?mode=Undef&id=32011&lvl=3&keep=1&srchmode=1&unlock) | [Nitrosomonadales](https://www.ncbi.nlm.nih.gov/Taxonomy/Browser/wwwtax.cgi?mode=Undef&id=32003&lvl=3&keep=1&srchmode=1&unlock) | [Betaproteobacteria](https://www.ncbi.nlm.nih.gov/Taxonomy/Browser/wwwtax.cgi?mode=Undef&id=28216&lvl=3&keep=1&srchmode=1&unlock) | [Proteobacteria](https://www.ncbi.nlm.nih.gov/Taxonomy/Browser/wwwtax.cgi?mode=Undef&id=1224&lvl=3&keep=1&srchmode=1&unlock) | [Bacteria](https://www.ncbi.nlm.nih.gov/Taxonomy/Browser/wwwtax.cgi?mode=Undef&id=2&lvl=3&keep=1&srchmode=1&unlock) |
| 32 | 100% | *3e-96* | ***Escherichia coli*** | [AP024205.1](https://www.ncbi.nlm.nih.gov/nucleotide/AP024205.1?report=genbank&log$=nucltop&blast_rank=2&RID=383DZYMR016) | [*Escherichia*](https://www.ncbi.nlm.nih.gov/Taxonomy/Browser/wwwtax.cgi?mode=Undef&id=561&lvl=3&keep=1&srchmode=1&unlock) | [Enterobacteriaceae](https://www.ncbi.nlm.nih.gov/Taxonomy/Browser/wwwtax.cgi?mode=Undef&id=543&lvl=3&keep=1&srchmode=1&unlock) | [Enterobacterales](https://www.ncbi.nlm.nih.gov/Taxonomy/Browser/wwwtax.cgi?mode=Undef&id=91347&lvl=3&keep=1&srchmode=1&unlock) | [Gammaproteobacteria](https://www.ncbi.nlm.nih.gov/Taxonomy/Browser/wwwtax.cgi?mode=Undef&id=1236&lvl=3&keep=1&srchmode=1&unlock) | [Proteobacteria](https://www.ncbi.nlm.nih.gov/Taxonomy/Browser/wwwtax.cgi?mode=Undef&id=1224&lvl=3&keep=1&srchmode=1&unlock) | [Bacteria](https://www.ncbi.nlm.nih.gov/Taxonomy/Browser/wwwtax.cgi?mode=Undef&id=2&lvl=3&keep=1&srchmode=1&unlock) |
| 33 | 98% | *1e-80* | ***Bdellovibrio bacteriovorus*** | [MK779947.1](https://www.ncbi.nlm.nih.gov/nucleotide/MK779947.1?report=genbank&log$=nucltop&blast_rank=6&RID=383NHBGP01R) | [*Bdellovibrio*](https://www.ncbi.nlm.nih.gov/Taxonomy/Browser/wwwtax.cgi?mode=Undef&id=958&lvl=3&keep=1&srchmode=1&unlock) | [Bdellovibrionaceae](https://www.ncbi.nlm.nih.gov/Taxonomy/Browser/wwwtax.cgi?mode=Undef&id=213483&lvl=3&keep=1&srchmode=1&unlock) | [Bdellovibrionales](https://www.ncbi.nlm.nih.gov/Taxonomy/Browser/wwwtax.cgi?mode=Undef&id=213481&lvl=3&keep=1&srchmode=1&unlock) | [Oligoflexia](https://www.ncbi.nlm.nih.gov/Taxonomy/Browser/wwwtax.cgi?mode=Undef&id=1553900&lvl=3&keep=1&srchmode=1&unlock) | [Proteobacteria](https://www.ncbi.nlm.nih.gov/Taxonomy/Browser/wwwtax.cgi?mode=Undef&id=1224&lvl=3&keep=1&srchmode=1&unlock) | [Bacteria](https://www.ncbi.nlm.nih.gov/Taxonomy/Browser/wwwtax.cgi?mode=Undef&id=2&lvl=3&keep=1&srchmode=1&unlock) |
| 34 | 100% | *5e-84* | ***Bdellovibrio bacteriovorus*** | [MK779947.1](https://www.ncbi.nlm.nih.gov/nucleotide/MK779947.1?report=genbank&log$=nucltop&blast_rank=5&RID=383YWVP9013) | [*Bdellovibrio*](https://www.ncbi.nlm.nih.gov/Taxonomy/Browser/wwwtax.cgi?mode=Undef&id=958&lvl=3&keep=1&srchmode=1&unlock) | [Bdellovibrionaceae](https://www.ncbi.nlm.nih.gov/Taxonomy/Browser/wwwtax.cgi?mode=Undef&id=213483&lvl=3&keep=1&srchmode=1&unlock) | [Bdellovibrionales](https://www.ncbi.nlm.nih.gov/Taxonomy/Browser/wwwtax.cgi?mode=Undef&id=213481&lvl=3&keep=1&srchmode=1&unlock) | [Oligoflexia](https://www.ncbi.nlm.nih.gov/Taxonomy/Browser/wwwtax.cgi?mode=Undef&id=1553900&lvl=3&keep=1&srchmode=1&unlock) | [Proteobacteria](https://www.ncbi.nlm.nih.gov/Taxonomy/Browser/wwwtax.cgi?mode=Undef&id=1224&lvl=3&keep=1&srchmode=1&unlock) | [Bacteria](https://www.ncbi.nlm.nih.gov/Taxonomy/Browser/wwwtax.cgi?mode=Undef&id=2&lvl=3&keep=1&srchmode=1&unlock) |
| 35 | 99% | *1e-95* | ***Kluyvera ascorbata*** | [MT611553.1](https://www.ncbi.nlm.nih.gov/nucleotide/MT611553.1?report=genbank&log$=nucltop&blast_rank=2&RID=3844GM2S016) | [*Kluyvera*](https://www.ncbi.nlm.nih.gov/Taxonomy/Browser/wwwtax.cgi?mode=Undef&id=579&lvl=3&keep=1&srchmode=1&unlock) | [Enterobacteriaceae](https://www.ncbi.nlm.nih.gov/Taxonomy/Browser/wwwtax.cgi?mode=Undef&id=543&lvl=3&keep=1&srchmode=1&unlock) | [Enterobacterales](https://www.ncbi.nlm.nih.gov/Taxonomy/Browser/wwwtax.cgi?mode=Undef&id=91347&lvl=3&keep=1&srchmode=1&unlock) | [Gammaproteobacteria](https://www.ncbi.nlm.nih.gov/Taxonomy/Browser/wwwtax.cgi?mode=Undef&id=1236&lvl=3&keep=1&srchmode=1&unlock) | [Proteobacteria](https://www.ncbi.nlm.nih.gov/Taxonomy/Browser/wwwtax.cgi?mode=Undef&id=1224&lvl=3&keep=1&srchmode=1&unlock) | [Bacteria](https://www.ncbi.nlm.nih.gov/Taxonomy/Browser/wwwtax.cgi?mode=Undef&id=2&lvl=3&keep=1&srchmode=1&unlock) |
| 36 | 98.47% | *6e-57* | ***Acinetobacter tjernbergiae*** | [MN901261.1](https://www.ncbi.nlm.nih.gov/nucleotide/MN901261.1?report=genbank&log$=nucltop&blast_rank=4&RID=384C4C4F01R) | [*Acinetobacter*](https://www.ncbi.nlm.nih.gov/Taxonomy/Browser/wwwtax.cgi?mode=Undef&id=469&lvl=3&keep=1&srchmode=1&unlock) | [Moraxellaceae](https://www.ncbi.nlm.nih.gov/Taxonomy/Browser/wwwtax.cgi?mode=Undef&id=468&lvl=3&keep=1&srchmode=1&unlock) | [Pseudomonadales](https://www.ncbi.nlm.nih.gov/Taxonomy/Browser/wwwtax.cgi?mode=Undef&id=72274&lvl=3&keep=1&srchmode=1&unlock) | [Gammaproteobacteria](https://www.ncbi.nlm.nih.gov/Taxonomy/Browser/wwwtax.cgi?mode=Undef&id=1236&lvl=3&keep=1&srchmode=1&unlock) | [Proteobacteria](https://www.ncbi.nlm.nih.gov/Taxonomy/Browser/wwwtax.cgi?mode=Undef&id=1224&lvl=3&keep=1&srchmode=1&unlock) | [Bacteria](https://www.ncbi.nlm.nih.gov/Taxonomy/Browser/wwwtax.cgi?mode=Undef&id=2&lvl=3&keep=1&srchmode=1&unlock) |
| 37 | 100% | *1e-63* | ***Acinetobacter tjernbergiae*** | [MN901261.1](https://www.ncbi.nlm.nih.gov/nucleotide/MN901261.1?report=genbank&log$=nucltop&blast_rank=3&RID=384KM7BV01R) | [*Acinetobacter*](https://www.ncbi.nlm.nih.gov/Taxonomy/Browser/wwwtax.cgi?mode=Undef&id=469&lvl=3&keep=1&srchmode=1&unlock) | [Moraxellaceae](https://www.ncbi.nlm.nih.gov/Taxonomy/Browser/wwwtax.cgi?mode=Undef&id=468&lvl=3&keep=1&srchmode=1&unlock) | [Pseudomonadales](https://www.ncbi.nlm.nih.gov/Taxonomy/Browser/wwwtax.cgi?mode=Undef&id=72274&lvl=3&keep=1&srchmode=1&unlock) | [Gammaproteobacteria](https://www.ncbi.nlm.nih.gov/Taxonomy/Browser/wwwtax.cgi?mode=Undef&id=1236&lvl=3&keep=1&srchmode=1&unlock) | [Proteobacteria](https://www.ncbi.nlm.nih.gov/Taxonomy/Browser/wwwtax.cgi?mode=Undef&id=1224&lvl=3&keep=1&srchmode=1&unlock) | [Bacteria](https://www.ncbi.nlm.nih.gov/Taxonomy/Browser/wwwtax.cgi?mode=Undef&id=2&lvl=3&keep=1&srchmode=1&unlock) |
| 38 | 100% | *7e-103* | ***Bacillus coreaensis*** | [MT214194.1](https://www.ncbi.nlm.nih.gov/nucleotide/MT214194.1?report=genbank&log$=nucltop&blast_rank=1&RID=384U39EG013) | [*Bacillus*](https://www.ncbi.nlm.nih.gov/Taxonomy/Browser/wwwtax.cgi?mode=Undef&id=1386&lvl=3&keep=1&srchmode=1&unlock) | [Bacillaceae](https://www.ncbi.nlm.nih.gov/Taxonomy/Browser/wwwtax.cgi?mode=Undef&id=186817&lvl=3&keep=1&srchmode=1&unlock) | [Bacillales](https://www.ncbi.nlm.nih.gov/Taxonomy/Browser/wwwtax.cgi?mode=Undef&id=1385&lvl=3&keep=1&srchmode=1&unlock) | [Bacilli](https://www.ncbi.nlm.nih.gov/Taxonomy/Browser/wwwtax.cgi?mode=Undef&id=91061&lvl=3&keep=1&srchmode=1&unlock) | [Firmicutes](https://www.ncbi.nlm.nih.gov/Taxonomy/Browser/wwwtax.cgi?mode=Undef&id=1239&lvl=3&keep=1&srchmode=1&unlock) | [Bacteria](https://www.ncbi.nlm.nih.gov/Taxonomy/Browser/wwwtax.cgi?mode=Undef&id=2&lvl=3&keep=1&srchmode=1&unlock) |
| 39 | 100% | *2e-93* | ***Sphingobacterium sp.*** | [LN867309.1](https://www.ncbi.nlm.nih.gov/nucleotide/LN867309.1?report=genbank&log$=nucltop&blast_rank=1&RID=385509Z5013) | [*Sphingobacterium*](https://www.ncbi.nlm.nih.gov/Taxonomy/Browser/wwwtax.cgi?mode=Undef&id=2609468&lvl=3&keep=1&srchmode=1&unlock) | [Sphingobacteriaceae](https://www.ncbi.nlm.nih.gov/Taxonomy/Browser/wwwtax.cgi?mode=Undef&id=84566&lvl=3&keep=1&srchmode=1&unlock) | [Sphingobacteriales](https://www.ncbi.nlm.nih.gov/Taxonomy/Browser/wwwtax.cgi?mode=Undef&id=200666&lvl=3&keep=1&srchmode=1&unlock) | [Sphingobacteriia](https://www.ncbi.nlm.nih.gov/Taxonomy/Browser/wwwtax.cgi?mode=Undef&id=117747&lvl=3&keep=1&srchmode=1&unlock) | [Bacteroidetes](https://www.ncbi.nlm.nih.gov/Taxonomy/Browser/wwwtax.cgi?mode=Undef&id=68336&lvl=3&keep=1&srchmode=1&unlock) | [Bacteria](https://www.ncbi.nlm.nih.gov/Taxonomy/Browser/wwwtax.cgi?mode=Undef&id=2&lvl=3&keep=1&srchmode=1&unlock) |
| 40 | *99%* | *2e-73* | ***Acinetobacter tjernbergiae*** | [MN901261.1](https://www.ncbi.nlm.nih.gov/nucleotide/MN901261.1?report=genbank&log$=nucltop&blast_rank=3&RID=385C44TS01R) | [*Acinetobacter*](https://www.ncbi.nlm.nih.gov/Taxonomy/Browser/wwwtax.cgi?mode=Undef&id=469&lvl=3&keep=1&srchmode=1&unlock) | [Moraxellaceae](https://www.ncbi.nlm.nih.gov/Taxonomy/Browser/wwwtax.cgi?mode=Undef&id=468&lvl=3&keep=1&srchmode=1&unlock) | [Pseudomonadales](https://www.ncbi.nlm.nih.gov/Taxonomy/Browser/wwwtax.cgi?mode=Undef&id=72274&lvl=3&keep=1&srchmode=1&unlock) | [Gammaproteobacteria](https://www.ncbi.nlm.nih.gov/Taxonomy/Browser/wwwtax.cgi?mode=Undef&id=1236&lvl=3&keep=1&srchmode=1&unlock) | [Proteobacteria](https://www.ncbi.nlm.nih.gov/Taxonomy/Browser/wwwtax.cgi?mode=Undef&id=1224&lvl=3&keep=1&srchmode=1&unlock) | [Bacteria](https://www.ncbi.nlm.nih.gov/Taxonomy/Browser/wwwtax.cgi?mode=Undef&id=2&lvl=3&keep=1&srchmode=1&unlock) |
| 41 | 100% | *7e-77* | ***Acinetobacter tjernbergiae*** | [MN901261.1](https://www.ncbi.nlm.nih.gov/nucleotide/MN901261.1?report=genbank&log$=nucltop&blast_rank=2&RID=385NXEM0016) | [*Acinetobacter*](https://www.ncbi.nlm.nih.gov/Taxonomy/Browser/wwwtax.cgi?mode=Undef&id=469&lvl=3&keep=1&srchmode=1&unlock) | [Moraxellaceae](https://www.ncbi.nlm.nih.gov/Taxonomy/Browser/wwwtax.cgi?mode=Undef&id=468&lvl=3&keep=1&srchmode=1&unlock) | [Pseudomonadales](https://www.ncbi.nlm.nih.gov/Taxonomy/Browser/wwwtax.cgi?mode=Undef&id=72274&lvl=3&keep=1&srchmode=1&unlock) | [Gammaproteobacteria](https://www.ncbi.nlm.nih.gov/Taxonomy/Browser/wwwtax.cgi?mode=Undef&id=1236&lvl=3&keep=1&srchmode=1&unlock) | [Proteobacteria](https://www.ncbi.nlm.nih.gov/Taxonomy/Browser/wwwtax.cgi?mode=Undef&id=1224&lvl=3&keep=1&srchmode=1&unlock) | [Bacteria](https://www.ncbi.nlm.nih.gov/Taxonomy/Browser/wwwtax.cgi?mode=Undef&id=2&lvl=3&keep=1&srchmode=1&unlock) |
| 42 | 100% | *2e-96* | ***Bacillus koreensis*** | [MT225671.1](https://www.ncbi.nlm.nih.gov/nucleotide/MT225671.1?report=genbank&log$=nucltop&blast_rank=1&RID=385UZSR2016) | ***Bacillus*** | [Bacillaceae](https://www.ncbi.nlm.nih.gov/Taxonomy/Browser/wwwtax.cgi?mode=Undef&id=186817&lvl=3&keep=1&srchmode=1&unlock) | [Bacillales](https://www.ncbi.nlm.nih.gov/Taxonomy/Browser/wwwtax.cgi?mode=Undef&id=1385&lvl=3&keep=1&srchmode=1&unlock) | [Bacilli](https://www.ncbi.nlm.nih.gov/Taxonomy/Browser/wwwtax.cgi?mode=Undef&id=91061&lvl=3&keep=1&srchmode=1&unlock) | [Firmicutes](https://www.ncbi.nlm.nih.gov/Taxonomy/Browser/wwwtax.cgi?mode=Undef&id=1239&lvl=3&keep=1&srchmode=1&unlock) | [Bacteria](https://www.ncbi.nlm.nih.gov/Taxonomy/Browser/wwwtax.cgi?mode=Undef&id=2&lvl=3&keep=1&srchmode=1&unlock) |
| 43 | 98.83% | *0* | ***Moraxella osloensis*** | [MT225646.1](https://www.ncbi.nlm.nih.gov/nucleotide/MT225646.1?report=genbank&log$=nucltop&blast_rank=7&RID=3868DXJ1016) | [*Moraxella*](https://www.ncbi.nlm.nih.gov/Taxonomy/Browser/wwwtax.cgi?mode=Undef&id=475&lvl=3&keep=1&srchmode=1&unlock) | [Moraxellaceae](https://www.ncbi.nlm.nih.gov/Taxonomy/Browser/wwwtax.cgi?mode=Undef&id=468&lvl=3&keep=1&srchmode=1&unlock) | [Pseudomonadales](https://www.ncbi.nlm.nih.gov/Taxonomy/Browser/wwwtax.cgi?mode=Undef&id=72274&lvl=3&keep=1&srchmode=1&unlock) | [Gammaproteobacteria](https://www.ncbi.nlm.nih.gov/Taxonomy/Browser/wwwtax.cgi?mode=Undef&id=1236&lvl=3&keep=1&srchmode=1&unlock) | [Proteobacteria](https://www.ncbi.nlm.nih.gov/Taxonomy/Browser/wwwtax.cgi?mode=Undef&id=1224&lvl=3&keep=1&srchmode=1&unlock) | [Bacteria](https://www.ncbi.nlm.nih.gov/Taxonomy/Browser/wwwtax.cgi?mode=Undef&id=2&lvl=3&keep=1&srchmode=1&unlock) |
| 44 | 92.32% | *0* | ***Moraxella osloensis*** | [KJ589468.1](https://www.ncbi.nlm.nih.gov/nucleotide/KJ589468.1?report=genbank&log$=nucltop&blast_rank=16&RID=386KCX9M01R) | [*Moraxella*](https://www.ncbi.nlm.nih.gov/Taxonomy/Browser/wwwtax.cgi?mode=Undef&id=475&lvl=3&keep=1&srchmode=1&unlock) | [Moraxellaceae](https://www.ncbi.nlm.nih.gov/Taxonomy/Browser/wwwtax.cgi?mode=Undef&id=468&lvl=3&keep=1&srchmode=1&unlock) | [Pseudomonadales](https://www.ncbi.nlm.nih.gov/Taxonomy/Browser/wwwtax.cgi?mode=Undef&id=72274&lvl=3&keep=1&srchmode=1&unlock) | [Gammaproteobacteria](https://www.ncbi.nlm.nih.gov/Taxonomy/Browser/wwwtax.cgi?mode=Undef&id=1236&lvl=3&keep=1&srchmode=1&unlock) | [Proteobacteria](https://www.ncbi.nlm.nih.gov/Taxonomy/Browser/wwwtax.cgi?mode=Undef&id=1224&lvl=3&keep=1&srchmode=1&unlock) | [Bacteria](https://www.ncbi.nlm.nih.gov/Taxonomy/Browser/wwwtax.cgi?mode=Undef&id=2&lvl=3&keep=1&srchmode=1&unlock) |
| 45 | *99%* | *0* | ***Pseudomonas fragi*** | [MN758766.1](https://www.ncbi.nlm.nih.gov/nucleotide/MN758766.1?report=genbank&log$=nucltop&blast_rank=2&RID=386UNVWZ01R) | [*Pseudomonas*](https://www.ncbi.nlm.nih.gov/Taxonomy/Browser/wwwtax.cgi?mode=Undef&id=286&lvl=3&keep=1&srchmode=1&unlock) | [Pseudomonadaceae](https://www.ncbi.nlm.nih.gov/Taxonomy/Browser/wwwtax.cgi?mode=Undef&id=135621&lvl=3&keep=1&srchmode=1&unlock) | [Pseudomonadales](https://www.ncbi.nlm.nih.gov/Taxonomy/Browser/wwwtax.cgi?mode=Undef&id=72274&lvl=3&keep=1&srchmode=1&unlock) | [Gammaproteobacteria](https://www.ncbi.nlm.nih.gov/Taxonomy/Browser/wwwtax.cgi?mode=Undef&id=1236&lvl=3&keep=1&srchmode=1&unlock) | [Proteobacteria](https://www.ncbi.nlm.nih.gov/Taxonomy/Browser/wwwtax.cgi?mode=Undef&id=1224&lvl=3&keep=1&srchmode=1&unlock) | [Bacteria](https://www.ncbi.nlm.nih.gov/Taxonomy/Browser/wwwtax.cgi?mode=Undef&id=2&lvl=3&keep=1&srchmode=1&unlock) |
| 46 | *99%* | *0* | ***Pseudomonas fragi*** | [MN758766.1](https://www.ncbi.nlm.nih.gov/nucleotide/MN758766.1?report=genbank&log$=nucltop&blast_rank=2&RID=3874WC2A01R) | [*Pseudomonas*](https://www.ncbi.nlm.nih.gov/Taxonomy/Browser/wwwtax.cgi?mode=Undef&id=286&lvl=3&keep=1&srchmode=1&unlock) | [Pseudomonadaceae](https://www.ncbi.nlm.nih.gov/Taxonomy/Browser/wwwtax.cgi?mode=Undef&id=135621&lvl=3&keep=1&srchmode=1&unlock) | [Pseudomonadales](https://www.ncbi.nlm.nih.gov/Taxonomy/Browser/wwwtax.cgi?mode=Undef&id=72274&lvl=3&keep=1&srchmode=1&unlock) | [Gammaproteobacteria](https://www.ncbi.nlm.nih.gov/Taxonomy/Browser/wwwtax.cgi?mode=Undef&id=1236&lvl=3&keep=1&srchmode=1&unlock) | [Proteobacteria](https://www.ncbi.nlm.nih.gov/Taxonomy/Browser/wwwtax.cgi?mode=Undef&id=1224&lvl=3&keep=1&srchmode=1&unlock) | [Bacteria](https://www.ncbi.nlm.nih.gov/Taxonomy/Browser/wwwtax.cgi?mode=Undef&id=2&lvl=3&keep=1&srchmode=1&unlock) |
| 47 | 99.36% | *0* | ***Pseudomonas poae*** | [MN307314.1](https://www.ncbi.nlm.nih.gov/nucleotide/MN307314.1?report=genbank&log$=nucltop&blast_rank=24&RID=387C8XGE016) | [*Pseudomonas*](https://www.ncbi.nlm.nih.gov/Taxonomy/Browser/wwwtax.cgi?mode=Undef&id=286&lvl=3&keep=1&srchmode=1&unlock) | [Pseudomonadaceae](https://www.ncbi.nlm.nih.gov/Taxonomy/Browser/wwwtax.cgi?mode=Undef&id=135621&lvl=3&keep=1&srchmode=1&unlock) | [Pseudomonadales](https://www.ncbi.nlm.nih.gov/Taxonomy/Browser/wwwtax.cgi?mode=Undef&id=72274&lvl=3&keep=1&srchmode=1&unlock) | [Gammaproteobacteria](https://www.ncbi.nlm.nih.gov/Taxonomy/Browser/wwwtax.cgi?mode=Undef&id=1236&lvl=3&keep=1&srchmode=1&unlock) | [Proteobacteria](https://www.ncbi.nlm.nih.gov/Taxonomy/Browser/wwwtax.cgi?mode=Undef&id=1224&lvl=3&keep=1&srchmode=1&unlock) | [Bacteria](https://www.ncbi.nlm.nih.gov/Taxonomy/Browser/wwwtax.cgi?mode=Undef&id=2&lvl=3&keep=1&srchmode=1&unlock) |
| 48 | 96.85% | *0* | ***Gallionella capsiferriformans*** | [NR_074658.1](https://www.ncbi.nlm.nih.gov/nucleotide/NR_074658.1?report=genbank&log$=nucltop&blast_rank=68&RID=387N1E7601R) | [*Gallionella*](https://www.ncbi.nlm.nih.gov/Taxonomy/Browser/wwwtax.cgi?mode=Undef&id=96&lvl=3&keep=1&srchmode=1&unlock) | [Gallionellaceae](https://www.ncbi.nlm.nih.gov/Taxonomy/Browser/wwwtax.cgi?mode=Undef&id=90627&lvl=3&keep=1&srchmode=1&unlock) | [Nitrosomonadales](https://www.ncbi.nlm.nih.gov/Taxonomy/Browser/wwwtax.cgi?mode=Undef&id=32003&lvl=3&keep=1&srchmode=1&unlock) | [Betaproteobacteria](https://www.ncbi.nlm.nih.gov/Taxonomy/Browser/wwwtax.cgi?mode=Undef&id=28216&lvl=3&keep=1&srchmode=1&unlock) | [Proteobacteria](https://www.ncbi.nlm.nih.gov/Taxonomy/Browser/wwwtax.cgi?mode=Undef&id=1224&lvl=3&keep=1&srchmode=1&unlock) | [Bacteria](https://www.ncbi.nlm.nih.gov/Taxonomy/Browser/wwwtax.cgi?mode=Undef&id=2&lvl=3&keep=1&srchmode=1&unlock) |
| 49 | 93.88% | *0* | ***Methylobacter sp.*** | [HF565143.1](https://www.ncbi.nlm.nih.gov/nucleotide/HF565143.1?report=genbank&log$=nucltop&blast_rank=2&RID=387ZG5HJ016) | [*Methylobacter*](https://www.ncbi.nlm.nih.gov/Taxonomy/Browser/wwwtax.cgi?mode=Undef&id=429&lvl=3&keep=1&srchmode=1&unlock) | [Methylococcaceae](https://www.ncbi.nlm.nih.gov/Taxonomy/Browser/wwwtax.cgi?mode=Undef&id=403&lvl=3&keep=1&srchmode=1&unlock) | [Methylococcales](https://www.ncbi.nlm.nih.gov/Taxonomy/Browser/wwwtax.cgi?mode=Undef&id=135618&lvl=3&keep=1&srchmode=1&unlock) | [Gammaproteobacteria](https://www.ncbi.nlm.nih.gov/Taxonomy/Browser/wwwtax.cgi?mode=Undef&id=1236&lvl=3&keep=1&srchmode=1&unlock) | [Proteobacteria](https://www.ncbi.nlm.nih.gov/Taxonomy/Browser/wwwtax.cgi?mode=Undef&id=1224&lvl=3&keep=1&srchmode=1&unlock) | [Bacteria](https://www.ncbi.nlm.nih.gov/Taxonomy/Browser/wwwtax.cgi?mode=Undef&id=2&lvl=3&keep=1&srchmode=1&unlock) |
| 50 | 96.63% | *0* | ***Steroidobacter denitrificans*** | [CP011971.1](https://www.ncbi.nlm.nih.gov/nucleotide/CP011971.1?report=genbank&log$=nucltop&blast_rank=23&RID=388W46S701R) | [*Steroidobacter*](https://www.ncbi.nlm.nih.gov/Taxonomy/Browser/wwwtax.cgi?mode=Undef&id=469322&lvl=3&keep=1&srchmode=1&unlock) | [Steroidobacteraceae](https://www.ncbi.nlm.nih.gov/Taxonomy/Browser/wwwtax.cgi?mode=Undef&id=2689614&lvl=3&keep=1&srchmode=1&unlock) | [Nevskiales](https://www.ncbi.nlm.nih.gov/Taxonomy/Browser/wwwtax.cgi?mode=Undef&id=1775403&lvl=3&keep=1&srchmode=1&unlock) | [Gammaproteobacteria](https://www.ncbi.nlm.nih.gov/Taxonomy/Browser/wwwtax.cgi?mode=Undef&id=1236&lvl=3&keep=1&srchmode=1&unlock) | [Proteobacteria](https://www.ncbi.nlm.nih.gov/Taxonomy/Browser/wwwtax.cgi?mode=Undef&id=1224&lvl=3&keep=1&srchmode=1&unlock) | [Bacteria](https://www.ncbi.nlm.nih.gov/Taxonomy/Browser/wwwtax.cgi?mode=Undef&id=2&lvl=3&keep=1&srchmode=1&unlock) |
| 51* | 100% | *0* |  | [GU911375.1](https://www.ncbi.nlm.nih.gov/nucleotide/GU911375.1?report=genbank&log$=nucltop&blast_rank=1&RID=3898R09901R) |  |  |  |  | [Bacteroidetes](https://www.ncbi.nlm.nih.gov/Taxonomy/Browser/wwwtax.cgi?mode=Undef&id=68336&lvl=3&keep=1&srchmode=1&unlock) | [Bacteria](https://www.ncbi.nlm.nih.gov/Taxonomy/Browser/wwwtax.cgi?mode=Undef&id=2&lvl=3&keep=1&srchmode=1&unlock) |
| 52* | 98.53% | *0* |  | [EF018502.1](https://www.ncbi.nlm.nih.gov/nucleotide/EF018502.1?report=genbank&log$=nucltop&blast_rank=23&RID=389HA3BA016) |  | [Nitrosomonadaceae](https://www.ncbi.nlm.nih.gov/Taxonomy/Browser/wwwtax.cgi?mode=Undef&id=206379&lvl=3&keep=1&srchmode=1&unlock) | [Nitrosomonadales](https://www.ncbi.nlm.nih.gov/Taxonomy/Browser/wwwtax.cgi?mode=Undef&id=32003&lvl=3&keep=1&srchmode=1&unlock) | [Betaproteobacteria](https://www.ncbi.nlm.nih.gov/Taxonomy/Browser/wwwtax.cgi?mode=Undef&id=28216&lvl=3&keep=1&srchmode=1&unlock) | [Proteobacteria](https://www.ncbi.nlm.nih.gov/Taxonomy/Browser/wwwtax.cgi?mode=Undef&id=1224&lvl=3&keep=1&srchmode=1&unlock) | [Bacteria](https://www.ncbi.nlm.nih.gov/Taxonomy/Browser/wwwtax.cgi?mode=Undef&id=2&lvl=3&keep=1&srchmode=1&unlock) |
| 53* | 100% | *0* |  | [GU911377.1](https://www.ncbi.nlm.nih.gov/nucleotide/GU911377.1?report=genbank&log$=nucltop&blast_rank=1&RID=38A1HFPH013) |  |  |  | [Deltaproteobacteria](https://www.ncbi.nlm.nih.gov/Taxonomy/Browser/wwwtax.cgi?mode=Undef&id=28221&lvl=3&keep=1&srchmode=1&unlock) | [Proteobacteria](https://www.ncbi.nlm.nih.gov/Taxonomy/Browser/wwwtax.cgi?mode=Undef&id=1224&lvl=3&keep=1&srchmode=1&unlock) | [Bacteria](https://www.ncbi.nlm.nih.gov/Taxonomy/Browser/wwwtax.cgi?mode=Undef&id=2&lvl=3&keep=1&srchmode=1&unlock) |
| 54 | 100% | *0* | ***Magnetobacterium sp*** | [GU911380.1](https://www.ncbi.nlm.nih.gov/nucleotide/GU911380.1?report=genbank&log$=nucltop&blast_rank=1&RID=38ACXSGM013) | [*Magnetobacterium*](https://www.ncbi.nlm.nih.gov/Taxonomy/Browser/wwwtax.cgi?mode=Undef&id=40118&lvl=3&keep=1&srchmode=1&unlock) | [Nitrospiraceae](https://www.ncbi.nlm.nih.gov/Taxonomy/Browser/wwwtax.cgi?mode=Undef&id=189779&lvl=3&keep=1&srchmode=1&unlock) | [Nitrospirales](https://www.ncbi.nlm.nih.gov/Taxonomy/Browser/wwwtax.cgi?mode=Undef&id=189778&lvl=3&keep=1&srchmode=1&unlock) | [Nitrospira](https://www.ncbi.nlm.nih.gov/Taxonomy/Browser/wwwtax.cgi?mode=Undef&id=203693&lvl=3&keep=1&srchmode=1&unlock) | [Nitrospirae](https://www.ncbi.nlm.nih.gov/Taxonomy/Browser/wwwtax.cgi?mode=Undef&id=40117&lvl=3&keep=1&srchmode=1&unlock) | [Bacteria](https://www.ncbi.nlm.nih.gov/Taxonomy/Browser/wwwtax.cgi?mode=Undef&id=2&lvl=3&keep=1&srchmode=1&unlock) |
| 55* | 100% | *0* |  | [GU911381.1](https://www.ncbi.nlm.nih.gov/nucleotide/GU911381.1?report=genbank&log$=nucltop&blast_rank=1&RID=38AYR9AW013) |  |  |  |  |  | [Bacteria](https://www.ncbi.nlm.nih.gov/Taxonomy/Browser/wwwtax.cgi?mode=Undef&id=2&lvl=3&keep=1&srchmode=1&unlock) |
| 56* | 100% | *0* |  | [GU911382.1](https://www.ncbi.nlm.nih.gov/nucleotide/GU911382.1?report=genbank&log$=nucltop&blast_rank=1&RID=38BKAMGB016) |  |  |  | [Chlorobi](https://www.ncbi.nlm.nih.gov/Taxonomy/Browser/wwwtax.cgi?mode=Undef&id=68336&lvl=3&keep=1&srchmode=1&unlock) | [Bacteroidetes](https://www.ncbi.nlm.nih.gov/Taxonomy/Browser/wwwtax.cgi?mode=Undef&id=68336&lvl=3&keep=1&srchmode=1&unlock) | [Bacteria](https://www.ncbi.nlm.nih.gov/Taxonomy/Browser/wwwtax.cgi?mode=Undef&id=2&lvl=3&keep=1&srchmode=1&unlock) |
| 57* | 100% | *0* |  | [GU911383.1](https://www.ncbi.nlm.nih.gov/nucleotide/GU911383.1?report=genbank&log$=nucltop&blast_rank=1&RID=38BWGG5401R) | [*Verrucomicrobia*](https://www.ncbi.nlm.nih.gov/Taxonomy/Browser/wwwtax.cgi?mode=Undef&id=74201&lvl=3&keep=1&srchmode=1&unlock) |  |  |  | [PVC group](https://www.ncbi.nlm.nih.gov/Taxonomy/Browser/wwwtax.cgi?mode=Undef&id=1783257&lvl=3&keep=1&srchmode=1&unlock) | [Bacteria](https://www.ncbi.nlm.nih.gov/Taxonomy/Browser/wwwtax.cgi?mode=Undef&id=2&lvl=3&keep=1&srchmode=1&unlock) |
| 58* | 100% | *0* |  | [GU911387.1](https://www.ncbi.nlm.nih.gov/nucleotide/GU911387.1?report=genbank&log$=nucltop&blast_rank=1&RID=38C4K267013) |  |  |  |  | [Acidobacteria](https://www.ncbi.nlm.nih.gov/Taxonomy/Browser/wwwtax.cgi?mode=Undef&id=57723&lvl=3&keep=1&srchmode=1&unlock) | [Bacteria](https://www.ncbi.nlm.nih.gov/Taxonomy/Browser/wwwtax.cgi?mode=Undef&id=2&lvl=3&keep=1&srchmode=1&unlock) |
| 59* | 100% | *0* |  | [GU911390.1](https://www.ncbi.nlm.nih.gov/nucleotide/GU911390.1?report=genbank&log$=nucltop&blast_rank=1&RID=38CY15PN01R) |  | [Anaerolineae](https://www.ncbi.nlm.nih.gov/Taxonomy/Browser/wwwtax.cgi?mode=Undef&id=292625&lvl=3&keep=1&srchmode=1&unlock) |  | [Chloroflexi](https://www.ncbi.nlm.nih.gov/Taxonomy/Browser/wwwtax.cgi?mode=Undef&id=200795&lvl=3&keep=1&srchmode=1&unlock) | [Terrabacteria](https://www.ncbi.nlm.nih.gov/Taxonomy/Browser/wwwtax.cgi?mode=Undef&id=1783272&lvl=3&keep=1&srchmode=1&unlock) | [Bacteria](https://www.ncbi.nlm.nih.gov/Taxonomy/Browser/wwwtax.cgi?mode=Undef&id=2&lvl=3&keep=1&srchmode=1&unlock) |
| 60* | 100% | *0* |  | [GU911392.1](https://www.ncbi.nlm.nih.gov/nucleotide/GU911392.1?report=genbank&log$=nucltop&blast_rank=1&RID=38DM0MTG016) |  |  |  |  | [Acidobacteria](https://www.ncbi.nlm.nih.gov/Taxonomy/Browser/wwwtax.cgi?mode=Undef&id=57723&lvl=3&keep=1&srchmode=1&unlock) | [Bacteria](https://www.ncbi.nlm.nih.gov/Taxonomy/Browser/wwwtax.cgi?mode=Undef&id=2&lvl=3&keep=1&srchmode=1&unlock) |
| 61* | 100% | *0* |  | [GU911393.1](https://www.ncbi.nlm.nih.gov/nucleotide/GU911393.1?report=genbank&log$=nucltop&blast_rank=1&RID=38DUE5Z901R) |  |  |  |  | [Acidobacteria](https://www.ncbi.nlm.nih.gov/Taxonomy/Browser/wwwtax.cgi?mode=Undef&id=57723&lvl=3&keep=1&srchmode=1&unlock) | [Bacteria](https://www.ncbi.nlm.nih.gov/Taxonomy/Browser/wwwtax.cgi?mode=Undef&id=2&lvl=3&keep=1&srchmode=1&unlock) |
| 62 | 99.47% | *0* | ***Moraxella osloensis*** | [MT225646.1](https://www.ncbi.nlm.nih.gov/nucleotide/MT225646.1?report=genbank&log$=nucltop&blast_rank=4&RID=38E3AWGV013) | [*Moraxella*](https://www.ncbi.nlm.nih.gov/Taxonomy/Browser/wwwtax.cgi?mode=Undef&id=475&lvl=3&keep=1&srchmode=1&unlock) | [Moraxellaceae](https://www.ncbi.nlm.nih.gov/Taxonomy/Browser/wwwtax.cgi?mode=Undef&id=468&lvl=3&keep=1&srchmode=1&unlock) | [Pseudomonadales](https://www.ncbi.nlm.nih.gov/Taxonomy/Browser/wwwtax.cgi?mode=Undef&id=72274&lvl=3&keep=1&srchmode=1&unlock) | [Gammaproteobacteria](https://www.ncbi.nlm.nih.gov/Taxonomy/Browser/wwwtax.cgi?mode=Undef&id=1236&lvl=3&keep=1&srchmode=1&unlock) | [Proteobacteria](https://www.ncbi.nlm.nih.gov/Taxonomy/Browser/wwwtax.cgi?mode=Undef&id=1224&lvl=3&keep=1&srchmode=1&unlock) | [Bacteria](https://www.ncbi.nlm.nih.gov/Taxonomy/Browser/wwwtax.cgi?mode=Undef&id=2&lvl=3&keep=1&srchmode=1&unlock) |
| 63 | 99.75 | *0* | ***Moraxella osloensis*** | [MT225646.1](https://www.ncbi.nlm.nih.gov/nucleotide/MT225646.1?report=genbank&log$=nucltop&blast_rank=2&RID=38ENNZPP01R) | [*Moraxella*](https://www.ncbi.nlm.nih.gov/Taxonomy/Browser/wwwtax.cgi?mode=Undef&id=475&lvl=3&keep=1&srchmode=1&unlock) | [Moraxellaceae](https://www.ncbi.nlm.nih.gov/Taxonomy/Browser/wwwtax.cgi?mode=Undef&id=468&lvl=3&keep=1&srchmode=1&unlock) | [Pseudomonadales](https://www.ncbi.nlm.nih.gov/Taxonomy/Browser/wwwtax.cgi?mode=Undef&id=72274&lvl=3&keep=1&srchmode=1&unlock) | [Gammaproteobacteria](https://www.ncbi.nlm.nih.gov/Taxonomy/Browser/wwwtax.cgi?mode=Undef&id=1236&lvl=3&keep=1&srchmode=1&unlock) | [Proteobacteria](https://www.ncbi.nlm.nih.gov/Taxonomy/Browser/wwwtax.cgi?mode=Undef&id=1224&lvl=3&keep=1&srchmode=1&unlock) | [Bacteria](https://www.ncbi.nlm.nih.gov/Taxonomy/Browser/wwwtax.cgi?mode=Undef&id=2&lvl=3&keep=1&srchmode=1&unlock) |
| 64 | *99%* | *0* | ***Moraxella osloensis*** | [MT225646.1](https://www.ncbi.nlm.nih.gov/nucleotide/MT225646.1?report=genbank&log$=nucltop&blast_rank=3&RID=38EUXU92016) | [*Moraxella*](https://www.ncbi.nlm.nih.gov/Taxonomy/Browser/wwwtax.cgi?mode=Undef&id=475&lvl=3&keep=1&srchmode=1&unlock) | [Moraxellaceae](https://www.ncbi.nlm.nih.gov/Taxonomy/Browser/wwwtax.cgi?mode=Undef&id=468&lvl=3&keep=1&srchmode=1&unlock) | [Pseudomonadales](https://www.ncbi.nlm.nih.gov/Taxonomy/Browser/wwwtax.cgi?mode=Undef&id=72274&lvl=3&keep=1&srchmode=1&unlock) | [Gammaproteobacteria](https://www.ncbi.nlm.nih.gov/Taxonomy/Browser/wwwtax.cgi?mode=Undef&id=1236&lvl=3&keep=1&srchmode=1&unlock) | [Proteobacteria](https://www.ncbi.nlm.nih.gov/Taxonomy/Browser/wwwtax.cgi?mode=Undef&id=1224&lvl=3&keep=1&srchmode=1&unlock) | [Bacteria](https://www.ncbi.nlm.nih.gov/Taxonomy/Browser/wwwtax.cgi?mode=Undef&id=2&lvl=3&keep=1&srchmode=1&unlock) |
| 65 | 99.83 | *0* | ***Moraxella osloensis*** | [MT225646.1](https://www.ncbi.nlm.nih.gov/nucleotide/MT225646.1?report=genbank&log$=nucltop&blast_rank=2&RID=38F1P4F1016) | [*Moraxella*](https://www.ncbi.nlm.nih.gov/Taxonomy/Browser/wwwtax.cgi?mode=Undef&id=475&lvl=3&keep=1&srchmode=1&unlock) | [Moraxellaceae](https://www.ncbi.nlm.nih.gov/Taxonomy/Browser/wwwtax.cgi?mode=Undef&id=468&lvl=3&keep=1&srchmode=1&unlock) | [Pseudomonadales](https://www.ncbi.nlm.nih.gov/Taxonomy/Browser/wwwtax.cgi?mode=Undef&id=72274&lvl=3&keep=1&srchmode=1&unlock) | [Gammaproteobacteria](https://www.ncbi.nlm.nih.gov/Taxonomy/Browser/wwwtax.cgi?mode=Undef&id=1236&lvl=3&keep=1&srchmode=1&unlock) | [Proteobacteria](https://www.ncbi.nlm.nih.gov/Taxonomy/Browser/wwwtax.cgi?mode=Undef&id=1224&lvl=3&keep=1&srchmode=1&unlock) | [Bacteria](https://www.ncbi.nlm.nih.gov/Taxonomy/Browser/wwwtax.cgi?mode=Undef&id=2&lvl=3&keep=1&srchmode=1&unlock) |
| 66 | 99.66% | *0* | ***Moraxella osloensis*** | [MT225646.1](https://www.ncbi.nlm.nih.gov/nucleotide/MT225646.1?report=genbank&log$=nucltop&blast_rank=2&RID=38FAMS9K01R) | [*Moraxella*](https://www.ncbi.nlm.nih.gov/Taxonomy/Browser/wwwtax.cgi?mode=Undef&id=475&lvl=3&keep=1&srchmode=1&unlock) | [Moraxellaceae](https://www.ncbi.nlm.nih.gov/Taxonomy/Browser/wwwtax.cgi?mode=Undef&id=468&lvl=3&keep=1&srchmode=1&unlock) | [Pseudomonadales](https://www.ncbi.nlm.nih.gov/Taxonomy/Browser/wwwtax.cgi?mode=Undef&id=72274&lvl=3&keep=1&srchmode=1&unlock) | [Gammaproteobacteria](https://www.ncbi.nlm.nih.gov/Taxonomy/Browser/wwwtax.cgi?mode=Undef&id=1236&lvl=3&keep=1&srchmode=1&unlock) | [Proteobacteria](https://www.ncbi.nlm.nih.gov/Taxonomy/Browser/wwwtax.cgi?mode=Undef&id=1224&lvl=3&keep=1&srchmode=1&unlock) | [Bacteria](https://www.ncbi.nlm.nih.gov/Taxonomy/Browser/wwwtax.cgi?mode=Undef&id=2&lvl=3&keep=1&srchmode=1&unlock) |
| 67 | 99.66 | *0* | ***Moraxella osloensis*** | [MT225646.1](https://www.ncbi.nlm.nih.gov/nucleotide/MT225646.1?report=genbank&log$=nucltop&blast_rank=2&RID=38FTJYHX01R) | [*Moraxella*](https://www.ncbi.nlm.nih.gov/Taxonomy/Browser/wwwtax.cgi?mode=Undef&id=475&lvl=3&keep=1&srchmode=1&unlock) | [Moraxellaceae](https://www.ncbi.nlm.nih.gov/Taxonomy/Browser/wwwtax.cgi?mode=Undef&id=468&lvl=3&keep=1&srchmode=1&unlock) | [Pseudomonadales](https://www.ncbi.nlm.nih.gov/Taxonomy/Browser/wwwtax.cgi?mode=Undef&id=72274&lvl=3&keep=1&srchmode=1&unlock) | [Gammaproteobacteria](https://www.ncbi.nlm.nih.gov/Taxonomy/Browser/wwwtax.cgi?mode=Undef&id=1236&lvl=3&keep=1&srchmode=1&unlock) | [Proteobacteria](https://www.ncbi.nlm.nih.gov/Taxonomy/Browser/wwwtax.cgi?mode=Undef&id=1224&lvl=3&keep=1&srchmode=1&unlock) | [Bacteria](https://www.ncbi.nlm.nih.gov/Taxonomy/Browser/wwwtax.cgi?mode=Undef&id=2&lvl=3&keep=1&srchmode=1&unlock) |
| 68 | 97% | *0* | ***Labilithrix luteola*** | [CP012333.1](https://www.ncbi.nlm.nih.gov/nucleotide/CP012333.1?report=genbank&log$=nucltop&blast_rank=46&RID=38FZP4XG016) | [*Labilithrix*](https://www.ncbi.nlm.nih.gov/Taxonomy/Browser/wwwtax.cgi?mode=Undef&id=1524217&lvl=3&keep=1&srchmode=1&unlock) | [Labilitrichaceae](https://www.ncbi.nlm.nih.gov/Taxonomy/Browser/wwwtax.cgi?mode=Undef&id=1524216&lvl=3&keep=1&srchmode=1&unlock) | [Myxococcales](https://www.ncbi.nlm.nih.gov/Taxonomy/Browser/wwwtax.cgi?mode=Undef&id=29&lvl=3&keep=1&srchmode=1&unlock) | [Deltaproteobacteria](https://www.ncbi.nlm.nih.gov/Taxonomy/Browser/wwwtax.cgi?mode=Undef&id=28221&lvl=3&keep=1&srchmode=1&unlock) | [Proteobacteria](https://www.ncbi.nlm.nih.gov/Taxonomy/Browser/wwwtax.cgi?mode=Undef&id=1224&lvl=3&keep=1&srchmode=1&unlock) | [Bacteria](https://www.ncbi.nlm.nih.gov/Taxonomy/Browser/wwwtax.cgi?mode=Undef&id=2&lvl=3&keep=1&srchmode=1&unlock) |
| 69 | 89.52% | *0* | ***Vulgatibacter incomptus*** | [CP012332.1](https://www.ncbi.nlm.nih.gov/nucleotide/CP012332.1?report=genbank&log$=nucltop&blast_rank=89&RID=38GCJ5AS016) | [*Vulgatibacter*](https://www.ncbi.nlm.nih.gov/Taxonomy/Browser/wwwtax.cgi?mode=Undef&id=1524214&lvl=3&keep=1&srchmode=1&unlock) | [Vulgatibacteraceae](https://www.ncbi.nlm.nih.gov/Taxonomy/Browser/wwwtax.cgi?mode=Undef&id=1524213&lvl=3&keep=1&srchmode=1&unlock) | [Myxococcales](https://www.ncbi.nlm.nih.gov/Taxonomy/Browser/wwwtax.cgi?mode=Undef&id=29&lvl=3&keep=1&srchmode=1&unlock) | [Deltaproteobacteria](https://www.ncbi.nlm.nih.gov/Taxonomy/Browser/wwwtax.cgi?mode=Undef&id=28221&lvl=3&keep=1&srchmode=1&unlock) | [Proteobacteria](https://www.ncbi.nlm.nih.gov/Taxonomy/Browser/wwwtax.cgi?mode=Undef&id=1224&lvl=3&keep=1&srchmode=1&unlock) | [Bacteria](https://www.ncbi.nlm.nih.gov/Taxonomy/Browser/wwwtax.cgi?mode=Undef&id=2&lvl=3&keep=1&srchmode=1&unlock) |
| 70* | 100% | *0* |  | [GU911403.1](https://www.ncbi.nlm.nih.gov/nucleotide/GU911403.1?report=genbank&log$=nucltop&blast_rank=1&RID=38GUG08F01R) |  |  | [Planctomycetales](https://www.ncbi.nlm.nih.gov/Taxonomy/Browser/wwwtax.cgi?mode=Undef&id=112&lvl=3&keep=1&srchmode=1&unlock) | [Planctomycetia](https://www.ncbi.nlm.nih.gov/Taxonomy/Browser/wwwtax.cgi?mode=Undef&id=203683&lvl=3&keep=1&srchmode=1&unlock) | [Planctomycetes](https://www.ncbi.nlm.nih.gov/Taxonomy/Browser/wwwtax.cgi?mode=Undef&id=203682&lvl=3&keep=1&srchmode=1&unlock) | [Bacteria](https://www.ncbi.nlm.nih.gov/Taxonomy/Browser/wwwtax.cgi?mode=Undef&id=2&lvl=3&keep=1&srchmode=1&unlock) |
| 71* | 100% | *0* |  | [GU911405.1](https://www.ncbi.nlm.nih.gov/nucleotide/GU911405.1?report=genbank&log$=nucltop&blast_rank=1&RID=38H4W3CE013) |  |  |  | [Actinomycetia](https://www.ncbi.nlm.nih.gov/Taxonomy/Browser/wwwtax.cgi?mode=Undef&id=1760&lvl=3&keep=1&srchmode=1&unlock) | [Actinobacteria](https://www.ncbi.nlm.nih.gov/Taxonomy/Browser/wwwtax.cgi?mode=Undef&id=201174&lvl=3&keep=1&srchmode=1&unlock) | [Bacteria](https://www.ncbi.nlm.nih.gov/Taxonomy/Browser/wwwtax.cgi?mode=Undef&id=2&lvl=3&keep=1&srchmode=1&unlock) |
| 72 | 95.9% | *0* | ***Propionibacterium sp*** | [KF906602.1](https://www.ncbi.nlm.nih.gov/nucleotide/KF906602.1?report=genbank&log$=nucltop&blast_rank=12&RID=38HCAFPK01R) | [*Propionibacterium*](https://www.ncbi.nlm.nih.gov/Taxonomy/Browser/wwwtax.cgi?mode=Undef&id=1743&lvl=3&keep=1&srchmode=1&unlock) | [Propionibacteriaceae](https://www.ncbi.nlm.nih.gov/Taxonomy/Browser/wwwtax.cgi?mode=Undef&id=31957&lvl=3&keep=1&srchmode=1&unlock) | [Propionibacteriales](https://www.ncbi.nlm.nih.gov/Taxonomy/Browser/wwwtax.cgi?mode=Undef&id=85009&lvl=3&keep=1&srchmode=1&unlock) | [Actinomycetia](https://www.ncbi.nlm.nih.gov/Taxonomy/Browser/wwwtax.cgi?mode=Undef&id=1760&lvl=3&keep=1&srchmode=1&unlock) | [Actinobacteria](https://www.ncbi.nlm.nih.gov/Taxonomy/Browser/wwwtax.cgi?mode=Undef&id=201174&lvl=3&keep=1&srchmode=1&unlock) | [Bacteria](https://www.ncbi.nlm.nih.gov/Taxonomy/Browser/wwwtax.cgi?mode=Undef&id=2&lvl=3&keep=1&srchmode=1&unlock) |
| 75 | 100% | *0* | *Methanosaeta sp* | [GU911410.1](https://www.ncbi.nlm.nih.gov/nucleotide/GU911410.1?report=genbank&log$=nucltop&blast_rank=1&RID=38J3VUGV013) |  |  |  |  |  | [Archaea](https://www.ncbi.nlm.nih.gov/Taxonomy/Browser/wwwtax.cgi?mode=Undef&id=2157&lvl=3&keep=1&srchmode=1&unlock) |
| 77 | 97.6% | *0* | ***Methanoregula formicica*** | [NR_102441.1](https://www.ncbi.nlm.nih.gov/nucleotide/NR_102441.1?report=genbank&log$=nucltop&blast_rank=49&RID=38JD3J2601R) |  |  |  |  |  | **Archaea** |
| 78 | 99% | *0* | ***Paenibacillus sp.*** | [MT449014.1](https://www.ncbi.nlm.nih.gov/nucleotide/MT449014.1?report=genbank&log$=nucltop&blast_rank=1&RID=38JJR1HU016) | [*Paenibacillus*](https://www.ncbi.nlm.nih.gov/Taxonomy/Browser/wwwtax.cgi?mode=Undef&id=44249&lvl=3&keep=1&srchmode=1&unlock) | [Paenibacillaceae](https://www.ncbi.nlm.nih.gov/Taxonomy/Browser/wwwtax.cgi?mode=Undef&id=186822&lvl=3&keep=1&srchmode=1&unlock) | [Bacillales](https://www.ncbi.nlm.nih.gov/Taxonomy/Browser/wwwtax.cgi?mode=Undef&id=1385&lvl=3&keep=1&srchmode=1&unlock) | [Bacilli](https://www.ncbi.nlm.nih.gov/Taxonomy/Browser/wwwtax.cgi?mode=Undef&id=91061&lvl=3&keep=1&srchmode=1&unlock) | [Firmicutes](https://www.ncbi.nlm.nih.gov/Taxonomy/Browser/wwwtax.cgi?mode=Undef&id=1239&lvl=3&keep=1&srchmode=1&unlock) | [Bacteria](https://www.ncbi.nlm.nih.gov/Taxonomy/Browser/wwwtax.cgi?mode=Undef&id=2&lvl=3&keep=1&srchmode=1&unlock) |
| 79 | 99.84 | *0* | ***Acinetobacter rhizosphaerae*** | [MT386200.1](https://www.ncbi.nlm.nih.gov/nucleotide/MT386200.1?report=genbank&log$=nucltop&blast_rank=3&RID=38JTPNTX01R) | [*Acinetobacter*](https://www.ncbi.nlm.nih.gov/Taxonomy/Browser/wwwtax.cgi?mode=Undef&id=469&lvl=3&keep=1&srchmode=1&unlock) | [Moraxellaceae](https://www.ncbi.nlm.nih.gov/Taxonomy/Browser/wwwtax.cgi?mode=Undef&id=468&lvl=3&keep=1&srchmode=1&unlock) | [Pseudomonadales](https://www.ncbi.nlm.nih.gov/Taxonomy/Browser/wwwtax.cgi?mode=Undef&id=72274&lvl=3&keep=1&srchmode=1&unlock) | [Gammaproteobacteria](https://www.ncbi.nlm.nih.gov/Taxonomy/Browser/wwwtax.cgi?mode=Undef&id=1236&lvl=3&keep=1&srchmode=1&unlock) | [Proteobacteria](https://www.ncbi.nlm.nih.gov/Taxonomy/Browser/wwwtax.cgi?mode=Undef&id=1224&lvl=3&keep=1&srchmode=1&unlock) | [Bacteria](https://www.ncbi.nlm.nih.gov/Taxonomy/Browser/wwwtax.cgi?mode=Undef&id=2&lvl=3&keep=1&srchmode=1&unlock) |
| 80 | 99.85 | *0* | ***Acinetobacter pittii*** | [CP049806.1](https://www.ncbi.nlm.nih.gov/nucleotide/CP049806.1?report=genbank&log$=nucltop&blast_rank=2&RID=38K6R5ZU013) | ***Acinetobacte*** | [Moraxellaceae](https://www.ncbi.nlm.nih.gov/Taxonomy/Browser/wwwtax.cgi?mode=Undef&id=468&lvl=3&keep=1&srchmode=1&unlock) | [Pseudomonadales](https://www.ncbi.nlm.nih.gov/Taxonomy/Browser/wwwtax.cgi?mode=Undef&id=72274&lvl=3&keep=1&srchmode=1&unlock) | [Gammaproteobacteria](https://www.ncbi.nlm.nih.gov/Taxonomy/Browser/wwwtax.cgi?mode=Undef&id=1236&lvl=3&keep=1&srchmode=1&unlock) | [Proteobacteria](https://www.ncbi.nlm.nih.gov/Taxonomy/Browser/wwwtax.cgi?mode=Undef&id=1224&lvl=3&keep=1&srchmode=1&unlock) | [Bacteria](https://www.ncbi.nlm.nih.gov/Taxonomy/Browser/wwwtax.cgi?mode=Undef&id=2&lvl=3&keep=1&srchmode=1&unlock) |
| 81 | 100% | *0* | ***Acinetobacter pittii*** | [CP049806.1](https://www.ncbi.nlm.nih.gov/nucleotide/CP049806.1?report=genbank&log$=nucltop&blast_rank=1&RID=38KHWGMU016) | ***Acinetobacte*** | [Moraxellaceae](https://www.ncbi.nlm.nih.gov/Taxonomy/Browser/wwwtax.cgi?mode=Undef&id=468&lvl=3&keep=1&srchmode=1&unlock) | [Pseudomonadales](https://www.ncbi.nlm.nih.gov/Taxonomy/Browser/wwwtax.cgi?mode=Undef&id=72274&lvl=3&keep=1&srchmode=1&unlock) | [Gammaproteobacteria](https://www.ncbi.nlm.nih.gov/Taxonomy/Browser/wwwtax.cgi?mode=Undef&id=1236&lvl=3&keep=1&srchmode=1&unlock) | [Proteobacteria](https://www.ncbi.nlm.nih.gov/Taxonomy/Browser/wwwtax.cgi?mode=Undef&id=1224&lvl=3&keep=1&srchmode=1&unlock) | [Bacteria](https://www.ncbi.nlm.nih.gov/Taxonomy/Browser/wwwtax.cgi?mode=Undef&id=2&lvl=3&keep=1&srchmode=1&unlock) |
| 82 | 100% | *0* | ***Pseudomonas alcaligenes*** | [KP644236.1](https://www.ncbi.nlm.nih.gov/nucleotide/KP644236.1?report=genbank&log$=nucltop&blast_rank=4&RID=38KPXU5Z016) | [*Pseudomonas*](https://www.ncbi.nlm.nih.gov/Taxonomy/Browser/wwwtax.cgi?mode=Undef&id=286&lvl=3&keep=1&srchmode=1&unlock) | [Pseudomonadaceae](https://www.ncbi.nlm.nih.gov/Taxonomy/Browser/wwwtax.cgi?mode=Undef&id=135621&lvl=3&keep=1&srchmode=1&unlock) | [Pseudomonadales](https://www.ncbi.nlm.nih.gov/Taxonomy/Browser/wwwtax.cgi?mode=Undef&id=72274&lvl=3&keep=1&srchmode=1&unlock) | [Gammaproteobacteria](https://www.ncbi.nlm.nih.gov/Taxonomy/Browser/wwwtax.cgi?mode=Undef&id=1236&lvl=3&keep=1&srchmode=1&unlock) | [Proteobacteria](https://www.ncbi.nlm.nih.gov/Taxonomy/Browser/wwwtax.cgi?mode=Undef&id=1224&lvl=3&keep=1&srchmode=1&unlock) | [Bacteria](https://www.ncbi.nlm.nih.gov/Taxonomy/Browser/wwwtax.cgi?mode=Undef&id=2&lvl=3&keep=1&srchmode=1&unlock) |
| 83 | 98.18% | *0* | *Nevskia sp.* | [MH698687.1](https://www.ncbi.nlm.nih.gov/nucleotide/MH698687.1?report=genbank&log$=nucltop&blast_rank=14&RID=38KZPDBV01R) | [*Nevskia*](https://www.ncbi.nlm.nih.gov/Taxonomy/Browser/wwwtax.cgi?mode=Undef&id=64001&lvl=3&keep=1&srchmode=1&unlock) | [Sinobacteraceae](https://www.ncbi.nlm.nih.gov/Taxonomy/Browser/wwwtax.cgi?mode=Undef&id=568386&lvl=3&keep=1&srchmode=1&unlock) | [Nevskiales](https://www.ncbi.nlm.nih.gov/Taxonomy/Browser/wwwtax.cgi?mode=Undef&id=1775403&lvl=3&keep=1&srchmode=1&unlock) | [Gammaproteobacteria](https://www.ncbi.nlm.nih.gov/Taxonomy/Browser/wwwtax.cgi?mode=Undef&id=1236&lvl=3&keep=1&srchmode=1&unlock) | [Proteobacteria](https://www.ncbi.nlm.nih.gov/Taxonomy/Browser/wwwtax.cgi?mode=Undef&id=1224&lvl=3&keep=1&srchmode=1&unlock) | [Bacteria](https://www.ncbi.nlm.nih.gov/Taxonomy/Browser/wwwtax.cgi?mode=Undef&id=2&lvl=3&keep=1&srchmode=1&unlock) |
| 84 | 98.85% | *0* | ***Caulobacter sp*** | [AB979861.1](https://www.ncbi.nlm.nih.gov/nucleotide/AB979861.1?report=genbank&log$=nucltop&blast_rank=40&RID=38M894NK016) | [*Caulobacter*](https://www.ncbi.nlm.nih.gov/Taxonomy/Browser/wwwtax.cgi?mode=Undef&id=75&lvl=3&keep=1&srchmode=1&unlock) | [Caulobacteraceae](https://www.ncbi.nlm.nih.gov/Taxonomy/Browser/wwwtax.cgi?mode=Undef&id=76892&lvl=3&keep=1&srchmode=1&unlock) | [Caulobacterales](https://www.ncbi.nlm.nih.gov/Taxonomy/Browser/wwwtax.cgi?mode=Undef&id=204458&lvl=3&keep=1&srchmode=1&unlock) | [Alphaproteobacteria](https://www.ncbi.nlm.nih.gov/Taxonomy/Browser/wwwtax.cgi?mode=Undef&id=28211&lvl=3&keep=1&srchmode=1&unlock) | [Proteobacteria](https://www.ncbi.nlm.nih.gov/Taxonomy/Browser/wwwtax.cgi?mode=Undef&id=1224&lvl=3&keep=1&srchmode=1&unlock) | [Bacteria](https://www.ncbi.nlm.nih.gov/Taxonomy/Browser/wwwtax.cgi?mode=Undef&id=2&lvl=3&keep=1&srchmode=1&unlock) |
| 85 | 99.29% | *0* | ***Caulobacter sp*** | [KM555262.1](https://www.ncbi.nlm.nih.gov/nucleotide/KM555262.1?report=genbank&log$=nucltop&blast_rank=93&RID=38MGU33B016) | [*Caulobacter*](https://www.ncbi.nlm.nih.gov/Taxonomy/Browser/wwwtax.cgi?mode=Undef&id=75&lvl=3&keep=1&srchmode=1&unlock) | [Caulobacteraceae](https://www.ncbi.nlm.nih.gov/Taxonomy/Browser/wwwtax.cgi?mode=Undef&id=76892&lvl=3&keep=1&srchmode=1&unlock) | [Caulobacterales](https://www.ncbi.nlm.nih.gov/Taxonomy/Browser/wwwtax.cgi?mode=Undef&id=204458&lvl=3&keep=1&srchmode=1&unlock) | [Alphaproteobacteria](https://www.ncbi.nlm.nih.gov/Taxonomy/Browser/wwwtax.cgi?mode=Undef&id=28211&lvl=3&keep=1&srchmode=1&unlock) | [Proteobacteria](https://www.ncbi.nlm.nih.gov/Taxonomy/Browser/wwwtax.cgi?mode=Undef&id=1224&lvl=3&keep=1&srchmode=1&unlock) | [Bacteria](https://www.ncbi.nlm.nih.gov/Taxonomy/Browser/wwwtax.cgi?mode=Undef&id=2&lvl=3&keep=1&srchmode=1&unlock) |
| 86 | 99.44% | *0* | *Nevskia soli* | [AB682417.1](https://www.ncbi.nlm.nih.gov/nucleotide/AB682417.1?report=genbank&log$=nucltop&blast_rank=3&RID=3AFSN6ZC01R) | [*Nevskia*](https://www.ncbi.nlm.nih.gov/Taxonomy/Browser/wwwtax.cgi?mode=Undef&id=64001&lvl=3&keep=1&srchmode=1&unlock) | [Sinobacteraceae](https://www.ncbi.nlm.nih.gov/Taxonomy/Browser/wwwtax.cgi?mode=Undef&id=568386&lvl=3&keep=1&srchmode=1&unlock) | [Nevskiales](https://www.ncbi.nlm.nih.gov/Taxonomy/Browser/wwwtax.cgi?mode=Undef&id=1775403&lvl=3&keep=1&srchmode=1&unlock) | [Gammaproteobacteria](https://www.ncbi.nlm.nih.gov/Taxonomy/Browser/wwwtax.cgi?mode=Undef&id=1236&lvl=3&keep=1&srchmode=1&unlock) | [Proteobacteria](https://www.ncbi.nlm.nih.gov/Taxonomy/Browser/wwwtax.cgi?mode=Undef&id=1224&lvl=3&keep=1&srchmode=1&unlock) | [Bacteria](https://www.ncbi.nlm.nih.gov/Taxonomy/Browser/wwwtax.cgi?mode=Undef&id=2&lvl=3&keep=1&srchmode=1&unlock) |
| 87 | 100% | *0* | ***Streptomyces fulvissimus*** | [CP054926.1](https://www.ncbi.nlm.nih.gov/nucleotide/CP054926.1?report=genbank&log$=nucltop&blast_rank=2&RID=3AG5AHS301R) | [*Streptomyces*](https://www.ncbi.nlm.nih.gov/Taxonomy/Browser/wwwtax.cgi?mode=Undef&id=1883&lvl=3&keep=1&srchmode=1&unlock) | [Streptomycetaceae](https://www.ncbi.nlm.nih.gov/Taxonomy/Browser/wwwtax.cgi?mode=Undef&id=2062&lvl=3&keep=1&srchmode=1&unlock) | [Streptomycetales](https://www.ncbi.nlm.nih.gov/Taxonomy/Browser/wwwtax.cgi?mode=Undef&id=85011&lvl=3&keep=1&srchmode=1&unlock) | [Actinomycetia](https://www.ncbi.nlm.nih.gov/Taxonomy/Browser/wwwtax.cgi?mode=Undef&id=1760&lvl=3&keep=1&srchmode=1&unlock) | [Actinobacteria](https://www.ncbi.nlm.nih.gov/Taxonomy/Browser/wwwtax.cgi?mode=Undef&id=201174&lvl=3&keep=1&srchmode=1&unlock) | [Bacteria](https://www.ncbi.nlm.nih.gov/Taxonomy/Browser/wwwtax.cgi?mode=Undef&id=2&lvl=3&keep=1&srchmode=1&unlock) |
| 88 | 99% | *0* | ***Caulobacter sp*** | [AB979861.1](https://www.ncbi.nlm.nih.gov/nucleotide/AB979861.1?report=genbank&log$=nucltop&blast_rank=28&RID=3AGC6H6F01R) | [*Caulobacter*](https://www.ncbi.nlm.nih.gov/Taxonomy/Browser/wwwtax.cgi?mode=Undef&id=75&lvl=3&keep=1&srchmode=1&unlock) | [Caulobacteraceae](https://www.ncbi.nlm.nih.gov/Taxonomy/Browser/wwwtax.cgi?mode=Undef&id=76892&lvl=3&keep=1&srchmode=1&unlock) | [Caulobacterales](https://www.ncbi.nlm.nih.gov/Taxonomy/Browser/wwwtax.cgi?mode=Undef&id=204458&lvl=3&keep=1&srchmode=1&unlock) | [Alphaproteobacteria](https://www.ncbi.nlm.nih.gov/Taxonomy/Browser/wwwtax.cgi?mode=Undef&id=28211&lvl=3&keep=1&srchmode=1&unlock) | [Proteobacteria](https://www.ncbi.nlm.nih.gov/Taxonomy/Browser/wwwtax.cgi?mode=Undef&id=1224&lvl=3&keep=1&srchmode=1&unlock) | [Bacteria](https://www.ncbi.nlm.nih.gov/Taxonomy/Browser/wwwtax.cgi?mode=Undef&id=2&lvl=3&keep=1&srchmode=1&unlock) |
| 89 | 99% | *0* | ***Aeromonas veronii*** | [MT384380.1](https://www.ncbi.nlm.nih.gov/nucleotide/MT384380.1?report=genbank&log$=nucltop&blast_rank=10&RID=3AGP055F01R) | [*Aeromonas*](https://www.ncbi.nlm.nih.gov/Taxonomy/Browser/wwwtax.cgi?mode=Undef&id=642&lvl=3&keep=1&srchmode=1&unlock) | [Aeromonadaceae](https://www.ncbi.nlm.nih.gov/Taxonomy/Browser/wwwtax.cgi?mode=Undef&id=84642&lvl=3&keep=1&srchmode=1&unlock) | [Aeromonadales](https://www.ncbi.nlm.nih.gov/Taxonomy/Browser/wwwtax.cgi?mode=Undef&id=135624&lvl=3&keep=1&srchmode=1&unlock) | [Gammaproteobacteria](https://www.ncbi.nlm.nih.gov/Taxonomy/Browser/wwwtax.cgi?mode=Undef&id=1236&lvl=3&keep=1&srchmode=1&unlock) | [Proteobacteria](https://www.ncbi.nlm.nih.gov/Taxonomy/Browser/wwwtax.cgi?mode=Undef&id=1224&lvl=3&keep=1&srchmode=1&unlock) | [Bacteria](https://www.ncbi.nlm.nih.gov/Taxonomy/Browser/wwwtax.cgi?mode=Undef&id=2&lvl=3&keep=1&srchmode=1&unlock) |
| 90 | 99.78% | *0* | ***Staphylococcus epidermidis*** | [MT605363.1](https://www.ncbi.nlm.nih.gov/nucleotide/MT605363.1?report=genbank&log$=nucltop&blast_rank=2&RID=3AH8U9C5013) | [*Staphylococcus*](https://www.ncbi.nlm.nih.gov/Taxonomy/Browser/wwwtax.cgi?mode=Undef&id=1279&lvl=3&keep=1&srchmode=1&unlock) | [Staphylococcaceae](https://www.ncbi.nlm.nih.gov/Taxonomy/Browser/wwwtax.cgi?mode=Undef&id=90964&lvl=3&keep=1&srchmode=1&unlock) | [Bacillales](https://www.ncbi.nlm.nih.gov/Taxonomy/Browser/wwwtax.cgi?mode=Undef&id=1385&lvl=3&keep=1&srchmode=1&unlock) | [Bacilli](https://www.ncbi.nlm.nih.gov/Taxonomy/Browser/wwwtax.cgi?mode=Undef&id=91061&lvl=3&keep=1&srchmode=1&unlock) | [Firmicutes](https://www.ncbi.nlm.nih.gov/Taxonomy/Browser/wwwtax.cgi?mode=Undef&id=1239&lvl=3&keep=1&srchmode=1&unlock) | [Bacteria](https://www.ncbi.nlm.nih.gov/Taxonomy/Browser/wwwtax.cgi?mode=Undef&id=2&lvl=3&keep=1&srchmode=1&unlock) |
| 91 | 99.54% | *0* | ***Caulobacter sp*** | [AB979861.1](https://www.ncbi.nlm.nih.gov/nucleotide/AB979861.1?report=genbank&log$=nucltop&blast_rank=76&RID=3AHFBG6D01R) | [*Caulobacter*](https://www.ncbi.nlm.nih.gov/Taxonomy/Browser/wwwtax.cgi?mode=Undef&id=75&lvl=3&keep=1&srchmode=1&unlock) | [Caulobacteraceae](https://www.ncbi.nlm.nih.gov/Taxonomy/Browser/wwwtax.cgi?mode=Undef&id=76892&lvl=3&keep=1&srchmode=1&unlock) | [Caulobacterales](https://www.ncbi.nlm.nih.gov/Taxonomy/Browser/wwwtax.cgi?mode=Undef&id=204458&lvl=3&keep=1&srchmode=1&unlock) | [Alphaproteobacteria](https://www.ncbi.nlm.nih.gov/Taxonomy/Browser/wwwtax.cgi?mode=Undef&id=28211&lvl=3&keep=1&srchmode=1&unlock) | [Proteobacteria](https://www.ncbi.nlm.nih.gov/Taxonomy/Browser/wwwtax.cgi?mode=Undef&id=1224&lvl=3&keep=1&srchmode=1&unlock) | [Bacteria](https://www.ncbi.nlm.nih.gov/Taxonomy/Browser/wwwtax.cgi?mode=Undef&id=2&lvl=3&keep=1&srchmode=1&unlock) |
| 92 | 99.66% | *0* | ***Pseudomonas alcaliphila*** | [MT636466.1](https://www.ncbi.nlm.nih.gov/nucleotide/MT636466.1?report=genbank&log$=nucltop&blast_rank=2&RID=3AHSDJ0101R) | [*Pseudomonas*](https://www.ncbi.nlm.nih.gov/Taxonomy/Browser/wwwtax.cgi?mode=Undef&id=286&lvl=3&keep=1&srchmode=1&unlock) | [Pseudomonadaceae](https://www.ncbi.nlm.nih.gov/Taxonomy/Browser/wwwtax.cgi?mode=Undef&id=135621&lvl=3&keep=1&srchmode=1&unlock) | [Pseudomonadales](https://www.ncbi.nlm.nih.gov/Taxonomy/Browser/wwwtax.cgi?mode=Undef&id=72274&lvl=3&keep=1&srchmode=1&unlock) | [Gammaproteobacteria](https://www.ncbi.nlm.nih.gov/Taxonomy/Browser/wwwtax.cgi?mode=Undef&id=1236&lvl=3&keep=1&srchmode=1&unlock) | [Proteobacteria](https://www.ncbi.nlm.nih.gov/Taxonomy/Browser/wwwtax.cgi?mode=Undef&id=1224&lvl=3&keep=1&srchmode=1&unlock) | [Bacteria](https://www.ncbi.nlm.nih.gov/Taxonomy/Browser/wwwtax.cgi?mode=Undef&id=2&lvl=3&keep=1&srchmode=1&unlock) |
| 93 | 99.54% | *0* | ***Neisseria mucosa*** | [CP020452.2](https://www.ncbi.nlm.nih.gov/nucleotide/CP020452.2?report=genbank&log$=nucltop&blast_rank=7&RID=3AHZ3M8N013) | [*Neisseria*](https://www.ncbi.nlm.nih.gov/Taxonomy/Browser/wwwtax.cgi?mode=Undef&id=482&lvl=3&keep=1&srchmode=1&unlock) | [Neisseriaceae](https://www.ncbi.nlm.nih.gov/Taxonomy/Browser/wwwtax.cgi?mode=Undef&id=481&lvl=3&keep=1&srchmode=1&unlock) | [Neisseriales](https://www.ncbi.nlm.nih.gov/Taxonomy/Browser/wwwtax.cgi?mode=Undef&id=206351&lvl=3&keep=1&srchmode=1&unlock) | [Betaproteobacteria](https://www.ncbi.nlm.nih.gov/Taxonomy/Browser/wwwtax.cgi?mode=Undef&id=28216&lvl=3&keep=1&srchmode=1&unlock); | [Proteobacteria](https://www.ncbi.nlm.nih.gov/Taxonomy/Browser/wwwtax.cgi?mode=Undef&id=1224&lvl=3&keep=1&srchmode=1&unlock) | [Bacteria](https://www.ncbi.nlm.nih.gov/Taxonomy/Browser/wwwtax.cgi?mode=Undef&id=2&lvl=3&keep=1&srchmode=1&unlock) |
| 94 | 97.41% | *0* | ***Aeromonas caviae*** | [KU975025.1](https://www.ncbi.nlm.nih.gov/nucleotide/KU975025.1?report=genbank&log$=nucltop&blast_rank=2&RID=3AJ84BYH01R) | [*Aeromonas*](https://www.ncbi.nlm.nih.gov/Taxonomy/Browser/wwwtax.cgi?mode=Undef&id=642&lvl=3&keep=1&srchmode=1&unlock) | [Aeromonadaceae](https://www.ncbi.nlm.nih.gov/Taxonomy/Browser/wwwtax.cgi?mode=Undef&id=84642&lvl=3&keep=1&srchmode=1&unlock) | [Aeromonadales](https://www.ncbi.nlm.nih.gov/Taxonomy/Browser/wwwtax.cgi?mode=Undef&id=135624&lvl=3&keep=1&srchmode=1&unlock) | [Gammaproteobacteria](https://www.ncbi.nlm.nih.gov/Taxonomy/Browser/wwwtax.cgi?mode=Undef&id=1236&lvl=3&keep=1&srchmode=1&unlock) | [Proteobacteria](https://www.ncbi.nlm.nih.gov/Taxonomy/Browser/wwwtax.cgi?mode=Undef&id=1224&lvl=3&keep=1&srchmode=1&unlock) | [Bacteria](https://www.ncbi.nlm.nih.gov/Taxonomy/Browser/wwwtax.cgi?mode=Undef&id=2&lvl=3&keep=1&srchmode=1&unlock) |
| 95 | 94.34% | *0* | ***Streptomyces praecox*** | [JQ924402.1](https://www.ncbi.nlm.nih.gov/nucleotide/JQ924402.1?report=genbank&log$=nucltop&blast_rank=10&RID=3AJEJBUT01R) | [*Streptomyces*](https://www.ncbi.nlm.nih.gov/Taxonomy/Browser/wwwtax.cgi?mode=Undef&id=1883&lvl=3&keep=1&srchmode=1&unlock) | [treptomycetaceae](https://www.ncbi.nlm.nih.gov/Taxonomy/Browser/wwwtax.cgi?mode=Undef&id=2062&lvl=3&keep=1&srchmode=1&unlock) | [Streptomycetales](https://www.ncbi.nlm.nih.gov/Taxonomy/Browser/wwwtax.cgi?mode=Undef&id=85011&lvl=3&keep=1&srchmode=1&unlock) | [Actinomycetia](https://www.ncbi.nlm.nih.gov/Taxonomy/Browser/wwwtax.cgi?mode=Undef&id=1760&lvl=3&keep=1&srchmode=1&unlock) | [Actinobacteria](https://www.ncbi.nlm.nih.gov/Taxonomy/Browser/wwwtax.cgi?mode=Undef&id=201174&lvl=3&keep=1&srchmode=1&unlock) | [Bacteria](https://www.ncbi.nlm.nih.gov/Taxonomy/Browser/wwwtax.cgi?mode=Undef&id=2&lvl=3&keep=1&srchmode=1&unlock) |
| 96 | 99.44% | *0* | ***Methylobacter tundripaludum*** | [NR_042107.1](https://www.ncbi.nlm.nih.gov/nucleotide/NR_042107.1?report=genbank&log$=nucltop&blast_rank=2&RID=3AJNTX6301R) | [*Methylobacter*](https://www.ncbi.nlm.nih.gov/Taxonomy/Browser/wwwtax.cgi?mode=Undef&id=429&lvl=3&keep=1&srchmode=1&unlock) | [Methylococcaceae](https://www.ncbi.nlm.nih.gov/Taxonomy/Browser/wwwtax.cgi?mode=Undef&id=403&lvl=3&keep=1&srchmode=1&unlock) | [Methylococcales](https://www.ncbi.nlm.nih.gov/Taxonomy/Browser/wwwtax.cgi?mode=Undef&id=135618&lvl=3&keep=1&srchmode=1&unlock) | [Gammaproteobacteria](https://www.ncbi.nlm.nih.gov/Taxonomy/Browser/wwwtax.cgi?mode=Undef&id=1236&lvl=3&keep=1&srchmode=1&unlock) | [Proteobacteria](https://www.ncbi.nlm.nih.gov/Taxonomy/Browser/wwwtax.cgi?mode=Undef&id=1224&lvl=3&keep=1&srchmode=1&unlock) | [Bacteria](https://www.ncbi.nlm.nih.gov/Taxonomy/Browser/wwwtax.cgi?mode=Undef&id=2&lvl=3&keep=1&srchmode=1&unlock) |
| 97 | 99.29% | *0* | ***Methylobacter tundripaludum*** | [NR_042107.1](https://www.ncbi.nlm.nih.gov/nucleotide/NR_042107.1?report=genbank&log$=nucltop&blast_rank=3&RID=3AJXRJJN013) | [*Methylobacter*](https://www.ncbi.nlm.nih.gov/Taxonomy/Browser/wwwtax.cgi?mode=Undef&id=429&lvl=3&keep=1&srchmode=1&unlock) | [Methylococcaceae](https://www.ncbi.nlm.nih.gov/Taxonomy/Browser/wwwtax.cgi?mode=Undef&id=403&lvl=3&keep=1&srchmode=1&unlock) | [Methylococcales](https://www.ncbi.nlm.nih.gov/Taxonomy/Browser/wwwtax.cgi?mode=Undef&id=135618&lvl=3&keep=1&srchmode=1&unlock) | [Gammaproteobacteria](https://www.ncbi.nlm.nih.gov/Taxonomy/Browser/wwwtax.cgi?mode=Undef&id=1236&lvl=3&keep=1&srchmode=1&unlock) | [Proteobacteria](https://www.ncbi.nlm.nih.gov/Taxonomy/Browser/wwwtax.cgi?mode=Undef&id=1224&lvl=3&keep=1&srchmode=1&unlock) | [Bacteria](https://www.ncbi.nlm.nih.gov/Taxonomy/Browser/wwwtax.cgi?mode=Undef&id=2&lvl=3&keep=1&srchmode=1&unlock) |
| 98 | 98.65% | *0* | ***Methylobacter tundripaludum*** | [J[NR_042107.1](https://www.ncbi.nlm.nih.gov/nucleotide/NR_042107.1?report=genbank&log$=nucltop&blast_rank=7&RID=3AK3Z9MA013)](https://www.ncbi.nlm.nih.gov/nucleotide/JN656724.1?report=genbank&log$=nucltop&blast_rank=6&RID=3AK3Z9MA013) | [*Methylobacter*](https://www.ncbi.nlm.nih.gov/Taxonomy/Browser/wwwtax.cgi?mode=Undef&id=429&lvl=3&keep=1&srchmode=1&unlock) | [Methylococcaceae](https://www.ncbi.nlm.nih.gov/Taxonomy/Browser/wwwtax.cgi?mode=Undef&id=403&lvl=3&keep=1&srchmode=1&unlock) | [Methylococcales](https://www.ncbi.nlm.nih.gov/Taxonomy/Browser/wwwtax.cgi?mode=Undef&id=135618&lvl=3&keep=1&srchmode=1&unlock) | [Gammaproteobacteria](https://www.ncbi.nlm.nih.gov/Taxonomy/Browser/wwwtax.cgi?mode=Undef&id=1236&lvl=3&keep=1&srchmode=1&unlock) | [Proteobacteria](https://www.ncbi.nlm.nih.gov/Taxonomy/Browser/wwwtax.cgi?mode=Undef&id=1224&lvl=3&keep=1&srchmode=1&unlock) | [Bacteria](https://www.ncbi.nlm.nih.gov/Taxonomy/Browser/wwwtax.cgi?mode=Undef&id=2&lvl=3&keep=1&srchmode=1&unlock) |
| 99 | 89% | *0* | ***Methylobacter tundripaludum*** | [NR_042107.1](https://www.ncbi.nlm.nih.gov/nucleotide/NR_042107.1?report=genbank&log$=nucltop&blast_rank=9&RID=3AK94PGR016) | [*Methylobacter*](https://www.ncbi.nlm.nih.gov/Taxonomy/Browser/wwwtax.cgi?mode=Undef&id=429&lvl=3&keep=1&srchmode=1&unlock) | [Methylococcaceae](https://www.ncbi.nlm.nih.gov/Taxonomy/Browser/wwwtax.cgi?mode=Undef&id=403&lvl=3&keep=1&srchmode=1&unlock) | [Methylococcales](https://www.ncbi.nlm.nih.gov/Taxonomy/Browser/wwwtax.cgi?mode=Undef&id=135618&lvl=3&keep=1&srchmode=1&unlock) | [Gammaproteobacteria](https://www.ncbi.nlm.nih.gov/Taxonomy/Browser/wwwtax.cgi?mode=Undef&id=1236&lvl=3&keep=1&srchmode=1&unlock) | [Proteobacteria](https://www.ncbi.nlm.nih.gov/Taxonomy/Browser/wwwtax.cgi?mode=Undef&id=1224&lvl=3&keep=1&srchmode=1&unlock) | [Bacteria](https://www.ncbi.nlm.nih.gov/Taxonomy/Browser/wwwtax.cgi?mode=Undef&id=2&lvl=3&keep=1&srchmode=1&unlock) |
| 100 | 99.66% | *0* | ***Domibacillus enclensis*** | [NR_134021.1](https://www.ncbi.nlm.nih.gov/nucleotide/NR_134021.1?report=genbank&log$=nucltop&blast_rank=6&RID=3AKEJ65201R) | [*Domibacillus*](https://www.ncbi.nlm.nih.gov/Taxonomy/Browser/wwwtax.cgi?mode=Undef&id=1433999&lvl=3&keep=1&srchmode=1&unlock) | [Bacillaceae](https://www.ncbi.nlm.nih.gov/Taxonomy/Browser/wwwtax.cgi?mode=Undef&id=186817&lvl=3&keep=1&srchmode=1&unlock) | [Bacillales](https://www.ncbi.nlm.nih.gov/Taxonomy/Browser/wwwtax.cgi?mode=Undef&id=1385&lvl=3&keep=1&srchmode=1&unlock) | [Bacilli](https://www.ncbi.nlm.nih.gov/Taxonomy/Browser/wwwtax.cgi?mode=Undef&id=91061&lvl=3&keep=1&srchmode=1&unlock) | [Firmicutes](https://www.ncbi.nlm.nih.gov/Taxonomy/Browser/wwwtax.cgi?mode=Undef&id=1239&lvl=3&keep=1&srchmode=1&unlock) | [Bacteria](https://www.ncbi.nlm.nih.gov/Taxonomy/Browser/wwwtax.cgi?mode=Undef&id=2&lvl=3&keep=1&srchmode=1&unlock) |
| 101 | 99.19% | *0* | ***Domibacillus enclensis*** | [NR_134021.1](https://www.ncbi.nlm.nih.gov/nucleotide/NR_134021.1?report=genbank&log$=nucltop&blast_rank=6&RID=3AKR5S6N01R) | [*Domibacillus*](https://www.ncbi.nlm.nih.gov/Taxonomy/Browser/wwwtax.cgi?mode=Undef&id=1433999&lvl=3&keep=1&srchmode=1&unlock) | [Bacillaceae](https://www.ncbi.nlm.nih.gov/Taxonomy/Browser/wwwtax.cgi?mode=Undef&id=186817&lvl=3&keep=1&srchmode=1&unlock) | [Bacillales](https://www.ncbi.nlm.nih.gov/Taxonomy/Browser/wwwtax.cgi?mode=Undef&id=1385&lvl=3&keep=1&srchmode=1&unlock) | [Bacilli](https://www.ncbi.nlm.nih.gov/Taxonomy/Browser/wwwtax.cgi?mode=Undef&id=91061&lvl=3&keep=1&srchmode=1&unlock) | [Firmicutes](https://www.ncbi.nlm.nih.gov/Taxonomy/Browser/wwwtax.cgi?mode=Undef&id=1239&lvl=3&keep=1&srchmode=1&unlock) | [Bacteria](https://www.ncbi.nlm.nih.gov/Taxonomy/Browser/wwwtax.cgi?mode=Undef&id=2&lvl=3&keep=1&srchmode=1&unlock) |
| 102 | 99.44% | *0* | ***Domibacillus enclensis*** | [NR_134021.1](https://www.ncbi.nlm.nih.gov/nucleotide/NR_134021.1?report=genbank&log$=nucltop&blast_rank=6&RID=3AKVT3DK01R) | [*Domibacillus*](https://www.ncbi.nlm.nih.gov/Taxonomy/Browser/wwwtax.cgi?mode=Undef&id=1433999&lvl=3&keep=1&srchmode=1&unlock) | [Bacillaceae](https://www.ncbi.nlm.nih.gov/Taxonomy/Browser/wwwtax.cgi?mode=Undef&id=186817&lvl=3&keep=1&srchmode=1&unlock) | [Bacillales](https://www.ncbi.nlm.nih.gov/Taxonomy/Browser/wwwtax.cgi?mode=Undef&id=1385&lvl=3&keep=1&srchmode=1&unlock) | [Bacilli](https://www.ncbi.nlm.nih.gov/Taxonomy/Browser/wwwtax.cgi?mode=Undef&id=91061&lvl=3&keep=1&srchmode=1&unlock) | [Firmicutes](https://www.ncbi.nlm.nih.gov/Taxonomy/Browser/wwwtax.cgi?mode=Undef&id=1239&lvl=3&keep=1&srchmode=1&unlock) | [Bacteria](https://www.ncbi.nlm.nih.gov/Taxonomy/Browser/wwwtax.cgi?mode=Undef&id=2&lvl=3&keep=1&srchmode=1&unlock) |
| 104 | 100% | *0* | ***Terasakiella brassicae*** | [NR_148851.1](https://www.ncbi.nlm.nih.gov/nucleotide/NR_148851.1?report=genbank&log$=nucltop&blast_rank=1&RID=3AM6V8C801R) | [*Terasakiella*](https://www.ncbi.nlm.nih.gov/Taxonomy/Browser/wwwtax.cgi?mode=Undef&id=196080&lvl=3&keep=1&srchmode=1&unlock) | [Terasakiellaceae](https://www.ncbi.nlm.nih.gov/Taxonomy/Browser/wwwtax.cgi?mode=Undef&id=2813951&lvl=3&keep=1&srchmode=1&unlock) | [Rhodospirillales](https://www.ncbi.nlm.nih.gov/Taxonomy/Browser/wwwtax.cgi?mode=Undef&id=204441&lvl=3&keep=1&srchmode=1&unlock) | [Alphaproteobacteria](https://www.ncbi.nlm.nih.gov/Taxonomy/Browser/wwwtax.cgi?mode=Undef&id=28211&lvl=3&keep=1&srchmode=1&unlock) | [Proteobacteria](https://www.ncbi.nlm.nih.gov/Taxonomy/Browser/wwwtax.cgi?mode=Undef&id=1224&lvl=3&keep=1&srchmode=1&unlock) | [Bacteria](https://www.ncbi.nlm.nih.gov/Taxonomy/Browser/wwwtax.cgi?mode=Undef&id=2&lvl=3&keep=1&srchmode=1&unlock) |
| 105 | 99.78% | *0* | ***Nesiotobacter exalbescens*** | [KU996359.1](https://www.ncbi.nlm.nih.gov/nucleotide/KU996359.1?report=genbank&log$=nucltop&blast_rank=3&RID=3AMD1UYW01R) | [*Nesiotobacter*](https://www.ncbi.nlm.nih.gov/Taxonomy/Browser/wwwtax.cgi?mode=Undef&id=500577&lvl=3&keep=1&srchmode=1&unlock) | [Rhodobacteraceae](https://www.ncbi.nlm.nih.gov/Taxonomy/Browser/wwwtax.cgi?mode=Undef&id=31989&lvl=3&keep=1&srchmode=1&unlock) | [Rhodobacterales](https://www.ncbi.nlm.nih.gov/Taxonomy/Browser/wwwtax.cgi?mode=Undef&id=204455&lvl=3&keep=1&srchmode=1&unlock) | [Alphaproteobacteria](https://www.ncbi.nlm.nih.gov/Taxonomy/Browser/wwwtax.cgi?mode=Undef&id=28211&lvl=3&keep=1&srchmode=1&unlock) | [Proteobacteria](https://www.ncbi.nlm.nih.gov/Taxonomy/Browser/wwwtax.cgi?mode=Undef&id=1224&lvl=3&keep=1&srchmode=1&unlock) | [Bacteria](https://www.ncbi.nlm.nih.gov/Taxonomy/Browser/wwwtax.cgi?mode=Undef&id=2&lvl=3&keep=1&srchmode=1&unlock) |
| 106 | 99.39% | *0* | ***Sedimenticola selenatireducens*** | [KM192219.1](https://www.ncbi.nlm.nih.gov/nucleotide/KM192219.1?report=genbank&log$=nucltop&blast_rank=5&RID=3AN5ZAW6013) | [*Sedimenticola*](https://www.ncbi.nlm.nih.gov/Taxonomy/Browser/wwwtax.cgi?mode=Undef&id=349742&lvl=3&keep=1&srchmode=1&unlock) |  | [unclassified Gammaproteobacteria](https://en.wikipedia.org/w/index.php?title=Unclassified_Gammaproteobacteria&action=edit&redlink=1) | [Gammaproteobacteria](https://www.ncbi.nlm.nih.gov/Taxonomy/Browser/wwwtax.cgi?mode=Undef&id=1236&lvl=3&keep=1&srchmode=1&unlock) | [Proteobacteria](https://www.ncbi.nlm.nih.gov/Taxonomy/Browser/wwwtax.cgi?mode=Undef&id=1224&lvl=3&keep=1&srchmode=1&unlock) | [Bacteria](https://www.ncbi.nlm.nih.gov/Taxonomy/Browser/wwwtax.cgi?mode=Undef&id=2&lvl=3&keep=1&srchmode=1&unlock) |
| 107 | 98.17 | *0* | ***Sedimenticola selenatireducens*** | [KM192219.1](https://www.ncbi.nlm.nih.gov/nucleotide/KM192219.1?report=genbank&log$=nucltop&blast_rank=22&RID=3ANKR8X8016) | [*Sedimenticola*](https://www.ncbi.nlm.nih.gov/Taxonomy/Browser/wwwtax.cgi?mode=Undef&id=349742&lvl=3&keep=1&srchmode=1&unlock) |  | [unclassified Gammaproteobacteria](https://en.wikipedia.org/w/index.php?title=Unclassified_Gammaproteobacteria&action=edit&redlink=1) | [Gammaproteobacteria](https://www.ncbi.nlm.nih.gov/Taxonomy/Browser/wwwtax.cgi?mode=Undef&id=1236&lvl=3&keep=1&srchmode=1&unlock) | [Proteobacteria](https://www.ncbi.nlm.nih.gov/Taxonomy/Browser/wwwtax.cgi?mode=Undef&id=1224&lvl=3&keep=1&srchmode=1&unlock) | [Bacteria](https://www.ncbi.nlm.nih.gov/Taxonomy/Browser/wwwtax.cgi?mode=Undef&id=2&lvl=3&keep=1&srchmode=1&unlock) |
| 108 | 98.67% | *0* | ***Leisingera aquaemixtae*** | [CP041159.1](https://www.ncbi.nlm.nih.gov/nucleotide/CP041159.1?report=genbank&log$=nucltop&blast_rank=2&RID=3ANTZBWT01R) | [*Leisingera*](https://www.ncbi.nlm.nih.gov/Taxonomy/Browser/wwwtax.cgi?mode=Undef&id=191028&lvl=3&keep=1&srchmode=1&unlock) | [Rhodobacteraceae](https://www.ncbi.nlm.nih.gov/Taxonomy/Browser/wwwtax.cgi?mode=Undef&id=31989&lvl=3&keep=1&srchmode=1&unlock) | [Rhodobacterales](https://www.ncbi.nlm.nih.gov/Taxonomy/Browser/wwwtax.cgi?mode=Undef&id=204455&lvl=3&keep=1&srchmode=1&unlock) | [Alphaproteobacteria](https://www.ncbi.nlm.nih.gov/Taxonomy/Browser/wwwtax.cgi?mode=Undef&id=28211&lvl=3&keep=1&srchmode=1&unlock) | [Proteobacteria](https://www.ncbi.nlm.nih.gov/Taxonomy/Browser/wwwtax.cgi?mode=Undef&id=1224&lvl=3&keep=1&srchmode=1&unlock) | [Bacteria](https://www.ncbi.nlm.nih.gov/Taxonomy/Browser/wwwtax.cgi?mode=Undef&id=2&lvl=3&keep=1&srchmode=1&unlock) |
| 109 | 99.19% | *0* | ***Halodesulfovibrio marinisediminis*** | [NR_041631.1](https://www.ncbi.nlm.nih.gov/nucleotide/NR_041631.1?report=genbank&log$=nucltop&blast_rank=7&RID=3AP7UTN901R) | [*Halodesulfovibrio*](https://www.ncbi.nlm.nih.gov/Taxonomy/Browser/wwwtax.cgi?mode=Undef&id=1912771&lvl=3&keep=1&srchmode=1&unlock) | [Desulfovibrionaceae](https://www.ncbi.nlm.nih.gov/Taxonomy/Browser/wwwtax.cgi?mode=Undef&id=194924&lvl=3&keep=1&srchmode=1&unlock) | [Desulfovibrionales](https://www.ncbi.nlm.nih.gov/Taxonomy/Browser/wwwtax.cgi?mode=Undef&id=213115&lvl=3&keep=1&srchmode=1&unlock) | [Deltaproteobacteria](https://www.ncbi.nlm.nih.gov/Taxonomy/Browser/wwwtax.cgi?mode=Undef&id=28221&lvl=3&keep=1&srchmode=1&unlock) | [Proteobacteria](https://www.ncbi.nlm.nih.gov/Taxonomy/Browser/wwwtax.cgi?mode=Undef&id=1224&lvl=3&keep=1&srchmode=1&unlock) | [Bacteria](https://www.ncbi.nlm.nih.gov/Taxonomy/Browser/wwwtax.cgi?mode=Undef&id=2&lvl=3&keep=1&srchmode=1&unlock) |
| 110 | 96.64% | *0* | ***Sulfurimonas crateris*** | [MK859925.1](https://www.ncbi.nlm.nih.gov/nucleotide/MK859925.1?report=genbank&log$=nucltop&blast_rank=4&RID=3APRVV3D01R) | [*Sulfurimonas*](https://www.ncbi.nlm.nih.gov/Taxonomy/Browser/wwwtax.cgi?mode=Undef&id=202746&lvl=3&keep=1&srchmode=1&unlock) | [Thiovulaceae](https://www.ncbi.nlm.nih.gov/Taxonomy/Browser/wwwtax.cgi?mode=Undef&id=2771471&lvl=3&keep=1&srchmode=1&unlock) | [Campylobacterales](https://www.ncbi.nlm.nih.gov/Taxonomy/Browser/wwwtax.cgi?mode=Undef&id=213849&lvl=3&keep=1&srchmode=1&unlock) | [Epsilonproteobacteri](https://www.ncbi.nlm.nih.gov/Taxonomy/Browser/wwwtax.cgi?mode=Undef&id=29547&lvl=3&keep=1&srchmode=1&unlock) | [Proteobacteria](https://www.ncbi.nlm.nih.gov/Taxonomy/Browser/wwwtax.cgi?mode=Undef&id=1224&lvl=3&keep=1&srchmode=1&unlock) | [Bacteria](https://www.ncbi.nlm.nih.gov/Taxonomy/Browser/wwwtax.cgi?mode=Undef&id=2&lvl=3&keep=1&srchmode=1&unlock) |
| 111 | 99.41% | *0* | ***Desulfovibrio salexigens*** | [NR_102801.1](https://www.ncbi.nlm.nih.gov/nucleotide/NR_102801.1?report=genbank&log$=nucltop&blast_rank=3&RID=3APXZBYT01R) | [*Maridesulfovibrio*](https://www.ncbi.nlm.nih.gov/Taxonomy/Browser/wwwtax.cgi?mode=Undef&id=2794998&lvl=3&keep=1&srchmode=1&unlock) | [Desulfovibrionaceae](https://www.ncbi.nlm.nih.gov/Taxonomy/Browser/wwwtax.cgi?mode=Undef&id=194924&lvl=3&keep=1&srchmode=1&unlock) | [Desulfovibrionales](https://www.ncbi.nlm.nih.gov/Taxonomy/Browser/wwwtax.cgi?mode=Undef&id=213115&lvl=3&keep=1&srchmode=1&unlock) | [Deltaproteobacteria](https://www.ncbi.nlm.nih.gov/Taxonomy/Browser/wwwtax.cgi?mode=Undef&id=28221&lvl=3&keep=1&srchmode=1&unlock) | [Proteobacteria](https://www.ncbi.nlm.nih.gov/Taxonomy/Browser/wwwtax.cgi?mode=Undef&id=1224&lvl=3&keep=1&srchmode=1&unlock) | [Bacteria](https://www.ncbi.nlm.nih.gov/Taxonomy/Browser/wwwtax.cgi?mode=Undef&id=2&lvl=3&keep=1&srchmode=1&unlock) |
| 112 | 98.75% | *0* | ***Geotoga subterranea*** | [NR_029145.2](https://www.ncbi.nlm.nih.gov/nucleotide/NR_029145.2?report=genbank&log$=nucltop&blast_rank=6&RID=3AR3SV9M01R) | [*Geotoga*](https://www.ncbi.nlm.nih.gov/Taxonomy/Browser/wwwtax.cgi?mode=Undef&id=28233&lvl=3&keep=1&srchmode=1&unlock) | [Petrotogaceae](https://www.ncbi.nlm.nih.gov/Taxonomy/Browser/wwwtax.cgi?mode=Undef&id=1643949&lvl=3&keep=1&srchmode=1&unlock) | [Petrotogales](https://www.ncbi.nlm.nih.gov/Taxonomy/Browser/wwwtax.cgi?mode=Undef&id=1643947&lvl=3&keep=1&srchmode=1&unlock) | [Thermotogae](https://www.ncbi.nlm.nih.gov/Taxonomy/Browser/wwwtax.cgi?mode=Undef&id=188708&lvl=3&keep=1&srchmode=1&unlock) | [Thermotogae](https://www.ncbi.nlm.nih.gov/Taxonomy/Browser/wwwtax.cgi?mode=Undef&id=200918&lvl=3&keep=1&srchmode=1&unlock) | [Bacteria](https://www.ncbi.nlm.nih.gov/Taxonomy/Browser/wwwtax.cgi?mode=Undef&id=2&lvl=3&keep=1&srchmode=1&unlock) |
| 113 | 99.6% | *0* | ***Sphaerochaeta sp.*** | [MH456879.2](https://www.ncbi.nlm.nih.gov/nucleotide/MH456879.2?report=genbank&log$=nucltop&blast_rank=3&RID=3ARETUVU01R) | [*Sphaerochaeta*](https://www.ncbi.nlm.nih.gov/Taxonomy/Browser/wwwtax.cgi?mode=Undef&id=399320&lvl=3&keep=1&srchmode=1&unlock) | [Spirochaetaceae](https://www.ncbi.nlm.nih.gov/Taxonomy/Browser/wwwtax.cgi?mode=Undef&id=137&lvl=3&keep=1&srchmode=1&unlock) | [Spirochaetales](https://www.ncbi.nlm.nih.gov/Taxonomy/Browser/wwwtax.cgi?mode=Undef&id=136&lvl=3&keep=1&srchmode=1&unlock) | [Spirochaetia](https://www.ncbi.nlm.nih.gov/Taxonomy/Browser/wwwtax.cgi?mode=Undef&id=203692&lvl=3&keep=1&srchmode=1&unlock) | [Spirochaetes](https://www.ncbi.nlm.nih.gov/Taxonomy/Browser/wwwtax.cgi?mode=Undef&id=203691&lvl=3&keep=1&srchmode=1&unlock) | [Bacteria](https://www.ncbi.nlm.nih.gov/Taxonomy/Browser/wwwtax.cgi?mode=Undef&id=2&lvl=3&keep=1&srchmode=1&unlock) |
| 114 | 99.6 % | *0* | ***Sphaerochaeta sp.*** | [MH456879.2](https://www.ncbi.nlm.nih.gov/nucleotide/MH456879.2?report=genbank&log$=nucltop&blast_rank=2&RID=3ARM24ZZ01R) | [*Sphaerochaeta*](https://www.ncbi.nlm.nih.gov/Taxonomy/Browser/wwwtax.cgi?mode=Undef&id=399320&lvl=3&keep=1&srchmode=1&unlock) | [Spirochaetaceae](https://www.ncbi.nlm.nih.gov/Taxonomy/Browser/wwwtax.cgi?mode=Undef&id=137&lvl=3&keep=1&srchmode=1&unlock) | [Spirochaetales](https://www.ncbi.nlm.nih.gov/Taxonomy/Browser/wwwtax.cgi?mode=Undef&id=136&lvl=3&keep=1&srchmode=1&unlock) | [Spirochaetia](https://www.ncbi.nlm.nih.gov/Taxonomy/Browser/wwwtax.cgi?mode=Undef&id=203692&lvl=3&keep=1&srchmode=1&unlock) | [Spirochaetes](https://www.ncbi.nlm.nih.gov/Taxonomy/Browser/wwwtax.cgi?mode=Undef&id=203691&lvl=3&keep=1&srchmode=1&unlock) | [Bacteria](https://www.ncbi.nlm.nih.gov/Taxonomy/Browser/wwwtax.cgi?mode=Undef&id=2&lvl=3&keep=1&srchmode=1&unlock) |
| 115* | 99.6% | *0* |  | [GU136592.1](https://www.ncbi.nlm.nih.gov/nucleotide/GU136592.1?report=genbank&log$=nucltop&blast_rank=4&RID=3ARX0WU2016) |  |  |  | [Clostridia](https://www.ncbi.nlm.nih.gov/Taxonomy/Browser/wwwtax.cgi?mode=Undef&id=186801&lvl=3&keep=1&srchmode=1&unlock) | [Firmicutes](https://www.ncbi.nlm.nih.gov/Taxonomy/Browser/wwwtax.cgi?mode=Undef&id=1239&lvl=3&keep=1&srchmode=1&unlock) | [Bacteria](https://www.ncbi.nlm.nih.gov/Taxonomy/Browser/wwwtax.cgi?mode=Undef&id=2&lvl=3&keep=1&srchmode=1&unlock) |
| 116 | 99.80% | *0* | *Marinobacter sp* | [LC373531.1](https://www.ncbi.nlm.nih.gov/nucleotide/LC373531.1?report=genbank&log$=nucltop&blast_rank=4&RID=3AS2WMET016) | [*Marinobacter*](https://www.ncbi.nlm.nih.gov/Taxonomy/Browser/wwwtax.cgi?mode=Undef&id=2742&lvl=3&keep=1&srchmode=1&unlock) | [Alteromonadaceae](https://www.ncbi.nlm.nih.gov/Taxonomy/Browser/wwwtax.cgi?mode=Undef&id=72275&lvl=3&keep=1&srchmode=1&unlock) | [Alteromonadales](https://www.ncbi.nlm.nih.gov/Taxonomy/Browser/wwwtax.cgi?mode=Undef&id=135622&lvl=3&keep=1&srchmode=1&unlock) | [Gammaproteobacteria](https://www.ncbi.nlm.nih.gov/Taxonomy/Browser/wwwtax.cgi?mode=Undef&id=1236&lvl=3&keep=1&srchmode=1&unlock) | [Proteobacteria](https://www.ncbi.nlm.nih.gov/Taxonomy/Browser/wwwtax.cgi?mode=Undef&id=1224&lvl=3&keep=1&srchmode=1&unlock) | [Bacteria](https://www.ncbi.nlm.nih.gov/Taxonomy/Browser/wwwtax.cgi?mode=Undef&id=2&lvl=3&keep=1&srchmode=1&unlock) |
| 117 | 100% | *0* | ***Desulfotignum balticum*** | [NR_041852.1](https://www.ncbi.nlm.nih.gov/nucleotide/NR_041852.1?report=genbank&log$=nucltop&blast_rank=4&RID=3ASF1KFC01R) | [*Desulfotignum*](https://www.ncbi.nlm.nih.gov/Taxonomy/Browser/wwwtax.cgi?mode=Undef&id=115780&lvl=3&keep=1&srchmode=1&unlock) | [Desulfobacteraceae](https://www.ncbi.nlm.nih.gov/Taxonomy/Browser/wwwtax.cgi?mode=Undef&id=213119&lvl=3&keep=1&srchmode=1&unlock) | [Desulfobacterales](https://www.ncbi.nlm.nih.gov/Taxonomy/Browser/wwwtax.cgi?mode=Undef&id=213118&lvl=3&keep=1&srchmode=1&unlock) | [Deltaproteobacteria](https://www.ncbi.nlm.nih.gov/Taxonomy/Browser/wwwtax.cgi?mode=Undef&id=28221&lvl=3&keep=1&srchmode=1&unlock) | [roteobacteria](https://www.ncbi.nlm.nih.gov/Taxonomy/Browser/wwwtax.cgi?mode=Undef&id=1224&lvl=3&keep=1&srchmode=1&unlock) | [Bacteria](https://www.ncbi.nlm.nih.gov/Taxonomy/Browser/wwwtax.cgi?mode=Undef&id=2&lvl=3&keep=1&srchmode=1&unlock) |
| 118 | 97.06% | *0* | ***Alkaliphilus peptidifermentans*** | [NR_116002.1](https://www.ncbi.nlm.nih.gov/nucleotide/NR_116002.1?report=genbank&log$=nucltop&blast_rank=10&RID=3ASPA471013) | [*Alkaliphilus*](https://www.ncbi.nlm.nih.gov/Taxonomy/Browser/wwwtax.cgi?mode=Undef&id=114627&lvl=3&keep=1&srchmode=1&unlock) | [Clostridiaceae](https://www.ncbi.nlm.nih.gov/Taxonomy/Browser/wwwtax.cgi?mode=Undef&id=31979&lvl=3&keep=1&srchmode=1&unlock) | [Clostridiales](https://www.ncbi.nlm.nih.gov/Taxonomy/Browser/wwwtax.cgi?mode=Undef&id=186802&lvl=3&keep=1&srchmode=1&unlock) | [Clostridia](https://www.ncbi.nlm.nih.gov/Taxonomy/Browser/wwwtax.cgi?mode=Undef&id=186801&lvl=3&keep=1&srchmode=1&unlock) | [Firmicutes](https://www.ncbi.nlm.nih.gov/Taxonomy/Browser/wwwtax.cgi?mode=Undef&id=1239&lvl=3&keep=1&srchmode=1&unlock) | [Bacteria](https://www.ncbi.nlm.nih.gov/Taxonomy/Browser/wwwtax.cgi?mode=Undef&id=2&lvl=3&keep=1&srchmode=1&unlock) |
| 119 | 89.97% | *0* | ***Tepidibacillus decaturensis*** | [NR_152058.1](https://www.ncbi.nlm.nih.gov/nucleotide/NR_152058.1?report=genbank&log$=nucltop&blast_rank=83&RID=3AT691US01R) | [*Tepidibacillus*](https://www.ncbi.nlm.nih.gov/Taxonomy/Browser/wwwtax.cgi?mode=Undef&id=1494427&lvl=3&keep=1&srchmode=1&unlock) | [Bacillaceae](https://www.ncbi.nlm.nih.gov/Taxonomy/Browser/wwwtax.cgi?mode=Undef&id=186817&lvl=3&keep=1&srchmode=1&unlock) | [Bacillales](https://www.ncbi.nlm.nih.gov/Taxonomy/Browser/wwwtax.cgi?mode=Undef&id=1385&lvl=3&keep=1&srchmode=1&unlock) | [Bacilli](https://www.ncbi.nlm.nih.gov/Taxonomy/Browser/wwwtax.cgi?mode=Undef&id=91061&lvl=3&keep=1&srchmode=1&unlock) | [Firmicutes](https://www.ncbi.nlm.nih.gov/Taxonomy/Browser/wwwtax.cgi?mode=Undef&id=1239&lvl=3&keep=1&srchmode=1&unlock) | [Bacteria](https://www.ncbi.nlm.nih.gov/Taxonomy/Browser/wwwtax.cgi?mode=Undef&id=2&lvl=3&keep=1&srchmode=1&unlock) |
| 120 | 91.22% | *0* | ***Shewanella algae*** | [DQ386137.1](https://www.ncbi.nlm.nih.gov/nucleotide/DQ386137.1?report=genbank&log$=nucltop&blast_rank=4&RID=3ATDWTJ501R) | [*Shewanella*](https://www.ncbi.nlm.nih.gov/Taxonomy/Browser/wwwtax.cgi?mode=Undef&id=22&lvl=3&keep=1&srchmode=1&unlock) | [Shewanellaceae](https://www.ncbi.nlm.nih.gov/Taxonomy/Browser/wwwtax.cgi?mode=Undef&id=267890&lvl=3&keep=1&srchmode=1&unlock) | [Alteromonadales](https://www.ncbi.nlm.nih.gov/Taxonomy/Browser/wwwtax.cgi?mode=Undef&id=135622&lvl=3&keep=1&srchmode=1&unlock) | [Gammaproteobacteria](https://www.ncbi.nlm.nih.gov/Taxonomy/Browser/wwwtax.cgi?mode=Undef&id=1236&lvl=3&keep=1&srchmode=1&unlock) | [Proteobacteria](https://www.ncbi.nlm.nih.gov/Taxonomy/Browser/wwwtax.cgi?mode=Undef&id=1224&lvl=3&keep=1&srchmode=1&unlock) | [Bacteria](https://www.ncbi.nlm.nih.gov/Taxonomy/Browser/wwwtax.cgi?mode=Undef&id=2&lvl=3&keep=1&srchmode=1&unlock) |
| 121 | 97.29% | *0* | ***Shewanella algae*** | [CP033575.1](https://www.ncbi.nlm.nih.gov/nucleotide/CP033575.1?report=genbank&log$=nucltop&blast_rank=2&RID=3ATNZYRP01R) | [*Shewanella*](https://www.ncbi.nlm.nih.gov/Taxonomy/Browser/wwwtax.cgi?mode=Undef&id=22&lvl=3&keep=1&srchmode=1&unlock) | [Shewanellaceae](https://www.ncbi.nlm.nih.gov/Taxonomy/Browser/wwwtax.cgi?mode=Undef&id=267890&lvl=3&keep=1&srchmode=1&unlock) | [Alteromonadales](https://www.ncbi.nlm.nih.gov/Taxonomy/Browser/wwwtax.cgi?mode=Undef&id=135622&lvl=3&keep=1&srchmode=1&unlock) | [Gammaproteobacteria](https://www.ncbi.nlm.nih.gov/Taxonomy/Browser/wwwtax.cgi?mode=Undef&id=1236&lvl=3&keep=1&srchmode=1&unlock) | [Proteobacteria](https://www.ncbi.nlm.nih.gov/Taxonomy/Browser/wwwtax.cgi?mode=Undef&id=1224&lvl=3&keep=1&srchmode=1&unlock) | [Bacteria](https://www.ncbi.nlm.nih.gov/Taxonomy/Browser/wwwtax.cgi?mode=Undef&id=2&lvl=3&keep=1&srchmode=1&unlock) |
| 122 | 97.75% | *0* | ***Terrimonas sp.*** | [MK402934.2](https://www.ncbi.nlm.nih.gov/nucleotide/MK402934.2?report=genbank&log$=nucltop&blast_rank=7&RID=3ATV9WTW01R) | [*Terrimonas*](https://www.ncbi.nlm.nih.gov/Taxonomy/Browser/wwwtax.cgi?mode=Undef&id=296051&lvl=3&keep=1&srchmode=1&unlock) | [Chitinophagaceae](https://www.ncbi.nlm.nih.gov/Taxonomy/Browser/wwwtax.cgi?mode=Undef&id=563835&lvl=3&keep=1&srchmode=1&unlock) | [Chitinophagales](https://www.ncbi.nlm.nih.gov/Taxonomy/Browser/wwwtax.cgi?mode=Undef&id=1853229&lvl=3&keep=1&srchmode=1&unlock) | [Chitinophagi](https://www.ncbi.nlm.nih.gov/Taxonomy/Browser/wwwtax.cgi?mode=Undef&id=1853228&lvl=3&keep=1&srchmode=1&unlock) | [Bacteroidetes](https://www.ncbi.nlm.nih.gov/Taxonomy/Browser/wwwtax.cgi?mode=Undef&id=68336&lvl=3&keep=1&srchmode=1&unlock) | [Bacteria](https://www.ncbi.nlm.nih.gov/Taxonomy/Browser/wwwtax.cgi?mode=Undef&id=2&lvl=3&keep=1&srchmode=1&unlock) |
| 123 | 95.83% | *0* | ***Chitinophaga pinensis*** | [KF228163.1](https://www.ncbi.nlm.nih.gov/nucleotide/KF228163.1?report=genbank&log$=nucltop&blast_rank=88&RID=3AU3G8Y6013) | [*Chitinophaga*](https://www.ncbi.nlm.nih.gov/Taxonomy/Browser/wwwtax.cgi?mode=Undef&id=79328&lvl=3&keep=1&srchmode=1&unlock) | [Chitinophagaceae](https://www.ncbi.nlm.nih.gov/Taxonomy/Browser/wwwtax.cgi?mode=Undef&id=563835&lvl=3&keep=1&srchmode=1&unlock) | [Chitinophagales](https://www.ncbi.nlm.nih.gov/Taxonomy/Browser/wwwtax.cgi?mode=Undef&id=1853229&lvl=3&keep=1&srchmode=1&unlock) | [Chitinophagia](https://www.ncbi.nlm.nih.gov/Taxonomy/Browser/wwwtax.cgi?mode=Undef&id=1853228&lvl=3&keep=1&srchmode=1&unlock) | [Bacteroidetes](https://www.ncbi.nlm.nih.gov/Taxonomy/Browser/wwwtax.cgi?mode=Undef&id=976&lvl=3&keep=1&srchmode=1&unlock) | [Bacteria](https://www.ncbi.nlm.nih.gov/Taxonomy/Browser/wwwtax.cgi?mode=Undef&id=2&lvl=3&keep=1&srchmode=1&unlock) |
| 124 | 100% | *0* | ***Owenweeksia sp*** | [KT826315.1](https://www.ncbi.nlm.nih.gov/nucleotide/KT826315.1?report=genbank&log$=nucltop&blast_rank=6&RID=3AUNH9BJ01R) | [*Owenweeksia*](https://www.ncbi.nlm.nih.gov/Taxonomy/Browser/wwwtax.cgi?mode=Undef&id=267986&lvl=3&keep=1&srchmode=1&unlock) | [Schleiferiaceae](https://www.ncbi.nlm.nih.gov/Taxonomy/Browser/wwwtax.cgi?mode=Undef&id=1333713&lvl=3&keep=1&srchmode=1&unlock) | [Flavobacteriales](https://www.ncbi.nlm.nih.gov/Taxonomy/Browser/wwwtax.cgi?mode=Undef&id=200644&lvl=3&keep=1&srchmode=1&unlock) | [Flavobacteriia](https://www.ncbi.nlm.nih.gov/Taxonomy/Browser/wwwtax.cgi?mode=Undef&id=117743&lvl=3&keep=1&srchmode=1&unlock) | [Bacteroidetes](https://www.ncbi.nlm.nih.gov/Taxonomy/Browser/wwwtax.cgi?mode=Undef&id=976&lvl=3&keep=1&srchmode=1&unlock) | [Bacteria](https://www.ncbi.nlm.nih.gov/Taxonomy/Browser/wwwtax.cgi?mode=Undef&id=2&lvl=3&keep=1&srchmode=1&unlock) |
| 125 | 95.49% | *0* | ***Owenweeksia sp*** | [KT826315.1](https://www.ncbi.nlm.nih.gov/nucleotide/KT826315.1?report=genbank&log$=nucltop&blast_rank=2&RID=3AUVKVAA01R) | [*Owenweeksia*](https://www.ncbi.nlm.nih.gov/Taxonomy/Browser/wwwtax.cgi?mode=Undef&id=267986&lvl=3&keep=1&srchmode=1&unlock) | [Schleiferiaceae](https://www.ncbi.nlm.nih.gov/Taxonomy/Browser/wwwtax.cgi?mode=Undef&id=1333713&lvl=3&keep=1&srchmode=1&unlock) | [Flavobacteriales](https://www.ncbi.nlm.nih.gov/Taxonomy/Browser/wwwtax.cgi?mode=Undef&id=200644&lvl=3&keep=1&srchmode=1&unlock) | [Flavobacteriia](https://www.ncbi.nlm.nih.gov/Taxonomy/Browser/wwwtax.cgi?mode=Undef&id=117743&lvl=3&keep=1&srchmode=1&unlock) | [Bacteroidetes](https://www.ncbi.nlm.nih.gov/Taxonomy/Browser/wwwtax.cgi?mode=Undef&id=976&lvl=3&keep=1&srchmode=1&unlock) | [Bacteria](https://www.ncbi.nlm.nih.gov/Taxonomy/Browser/wwwtax.cgi?mode=Undef&id=2&lvl=3&keep=1&srchmode=1&unlock) |
| 126* | 100% | *0* |  | [AF540049.1](https://www.ncbi.nlm.nih.gov/nucleotide/AF540049.1?report=genbank&log$=nucltop&blast_rank=1&RID=3AV0MHJJ016) |  |  |  |  | [Nitrospirae](https://www.ncbi.nlm.nih.gov/Taxonomy/Browser/wwwtax.cgi?mode=Undef&id=40117&lvl=3&keep=1&srchmode=1&unlock) | [Bacteria](https://www.ncbi.nlm.nih.gov/Taxonomy/Browser/wwwtax.cgi?mode=Undef&id=2&lvl=3&keep=1&srchmode=1&unlock) |
| 127 | 97.8% | *0* | ***Candidatus Planktophila vernalis*** | [CP016776.1](https://www.ncbi.nlm.nih.gov/nucleotide/CP016776.1?report=genbank&log$=nucltop&blast_rank=3&RID=3AV7U2EY01R) | [*Candidatus Planktophila*](https://www.ncbi.nlm.nih.gov/Taxonomy/Browser/wwwtax.cgi?mode=Undef&id=622681&lvl=3&keep=1&srchmode=1&unlock) | [Candidatus Nanopelagicaceae](https://www.ncbi.nlm.nih.gov/Taxonomy/Browser/wwwtax.cgi?mode=Undef&id=2162846&lvl=3&keep=1&srchmode=1&unlock) | [Candidatus Nanopelagicales](https://www.ncbi.nlm.nih.gov/Taxonomy/Browser/wwwtax.cgi?mode=Undef&id=2039638&lvl=3&keep=1&srchmode=1&unlock) | [Actinomycetia](https://www.ncbi.nlm.nih.gov/Taxonomy/Browser/wwwtax.cgi?mode=Undef&id=1760&lvl=3&keep=1&srchmode=1&unlock) | [Actinobacteria](https://www.ncbi.nlm.nih.gov/Taxonomy/Browser/wwwtax.cgi?mode=Undef&id=201174&lvl=3&keep=1&srchmode=1&unlock) | [Bacteria](https://www.ncbi.nlm.nih.gov/Taxonomy/Browser/wwwtax.cgi?mode=Undef&id=2&lvl=3&keep=1&srchmode=1&unlock) |
| 128* | 94.56% | *0* |  | [CP054186.1](https://www.ncbi.nlm.nih.gov/nucleotide/CP054186.1?report=genbank&log$=nucltop&blast_rank=4&RID=3AVHKYST013) |  | [Phycisphaeraceae](https://www.ncbi.nlm.nih.gov/Taxonomy/Browser/wwwtax.cgi?mode=Undef&id=666507&lvl=3&keep=1&srchmode=1&unlock) | [Phycisphaerales](https://www.ncbi.nlm.nih.gov/Taxonomy/Browser/wwwtax.cgi?mode=Undef&id=666506&lvl=3&keep=1&srchmode=1&unlock) | [Phycisphaerae](https://www.ncbi.nlm.nih.gov/Taxonomy/Browser/wwwtax.cgi?mode=Undef&id=666505&lvl=3&keep=1&srchmode=1&unlock) | [Planctomycetes](https://www.ncbi.nlm.nih.gov/Taxonomy/Browser/wwwtax.cgi?mode=Undef&id=203682&lvl=3&keep=1&srchmode=1&unlock) | [Bacteria](https://www.ncbi.nlm.nih.gov/Taxonomy/Browser/wwwtax.cgi?mode=Undef&id=2&lvl=3&keep=1&srchmode=1&unlock) |
| 129* | 100% | *0* |  | [AF540054.1](https://www.ncbi.nlm.nih.gov/nucleotide/AF540054.1?report=genbank&log$=nucltop&blast_rank=2&RID=3AVSSF9401R) |  |  |  |  | [Nitrospirae](https://www.ncbi.nlm.nih.gov/Taxonomy/Browser/wwwtax.cgi?mode=Undef&id=40117&lvl=3&keep=1&srchmode=1&unlock) | [Bacteria](https://www.ncbi.nlm.nih.gov/Taxonomy/Browser/wwwtax.cgi?mode=Undef&id=2&lvl=3&keep=1&srchmode=1&unlock) |
| 130* | 99% | *0* |  | [EF663626.1](https://www.ncbi.nlm.nih.gov/nucleotide/EF663626.1?report=genbank&log$=nucltop&blast_rank=8&RID=3AVZ62DR01R) |  |  |  |  | [Acidobacteria](https://www.ncbi.nlm.nih.gov/Taxonomy/Browser/wwwtax.cgi?mode=Undef&id=57723&lvl=3&keep=1&srchmode=1&unlock) | [Bacteria](https://www.ncbi.nlm.nih.gov/Taxonomy/Browser/wwwtax.cgi?mode=Undef&id=2&lvl=3&keep=1&srchmode=1&unlock) |
| 131* | 100% | *0* | ***Escherichia coli*** | [MT263026.1](https://www.ncbi.nlm.nih.gov/nucleotide/MT263026.1?report=genbank&log$=nucltop&blast_rank=1&RID=3AW381C701R) | [*Escherichia*](https://www.ncbi.nlm.nih.gov/Taxonomy/Browser/wwwtax.cgi?mode=Undef&id=561&lvl=3&keep=1&srchmode=1&unlock) | [Enterobacteriaceae](https://www.ncbi.nlm.nih.gov/Taxonomy/Browser/wwwtax.cgi?mode=Undef&id=543&lvl=3&keep=1&srchmode=1&unlock) | [Enterobacterales](https://www.ncbi.nlm.nih.gov/Taxonomy/Browser/wwwtax.cgi?mode=Undef&id=91347&lvl=3&keep=1&srchmode=1&unlock) | [Gammaproteobacteria](https://www.ncbi.nlm.nih.gov/Taxonomy/Browser/wwwtax.cgi?mode=Undef&id=1236&lvl=3&keep=1&srchmode=1&unlock) | [Proteobacteria](https://www.ncbi.nlm.nih.gov/Taxonomy/Browser/wwwtax.cgi?mode=Undef&id=1224&lvl=3&keep=1&srchmode=1&unlock) | [Bacteria](https://www.ncbi.nlm.nih.gov/Taxonomy/Browser/wwwtax.cgi?mode=Undef&id=2&lvl=3&keep=1&srchmode=1&unlock) |
| 132* | 100% | *0* | ***Escherichia coli*** | [AB075685.1](https://www.ncbi.nlm.nih.gov/nucleotide/AB075685.1?report=genbank&log$=nucltop&blast_rank=1&RID=3AW97TA101R) | [*Escherichia*](https://www.ncbi.nlm.nih.gov/Taxonomy/Browser/wwwtax.cgi?mode=Undef&id=561&lvl=3&keep=1&srchmode=1&unlock) | [Enterobacteriaceae](https://www.ncbi.nlm.nih.gov/Taxonomy/Browser/wwwtax.cgi?mode=Undef&id=543&lvl=3&keep=1&srchmode=1&unlock) | [Enterobacterales](https://www.ncbi.nlm.nih.gov/Taxonomy/Browser/wwwtax.cgi?mode=Undef&id=91347&lvl=3&keep=1&srchmode=1&unlock) | [Gammaproteobacteria](https://www.ncbi.nlm.nih.gov/Taxonomy/Browser/wwwtax.cgi?mode=Undef&id=1236&lvl=3&keep=1&srchmode=1&unlock) | [Proteobacteria](https://www.ncbi.nlm.nih.gov/Taxonomy/Browser/wwwtax.cgi?mode=Undef&id=1224&lvl=3&keep=1&srchmode=1&unlock) | [Bacteria](https://www.ncbi.nlm.nih.gov/Taxonomy/Browser/wwwtax.cgi?mode=Undef&id=2&lvl=3&keep=1&srchmode=1&unlock) |

| **Table S6. Bacterial species that have demonstrated resistance to antibiotics and that were detected in wastewater samples by metagenomics.** | | | |
| --- | --- | --- | --- |
| **Specie** | **Isolation habitat** | **Antibiotic to which it is resistant (concentration)** | **References** |
| *Prolixibacter sp.* | Residual water | Amoxicillin | [1] |
| *Fusobacterium sp* | Colon of patient | Penicillin (0.25 µg); amoxicillin (0.12) µg; imipenem (0.25 µg); metronidazole (l0.25 µg). | [2] |
| *Vibrio parahaemolyticus* | Isolated from retail markets in Cochin India | Cefepime tetracycline, aztreonam, streptomycin, gentamicin tobramycin | [3] |
| *[Enterobacter cloacae](https://www.ncbi.nlm.nih.gov/Taxonomy/Browser/wwwtax.cgi?mode=Info&id=550&lvl=3&lin=f&keep=1&srchmode=1&unlock)* | Patients in hospitals | Piperacillin (100 µg); trimethoprim (2.5 µg); gentamicin (10 µg); ciprofloxacin (5 µg); ceftazidine (30 µg); ampicillin (10 µg), erythromycin (5 µg); clindamycin (2 µg); cephaloridin (5 µg); tetracycline (10 µg); and penicillin G (1 ug). | [4] |
| [*Escherichia coli*](https://www.ncbi.nlm.nih.gov/Taxonomy/Browser/wwwtax.cgi?mode=Info&id=562&lvl=3&lin=f&keep=1&srchmode=1&unlock) | Residual water | Ampicillin (32 µg/mL); chloramphenicol 32 µg/mL; sulfamethoxazole (512 µg/mL); tetracycline (16 µg/mL); trimethoprim (10 µg/mL); streptomycin (64 µg/mL); kanamycin 64 (µg/mL) and nalidixic acid (64 µg/mL). | [5] |
| *Sphingomonas faucium* | Mire of the Enshi Grand Canyon | Sensitive to carbenicillin, erythromycin, norfloxacin, ofloxacin, penicillin, piperacillin, ceftriaxone, doxycycline, minocycline, IV cephalosporin, cephalosporin VI, cefoperazone, neomycin, sulfamethoxazole, cephalosporin V, cefonazidine, and resistant but methofonazidine, cephalosporin V, cefonaximazidine, and resistant oximezidurazidine | [6] |
| ***[Pseudomonas alcaligenes](https://www.ncbi.nlm.nih.gov/Taxonomy/Browser/wwwtax.cgi?mode=Info&id=43263&lvl=3&lin=f&keep=1&srchmode=1&unlock)*** | Municipal water supply and water sources | Sulfanilamide (350 µg/mL); streptomycin sulfate (15 µg/mL); kanamycin sulfate (25 µg/mL); chloramphenicol (25 µg/mL) and tetracycline hydrochloride (12 µg/mL). | [7] |
| ***[Pseudomonas protegens](https://www.ncbi.nlm.nih.gov/Taxonomy/Browser/wwwtax.cgi?mode=Info&id=380021&lvl=3&lin=f&keep=1&srchmode=1&unlock)*** | Residual water | Amicamycin (16 mg/L); Gentamincin (4 mg/L); Trobamycin (4 mg/L); Imipenem (8 mg/L) and Meropenem (8 mg/L). | [8] |
| ***Pseudomonas syringae*** | Ninety-five strains of P. syringae were analyzed. | Ampicillin (100 g/mL); chloramphenicol (25 g/mL); kanamycin (50 g/mL); rifampin (50 g/mL); streptomycin (100 g/mL), or tetracycline (15 g/mL). | [9] |
| ***Stenotrophomonas acidaminiphila*** | Reactor that treats petrochemicals in  wastewater in  Upflow Anaerobic Mud Blanket (UASB) | Amikacin (30 µg); gentamicin (10 IU); netilmycin (30 µg); tobramycin (10 µg); fluoroquinolones (10ug). | [10] |
| ***Methylophilus sp.*** | Two-chamber microbial electrochemical reactors manufactured  acrylic glass biofilm | Sulfamethoxazole | [11] |
| ***Bdellovibrio bacteriovorus*** | n.d. | Amikamycin (0.7 µg/mL); kanamycin (0.69 µg/mL), gentamicin (0.06 µg/mL); tetracycline (1.7 µg/mL). | [12] |
| ***Kluyvera ascorbata*** | Recombinant strain | Cefotaxime (0.5 and 2 µg/mL); amoxicillin (100 µg/mL); tetracycline (15 µg/mL); kanamycin (30 µg/mL) and gentamicin (7 µg/mL). | [13] |
| ***Acinetobacter baumannii*** | Isolated samples from hospitals in Korea | Colistin (32 g/L); tetracycline (64 g/L): cefepime (64 g/L) and ampicillin (64 g/L). | [14] |
| ***Bacillus coreaensis*** | Poultry waste from poultry farm in Namakkal district, Tamil Nadu, India. | Ampicillin (35); cloxacillin, chloramphenicol, amoxicillin and ciprofloxacin (15%); tetracycline (50%); oxytetracycline (80%) and oxytetracycline. and erythromycin (90) | [15] |
| ***Sphingobacterium sp.*** | *Sphingobacterium* sp. PM2‐P1‐29 | Tetracycline (256 mg L^-1^); oxytetracycline (128 mg L^-1^); chlortetracycline (64 mg L^-1^) and doxycycline (32 mg L ^– 1^). | [16] |
| ***Moraxella osloensis*** | Brucellosis patients | Penicilina G (6.25 µg / mL), ampicilina (3,12 µg/mL) and tetraciclina (0.39 µg/mL). | [17] |
| ***Gallionella capsiferriformans*** | Biofilm from a reactor | Tetracycline | [18] |
| ***Methylobacter sp.*** | Hospital tap water | Ampicillin (120 µg/mL-256 µg/mL); cefuroxime (128 µg/mL-256 µg/mL); gentamicin (16 µg/mL-128 µg/mL); erythromycin (4 µg/mL-256 µg/mL); vancomycin (256 µg/mL-256 ug/mL); tetracycline (2 µg/mL-8 µg/ mL); chloramphenicol (256 µg/mL-256 µg/mL) | [19] |
| ***Magnetobacterium sp*** | Water from a pig farm | Oxytetracycline and Chlortetracycline | [20] |
| ***Propionibacterium sp*** | Samples of patients in hospitals | Amoxicillin (≤ 0.5 mg/L); ceftriaxone (<16 mg/L); vancomycin (≤ 2 mg/L); daptomycin (2 mg/L); linezolid (≤ 4 mg/L); rifampicin (≤ 0.5 mg/L); erythromycin (≤ 0.5 mg/ L); clindamycin (≤2 mg/L); moxifloxacin (≤2 mg/L) and tetracycline (≤ 4 mg/L). | [21] |
| ***Paenibacillus sp.*** | Samples from children with acute lymphoblastic leukemia  who were hospitalized at First Pavlov State Medical University, Saint Petersburg, Russia | Amoxiclav (30 μg); ampicillin (10 μg); penicillin (10 U); vancomycin (30 μg); cefotaxime (30 μg); erythromycin (10 μg); azithromycin (15 μg); gentamicin (10 μg); amikacin (30 μg); kanamycin (30 μg); clindamycin (2 μg); doxycycline (30 μg); ciprofloxacin (5 μg); neomycin (30 μg); chloramphenicol (30 μg); tetracycline (30 μg) and trimethoprim-sulfamethoxazole (1.25 μg/23.75 μg). | [22] |
| ***Acinetobacter pittii*** | Drinking water treatment plant | Amoxicillin (25 µg); gentamicin (10 μg); ciprofloxacin (5 μg); sulfamethoxazole/trimethoprim (23.75/1.25 μg); tetracycline (30 μg); cephalothin (30 μg); meropenem (10 μg); ceftazidime (30 μg); ticarcillin (75 μg); colistin sulfate (50 μg); sulfamethoxazole (25 μg); and streptomycin (10 μg). | [23] |
| ***Caulobacter sp*** | 64-year-old patient | Ampicillin (256 μg/mL); amoxicillin (16 μg/mL); streptomycin (3 μg/mL), tetracycline (0.094 μg/mL); vancomycin (24 μg/mL). | [24] |
| ***Aeromonas veronii*** | Clinical isolates | Ampicillin (32 μg/mL); cefepime (32 μg/mL) and gentamicin (16 μg/mL). | [25] |
| ***Staphylococcus epidermidis*** | Simples of hospital | Ciprofloxacin (32 μg/mL- 64 μg/mL) | [26] |
| ***Pseudomonas alcaliphila*** | Cepa isolated in 2009. | Ampicillin (10 μg); chloramphenicol  (30 μg); oxytetracycline (30 μg); erythromycin (15 μg);  penicillin (10 μg) and carbenicillin (100 μg). | [27] |
| ***Neisseria mucosa*** | Piercing a patient | penicillin G (0.38 mg/L) | [28] |
| ***Aeromonas caviae*** | Clinical isolates | Gentamicin (4-16 μg/mL) | [25] |
| ***Shewanella algae*** | Samples obtained from a bronchoalveolar lavage of a hospitalized  patient at the Timone Hospital (Marseille, France). | Kanamycin (50 mg/L) and Colistin (8 mg/L) | [29] |
| ***Streptococcus pyogenes*** | Isolated skin | Erythromycin (8-0.25 mg/mL); penicillin (0.016 mg/mL); cephalothin (0.125 mg/mL); tetracycline (0.5 mg/mL); chloramphenicol (4 mg/mL); ciprofloxacin (0.5 mg/mL) and vancomycin (0.5 mg/mL). | [30] |
| ***Streptococcus agalactiae*** | Patients samples | Ampicillin (0.032-0.064 μg/mL); gentamicin (8-16 μg/mL) and vancomycin (0.25-0.5 μg/mL) | [31] |
| ***Streptococcus parauberis*** | Isolates from olives | Erythromycin (256 mg/mL); tetracycline (128 mg/mL; penicillin (0.25 mg/mL). | [32] |
| ***Streptococcus intermedius*** | Endocarditis patient | Ceftriaxona (0.25 μg/mL). | [33] |
| ***Streptococcus pseudoporcinus*** | Immunosuppressed patient | Susceptible to penicillin, erythromycin, clindamycin, vancomycin, and trimethoprim-sulfamethoxazole; and especially tetracycline. | [34] |
| ***Burkholderia cepacia*** |  | Polymix B | [35] |
| ***Klebsiella pneumoniae*** |  | β-lactams, aminoglycosides, quinolones,  tigecycline and polymyxins | [36] |
| ***Aeromonas salmonicida*** | Canadá | Chloramphenicol/florfenicol, tetracycline, and sulfamethoxazole | [37] |
| ***Vibrio chagasii*** | Diseased hybrids (E. fuscoguttatus ♀ 9 E. lanceolatus ♂),  who presented scales fall and necrotic muscle symptoms, were collected from Danzhou and Wenchang cities | Penicillin (93.3%); ampicillin (93.3%); oxacillin (100%); amoxicillin (63.3%); carbenicillin (93.3%), cephalosporins and other b-lactams such as cefoxitin, imipenem, amoxicillin/clavulanic acid, latamoxef, aztreonam and quinolones. | [38] |

[1 ]Meng L W, Li XK, Wang K, Ma K L, Zhang J, et al. Influence of the amoxicillin concentration on organics removal and microbial community structure in an anaerobic EGSB reactor treating with antibiotic wastewater. Chem Eng J. 2015; 274: 94-101. <https://doi.org/10.1016/j.cej.2015.03.065>

[2] Shilnikova, II, Dmitrieva, NV. Evaluation of antibiotic susceptibility of Bacteroides, Prevotella and Fusobacterium species isolated from patients of the NN Blokhin Cancer Research Center, Moscow, Russia. Anaerobe.  2015;31 : 15-18. <https://doi.org/10.1016/j.anaerobe.2014.08.003>

[3 Elmahdi, S, DaSilva LV, Parveen S. Antibiotic resistance of Vibrio parahaemolyticus and Vibrio vulnificus in various countries: a review. Food Microbiol. 2016;57: 128-134. <https://doi.org/10.1016/j.fm.2016.02.008>

[4] Davis, IJ, Richards H, Mullany P. Isolation of silver‐and antibiotic‐resistant Enterobacter cloacae from teeth. Oral Microbiol Immun. 2005;20 (3): 191-194.  <https://doi.org/10.1111/j.1399-302X.2005.00218.x>

[5] Pignato S, Coniglio MA, Faro G, Lefevre M, Weill FX, Giammanco G. Molecular epidemiology of ampicillin resistance in Salmonella spp. and Escherichia coli from wastewater and clinical specimens. Foodborne Pathog Dis. 2010;7 (8): 945-951. <https://doi.org/10.1089/fpd.2009.0504>

[6] Liu D, Jin, X, Sun X, Song Y, Feng L, Wang G , et al. Sphingomonas faucium sp. nov., isolated from canyon soil. Int. J. Syst. Evol. Microbiol. 2016;66 (8): 2847-2852. <https://doi.org/10.1099/ijsem.0.001064>

[7] Armstrong JL, Shigeno DS, Calomiris JJ, Seidler RJ. Antibiotic-resistant bacteria in drinking water. *Applied and* Environ Microbio. 1981;42 (2) : 277-283. : <https://doi.org/10.1128/aem.42.2.277-283.1981>

[8] Luczkiewicz A, Kotlarska E, Artichowicz W, Tarasewicz K, Fudala-Ksiazek S. Antimicrobial resistance of Pseudomonas spp. isolated from wastewater and wastewater-impacted marine coastal zone. Environ Sci Pollut Res. 2015;22 (24): 19823-19834. DOI: 10.1007/s11356-015-5098-y

[9] Hwang MS, Morgan RL, Sarkar SF, Wang PW, Guttman DS. Phylogenetic characterization of virulence and resistance phenotypes of Pseudomonas syringae. Appl Environ Microbiol. 2005;71 (9): 5182-5191.  <https://doi.org/10.1128/AEM.71.9.5182-5191.2005>

[10] Assih EA, Ouattara AS, Thierry S, Cayol JL, Labat M, Macarie H. Stenotrophomonas acidaminiphila sp. nov., a strictly aerobic bacterium isolated from an upflow anaerobic sludge blanket (UASB) reactor.  Int. J Syst Evol Microbiol. 2002;52 (2): 559-568. <https://doi.org/10.1099/00207713-52-2-559>

[11] Yan W, Bai R, Wang S, Tian X, Li Y, Wang S, et al. Antibiotic resistance genes are increased by combined exposure to sulfamethoxazole and naproxen but relieved by low-salinity. Environ int. 2020;139: 105742. <https://doi.org/10.1016/j.envint.2020.105742>

[12] Dashiff A, Junka R A, Libera M, Kadouri DE. Predation of human pathogens by the predatory bacteria Micavibrio aeruginosavorus and Bdellovibrio bacteriovorus. J. Appl. Microbiol. 2011;110 (2): 431-444. <https://doi.org/10.1111/j.1365-2672.2010.04900.x>

[13] Lartigue MF, Poirel L, Aubert D, Nordmann P. In vitro analysis of IS Ecp1B-mediated mobilization of naturally occurring β-lactamase gene bla CTX-M of Kluyvera ascorbata. Antimicrob. Agents Chemother. 2006;50(4):1282-1286. <https://doi.org/10.1128/AAC.50.4.1282-1286.200>

[14] Ko KS, Suh J Y, Kwon KT, Jung SI, Park KH, Kang C I , et al. High rates of resistance to colistin and polymyxin B in subgroups of Acinetobacter baumannii isolates from Korea. J Antimicrob Chemother. 2007;60(5):1163-1167. <https://doi.org/10.1093/jac/dkm305>

[15] Boovaragamoorthy GM, Anbazhagan M, Piruthiviraj P, Pugazhendhi A, Kumar SS, Al-Dhabi NA , et al. Clinically important microbial diversity and its antibiotic resistance pattern towards various drugs. J Infect Public Health 2019;12(6): 783-788. <https://doi.org/10.1016/j.jiph.2019.08.008>

[16] Ghosh S, Sadowsky MJ, Roberts MC, Gralnick JA, LaPara, TM. Sphingobacterium sp. strain PM2‐P1‐29 harbours a functional tet (X) gene encoding for the degradation of tetracycline.  J Appl Microbiol*.* 2009;104 : 1336-1342.

<https://doi.org/10.1111/j.1365-2672.2008.04101.x>

[17] Hansen W, Butzler JP, Fuglesang JE, Henriksen SD. Isolation of penicillin and streptomycin resistant strains of Moraxella osloensis. Acta Patho Microbiol Scand. 1974;82 (3): 318-322. <https://doi.org/10.1111/j.1699-0463.1974.tb02333.x>

[18] Taşkan B, Casey E, Hasar H. Simultaneous oxidation of ammonium and tetracycline in a membrane aerated biofilm reactor. Sci Total Environ. 2019;682: 553-560. <https://doi.org/10.1016/j.scitotenv.2019.05.111>

[19] Uruhata K, Kato Y, Goto K, Hara M, Yoshida SI, Fukuyama M. Isolation and identification of Methylobacterium species from the tap water in hospitals in Japan and their antibiotic susceptibility. Microbiol immunolo. 2006;50 (1): 11-17. <https://doi.org/10.1111/j.1348-0421.2006.tb03765.x>

[20] Chen J, Yang Y, Liu Y, Tang M, Wang R, Hu H, et al. Effects caused by chlortetracycline and oxytetracycline in anaerobic digestion treatment of real piggery wastewater: Treatment efficiency and bacterial diversity. Int. J Hydrog Energy. 2020;45 (15): 9222-9230. <https://doi.org/10.1016/j.ijhydene.2020.01.138>

[21] Broly M, d’Epenoux LR, Guillouzouic A, Le Gargasson G, Juvin ME , Leroy AG, et al. Propionibacterium/Cutibacterium species–related positive samples, identification, clinical and resistance features: a 10-year survey in a French hospital. Eur J of Clin Microbio Infect Dis. 2020;39 (7): 1357-1364.
https://doi.org/10.1007/s10096-020-03852-5

[22] Tetz G, Tetz V, Vecherkovskaya M. Genomic characterization and assessment of the virulence and antibiotic resistance of the novel species Paenibacillus sp. strain VT-400, a potentially pathogenic bacterium in the oral cavity of patients with hematological malignancies. Gut Pathog. 2016;8 (1): 1-9. <https://doi.org/10.1186/s13099-016-0089-1>

[23] Narciso-da-Rocha C, Vaz-Moreira I, Svensson-Stadler L, Moore E R, Manaia CM. Diversity and antibiotic resistance of Acinetobacter spp. in water from the source to the tap.  **Appl Microbiol Biotechnol.** 2013;97(1):329-340.
https://doi.org/10.1007/s00253-012-4190-1

[24] Justesen US, Holt HM, Thiesson HC, Blom J, Nielsen XC, Dargis R, et al. Report of the first human case of Caulobacter sp. infection. J. Clin. Microbiol. 2007;45(4):1366-1369.  <https://doi.org/10.1128/JCM.02380-06>

[25] Vila J, Marco F, Soler L, Chacon M, Figueras MJ. In vitro antimicrobial susceptibility of clinical isolates of Aeromonas caviae, Aeromonas hydrophila and Aeromonas veronii biotype sobria.  J. Antimicrob. Chemother. 2002;49 (4): 701-702. <https://doi.org/10.1093/jac/49.4.701>

[26] Raad I, Alrahwan A, Rolston K. Staphylococcus epidermidis: emerging resistance and need for alternative agents. Rev Infect Dis 1998;26(5):1182-1187. <https://doi.org/10.1086/520285>

[27] Noreen S, Ali B, Hasnain S. Growth promotion of Vigna mungo (L.) by Pseudomonas spp. exhibiting auxin production and ACC-deaminase activity.  Ann Microbiol. 2012;62(1): 411-417. <https://doi.org/10.1007/s13213-011-0277-7>

[28] Tronel H, Chaudemanche H, Pechier N, Doutrelant L, Hoen B. Endocarditis due to Neisseria mucosa after tongue piercing. Clin Microbiol Infect. 2001;7 (5) 275-276.  <https://doi.org/10.1046/j.1469-0691.2001.00241.x>

[29] Telke A A, Rolain JM. Functional genomics to discover antibiotic resistance genes: the paradigm of resistance to colistin mediated by ethanolamine phosphotransferase in Shewanella algae MARS 14. Int J Antimicrob Agents 2015;4 (6): 648-652. <https://doi.org/10.1016/j.ijantimicag.2015.09.001>

[30] Seppälä H, Skurnik M, Soini H, Roberts MC, Huovinen, P. A novel erythromycin resistance methylase gene (ermTR) in Streptococcus pyogenes. Antimicrob agents chemother. 1998;42 (2): 257-262.  <https://doi.org/10.1128/AAC.42.2.257>

[31] Poyart C, Jardy L, Quesne G, Berche P, Trieu-Cuot, P. Genetic basis of antibiotic resistance in Streptococcus agalactiae strains isolated in a French hospital. Antimicrob agents chemother. 2003;47(2):794-797.  <https://doi.org/10.1128/AAC.47.2.794-797.2003>

[32] Park YK, Nho SW, Shin GW, Park SB, Jang HB, Cha IS et al. Antibiotic susceptibility and resistance of Streptococcus iniae and Streptococcus parauberis isolated from olive flounder (Paralichthys olivaceus). Vet microbiol. 2009;136(1-2):76-81. <https://doi.org/10.1016/j.vetmic.2008.10.002>

[33] Cunha BA, D'Elia AA, Pawar N, Schoch P. Viridans streptococcal (Streptococcus intermedius) mitral valve subacute bacterial endocarditis (SBE) in a patient with mitral valve prolapse after a dental procedure: the importance of antibiotic prophylaxis. Heart Lung. 2010;39 (1): 64-72. <https://doi.org/10.1016/j.hrtlng.2009.01.004>

[34] Swain B, Otta S, Bhoi P. Streptococcus pseudoporcinus: a rare cause of bacteraemia in an immune-compromised patient. Int J Res Med Sci*.* 2020;8 (1): 365.  [http://dx.doi.org/10.18203/2320-6012.ijrms20195935](https://dx.doi.org/10.18203/2320-6012.ijrms20195935)

[35 Speert DP. Advances in Burkholderia cepacia complex. Paediatr Respir Rev. 2003;3(3):230-235. <https://doi.org/10.1016/S1526-0542(02)00185-9>

[36] Navon-Venezia S, Kondratyeva K, Carattoli A. Klebsiella pneumoniae: a major worldwide source and shuttle for antibiotic resistance. FEMS Microbiol Rev. 2017;41(3):252-275. <https://doi.org/10.1093/femsre/fux013>

[37] Trudel MV, Vincent AT, Attéré S A, Labbé M, Derome N, Culley A I, Charette SJ. Diversity of antibiotic-resistance genes in Canadian isolates of Aeromonas salmonicida subsp. salmonicida: dominance of pSN254b and discovery of pAsa8. Sci rep. 2016;6(1):1-10. <https://doi.org/10.1038/srep35617>

[38] Zhu ZM, Dong CF, Weng SP, He J G. The high prevalence of pathogenic Vibrio harveyi with multiple antibiotic resistance in scale drop and muscle necrosis disease of the hybrid grouper, Epinephelus fuscoguttatus (♀)× E. lanceolatus (♂), in China. J fish dis. 2018;41(4):589-601.   <https://doi.org/10.1111/jfd.12758>

| **Table S7. Morphology Characterization of isolates** | | | | |
| --- | --- | --- | --- | --- |
| **Strain** | **Results according to morphology in MacConkey agar plates** | **Resultados según la Tincion Gram** | **Morphology observed according to gram stain** | **Growth morphology by plate extension** |
| *E. coli* DH10B | Lac+ | Gram negative | Bacilli in short chains and in pairs | Rough |
| Strain 1 | Lac+ | Gram negative | Bacilli in short chains and in pairs | Rough |
| Strain 2 | Lac+ | Gram negative | Bacilli in short chains and in pairs | Rough |
| Strain 3 | Lac+ | Gram negative | Bacilli in short chains and in pairs | Rough |
| Strain 4 | Lac+ | Gram negative | Bacilli in short chains and in pairs | Rough |
| Strain 5 | Lac+ | Gram negative | Bacilli in short chains and in pairs | Rough |
| Strain 6 | Lac+ | Gram negative | Bacilli in short chains and in pairs | Rough |
| Strain 7 | Lac+ | Gram negative | Bacilli in short chains and in pairs | Smooth |
| Strain 8 | Lac+ | Gram negative | Bacilli in short chains and in pairs | Rough |
| Strain 9 | Lac+ | Gram negative | Bacilli in short chains and in pairs | Rough |
| Strain 10 | Lac+ | Gram negative | Bacilli in short chains and in pairs | Smooth – mucoid |
| Strain 11 | Lac+ | Gram negative | Bacilli in short chains and in pairs | Rough |
| Strain 12 | Lac+ | Gram negative | Bacilli in short chains and in pairs | Rough |
| Strain 13 | Lac+ | Gram negative | Bacilli in short chains and in pairs | Rough |
| Strain 14 | Lac+ | Gram negative | Filamentous forms: Bacilli that grow in the form of fibers | Rough |
| Strain 15 | Lac+ | Gram negative | Filamentous forms: Bacilli that grow in the form of fibers | Rough |
| Strain 16 | Lac+ | Gram negative | Bacilli in short chains and in pairs | Rough |
| Strain 17 | Lac+ | Gram negative | Filamentous forms: Bacilli that grow in the form of fibers | Rough |
| Strain 18 | Lac+ | Gram negative | Bacilli in short chains and in pairs | mucoid |
| Strain 19 | Lac+ | Gram negative | Filamentous forms: Bacilli that grow in the form of fibers | Rough |
| Strain 20 | Lac+ | Gram negative | Bacilli in short chains and in pairs | Smooth |
| Strain 21 | Lac+ | Gram negative | Bacilli in short chains and in pairs | Rough |
| Strain 22 | Lac- | Gram negative | Bacilli in short chains and in pairs | Smooth |


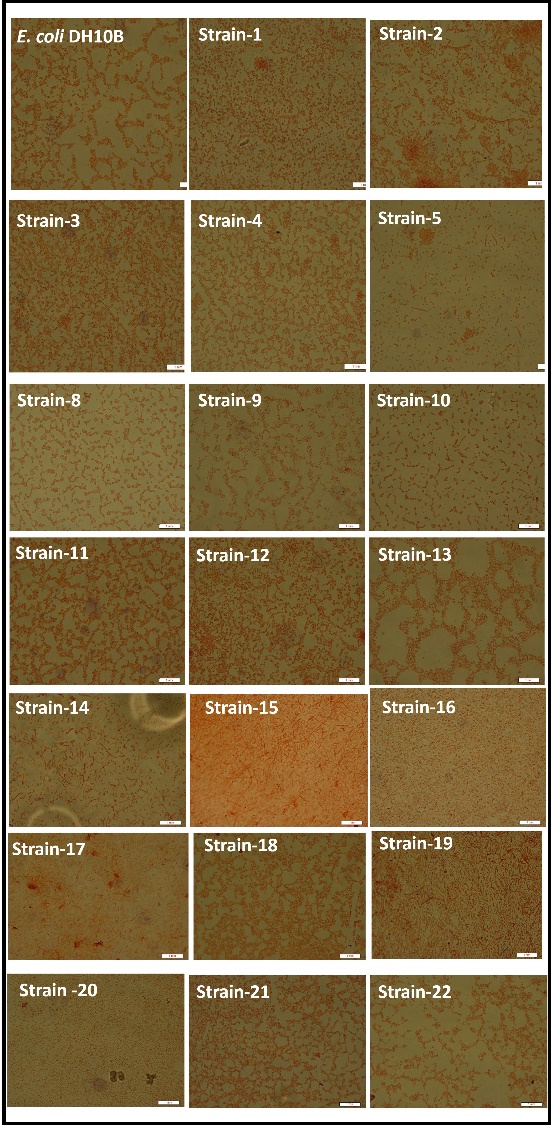


Figure S3. Gram stain of 20 strains and of the control strain *E.coli* DH10B.


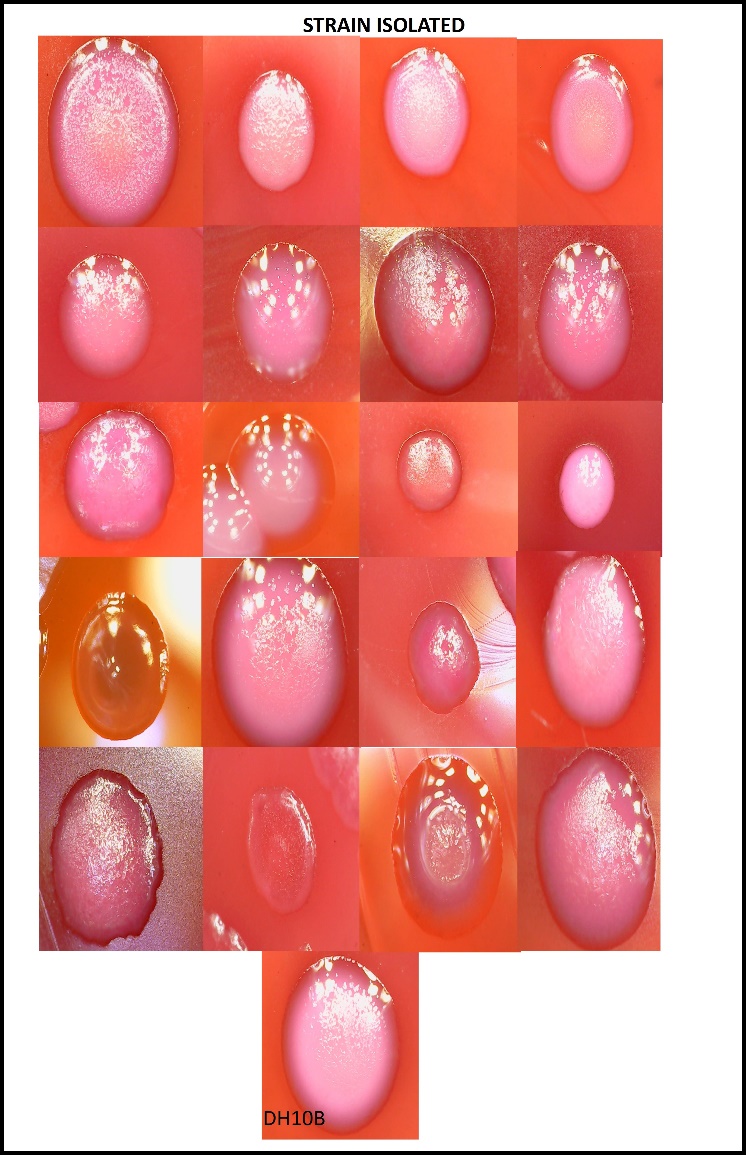


Figure S4. Representation of the morphology observed in the sowing by striatum in MacConkey Agar plates of 20 isolated strains and of the control strain *E.coli* DH10B

| **Table S8. Identification of bacteria by sequencing 16S rDNA** | | | | | |  |
| --- | --- | --- | --- | --- | --- | --- |
| **Strain** | **Size of the analyzed sequence** | **Gene** | **Species** | **Identidy** | **Value E** | |
| *E. coli* DH10B | 465pb | 16S rDNA | *Escherichia Coli* | 100% | 0 | |
| 1 | 340pb | 16S rDNA | *Escherichia Coli* | 73% | 1e^-15^ | |
| 2 | 567pb | 16S rDNA | *Escherichia Coli* | 89% | *1e^-175^* | |
| 3 | 250pb | 16S rDNA | *Escherichia Coli* | 100% | 0 | |
| 4 | 344pb | 16S rDNA | *Escherichia Coli* | 100% | 0 | |
| 5 | 700pb | 16S rDNA | *Escherichia Coli* | 100% | 0 | |
| 6 | 444pb | 16S rDNA | *Escherichia Coli* | *97%* | *4e^-06^* | |
| 7 | 532pb | 16S rDNA | *Escherichia Coli* | 99.45% | 0 | |
| 8 | 342pb | 16S rDNA | *Escherichia Coli* | *84%* | *1e^-596^* | |
| 9 | 283pb | 16S rDNA | *Escherichia Coli* | 100% | 0 | |
| 10 | 659pb | 16S rDNA | *Escherichia Coli* | 100% | 0 | |
| 11 | 325pb | 16S rDNA | *Escherichia Coli* | 100% | 0 | |
| 12 | 244pb | 16S rDNA | *Escherichia Coli* | 100% | 0 | |
| 13 | 511pb | 16S rDNA | *Escherichia Coli* | 100% | 0 | |
| 14 | 423pb | 16S rDNA | *Bacteroides fragilis* | 99.60% | 0 | |
| 15 | 357pb | 16S rDNA | *Bacteroides fragilis* | 99.73% | 0 | |
| 16 | 234pb | 16S rDNA | *Escherichia coli* | 100% | 0 | |
| 17 | 344pb | 16S rDNA | *Bacteroides fragilis* | 99.53% | 0 | |
| 18 | 273pb | 16S rDNA | *Escherichia coli* | 100% | 0 | |
| 19 | 468pb | 16S rDNA | *Bacteroides* *fragilis* | 99.39% | 0 | |
| 20 | 521pb | 16S rDNA | *Escherichia coli* | 100% | 0 | |
| 21 | 680pb | 16S rDNA | *Escherichia coli* | 100% | 0 | |
| 22 | 372pb | 16S rDNA | *Salmonella typhi* | 98.10% | 0 | |
